# Disruption of LTBP4 Inhibition-Induced TGFβ1 Activation Promoted Cell Proliferation and Metastasis in Skin Melanoma by Inhibiting the Activation of the Hippo-YAP1 Signaling Pathway

**DOI:** 10.3389/fcell.2021.673904

**Published:** 2022-02-17

**Authors:** Lina Wang, Dongrun Tang, Tong Wu, Fengyuan Sun

**Affiliations:** ^1^ Sichuan Eye Hospital, AIER Eye Hospital Group, Chengdu, China; ^2^ Tianjin International Joint Research and Development Centre of Ophthalmology and Vision Science, Eye Institute and School of Optometry, Tianjin Medical University Eye Hospital, Tianjin, China

**Keywords:** Ltbp4, proliferation, metastasis, melanoma, hippo, Yap1

## Abstract

Melanoma is a malignant tumor derived from melanocytes, which is the most fatal skin cancer. The present study aimed to explore and elucidate the candidate genes in melanoma and its underlying molecular mechanism. A total of 1,156 differentially expressed genes were obtained from the GSE46517 dataset of Gene Expression Omnibus database using the package “limma” in R. Based on two algorithms (LASSO and SVM-RFE), we obtained three candidate DEGs (LTBP4, CDHR1, and MARCKSL1). Among them, LTBP4 was identified as a diagnostic marker of melanoma (AUC = 0.985). Down-regulation of LTBP4 expression was identified in melanoma tissues and cells, which predicted poor prognosis of patients with melanoma. Cox analysis results discovered that LTBP4 with low expression was an independent prognostic factor for overall survival in patients with melanoma. LTBP4 inhibition reduced cell apoptosis and promoted cell proliferation and metastasis. These changes were correlated with the expression levels of caspase-3, Ki67 and E-cadherin. Further, as indicated by tumor formation study of nude mice, LTBP4 silencing improved the tumorigenic ability of melanoma cells. Knockdown of LTBP4 increased the percentage of active TGFβ1 secreted by melanoma cells. CTGF, Gyr61, and Birc5 expression levels were reduced, YAP1 phosphorylation was inhibited, and YAP1 was translocated from the cytoplasm to the nucleus in melanoma cells treated with TGF-β1. These effects were reversed by LTBP4 overexpression. As evidenced by immunofluorescent staining, Western blotting and luciferase reporter assay, LTBP4 overexpression activated the Hippo signaling pathway, which was characterized by the increased nuclear-cytoplasmic translocation of YAP1 and the enhanced phosphorylation of YAP1, MST1, and MOB1. In addition, the effects of LTBP4 overexpression on inhibiting CTGF, Cyr61 and Birc5 expression, promoting the apoptosis, and inhibiting the metastasis and proliferation of melanoma cells were reversed by the overexpression of YAP1 or MST1. In conclusion, the LTBP4-TGFβ1-Hippo-YAP1 axis is a critical pathway for the progression of skin melanoma.

## Background

Melanoma is a malignant tumor that originates from melanocytes in the skin and other tissues (Dong et al., 2020). Malignant melanoma is the most invasive skin cancer. Surgery is an effective treatment for early melanoma, however, many patients with advanced melanoma are associated with dismal prognosis and are not the candidates for surgery. For these patients, immunotherapy, radiation therapy, chemotherapy, or combination therapy (Altonsy et al., 2020). Therefore, it is very important to identify the effective potential targets and explore its related molecular mechanisms for the diagnosis and treatment of skin melanoma.

Latent transforming growth factor binding protein (LTBP) is an extracellular matrix (ECM) glycoprotein belonging to the LTBP/fibrillins superfamily, which mainly includes four subtypes of LTBP1, LTBP2, LTBP3, and LTBP4 (Koli et al., 2008; Rifkin et al., 2018). LTBP1 and LTBP3 are lowly expressed in epithelial cancer, ovarian cancer (Higashi et al., 2001), and liver cancer (Hou et al., 2017; Deryugina et al., 2018), respectively. LTBP2 functions as a component of ECM microfibers, which plays a role in cell adhesion (Vehviläinen et al., 2003; Enomoto et al., 2018; Pang et al., 2020). Studies have shown that there is a molecular interaction between LTBP4 and TGFβR2, and LTBP4 gene knockdown reduces the abundance of TGFβ receptor, resulting in the increased TGF-β1 level (Su et al., 2015; Ruan et al., 2019; Cao et al., 2020). It has also been shown that active TGF-β1 affects the activation of Hippo signaling pathway and the function of YAP1, thereby regulating the proliferation of tumor cells (Patel et al., 2019). The knockdown of LTBP4 can increase the percentage of active TGF-β1 secreted by liver cancer cell lines (Yang et al., 2020). The Hippo signaling pathway, which is involved in regulating the homeostasis of various cells and organs, is related to the occurrence and development of melanoma (Zhang et al., 2020). The hippo signaling pathway is mainly composed of a series of kinase complexes and transcription activating factors, including MST1/2, SAV1, LATS1/2, MOB1, and YAP1/TAZ. In addition, the Hippo pathway oncoprotein YAP can promote cell invasion and spontaneous metastasis in skin melanoma (Nallet-Staub et al., 2014; Zhang et al., 2019). Our previous results showed that LTBP4 was not only significantly down-regulated in melanoma tumor tissues, but also served as a diagnostic marker for melanoma in the GSE46517 dataset (Gutzmer et al., 2020). Therefore, we hypothesized that LTBP4 was a gene closely related to the occurrence and development of skin melanoma, and explored its related molecular mechanisms in this study.

## Methods

### Melanoma Cancer Database and Data Processing

GSE46517 dataset containing mRNA expression profiles were downloaded from the Gene Expression Omnibus (GEO) database (http://www.ncbi.nlm.nih.gov/geo/). GSE46517 dataset included 121 samples, which among is seven normal skin samples, one normal epithelial melanocytes sample, and 113 melanoma samples (73 metastatic melanoma samples and 40 primary melanoma samples). Because normal epithelial melanocytes sample does not belong to tissue sample, seven normal skin samples were included in the control group and 113 melanoma samples were included in the experiment group in this study.

### Data Processing and DEG Screening

The limma package of R (http://www.bioconductor.org/) was used for background correction, normalization between arrays, and differential expression analysis between 113 MM and seven control samples. Samples with an adjusted false discovery rate *p* < 0.05 and |log fold change (FC)| > 1 were considered as the threshold points for DEGs.

### Candidate Diagnostic Biomarker Screening

To identify significant prognostic variables, two machine-learning algorithms were used to predict disease status. The least absolute shrinkage and selection operator (LASSO) is a regression analysis algorithm that uses regularization to improve the prediction accuracy. The LASSO regression algorithm was carried out using the “glmnet” package in R to identify the genes significantly associated with the discrimination of melanoma and normal samples. Support vector machine (SVM) is a supervised machine-learning technique widely utilized for both classification and regression. To avoid overfitting, an RFE algorithm was employed to select the optimal genes from the meta-data cohort. Therefore, to identify the set of genes with the highest discriminative power, support vector machine recursive feature elimination (SVM-RFE) was carried out using the “e1071” package, “kernlab” package, and “caret” package in R to select the appropriate features. The overlapping genes between the two algorithms were included and the expression levels of candidate genes were further validated in the GSE46517 dataset.

### Diagnostic Value of Feature Biomarkers in Melanoma

To test the predictive value of the identified biomarkers, we generated an ROC curve using the mRNA expression data from 113 melanoma and seven control samples. The area under the ROC curve (AUC) value was utilized to determine the diagnostic effectiveness in discriminating MM from control samples and further validated in the GSE46517 dataset.

### Expression and Survival Analysis of LTBP4

TCGAportal (http://www.tcgaportal.org) and GEPIA (http://gepia.cancer-pku.cn/) were used to investigate survival probability and LTBP4 expression in tumor tissues and corresponding para-carcinoma tissues.

### Immunofluorescent Staining

Cells on the coverslips were fixed with 4% paraformaldehyde and incubated with the primary antibody against YAP (diluted 1:100; CST, 14074) at 4°C overnight. After washing with PBS, cells were then incubated with fluorescence-conjugated secondary antibody (Invitrogen, Carlsbad, CA), and subsequently, the coverslips were treated with 4,6-diamidino-2-phenylindole (DAPI; Life Technology) for 5 min for nuclear staining and then mounted on glass slides. Images were acquired using a fluorescence microscope (Olympus).

### Cells and Surgical Specimens

Five melanoma cell lines used in this study. A101D, SK-MEL-1, VMM5A, A375, and MeWo cells and control cells (human epidermal melanocytes: PEM cell) were purchased from the Cell Culture Collection of the Chinese Academy of Sciences (Beijing, China) and cultured in DMEM medium containing 10% fetal bovine serum (HyClone, Logan, UT, United States), 100 mg/ml streptomycin, and 100 U/mL penicillin in a humidified incubator containing 5% CO_2_ at 37°C for 2–3 days. Melanoma tumor tissues and corresponding normal tissues were collected from 76 patients with melanoma between October 2018 and October 2019, following surgical resection at Tianjin Medical University Eye Hospital. All patients have not received neoadjuvant treatment before surgery. All experiments were approved by the Medical Ethics Committee of Tianjin Medical University Eye Hospital and written informed consent documents were signed by all of the patients. Table 1 lists the clinical characteristics of the enrolled patients.

**TABLE 1:** Correlation between LTBP4 expression and clinicopathological parameters of patients with melanoma.

Parameters	Number of patients		*p*-value	χ^2^
	Low LTBP4 expression (n)	High LTBP4 expression (n)		
Age (years)				
<60	25	15	0.901	0.015
≥60	22	14		
Sex				
Male	28	14	0.336	0.926
Female	19	15		
Invasion				
T0-T2	14	20	0.001*	11.135
T3-T4	33	9		
TNM stage				
I-II	12	20	0.000*	13.879
III-IV	35	9		
Distal metastasis				
Yes	37	8	0.000*	19.418
No	10	21		
Lymph node metastasis				
Yes	32	9	0.002*	9.909
No	15	20		

TNM stage, Tumor-Lymph Node-Metastasis stage; LTBP4, Latent transforming growth factor beta binding protein 4; ^*^ *p* < 0.05 was considered as significant.

### LTBP4 Gene Silencing and OE and Cell Transfection

Two different shRNAs (shRNA-1 and shRNA-2; purchased from GeneChem, Shanghai, China) were used to target the LTBP4 gene KD. A nonsilencing shRNA (NC) was used as control (GeneChem). LTBP4 expressing plasmids (constructed using a pcDNA 3.1 vector) (Invitrogen; Thermo Fisher Scientific, Inc., Waltham, MA, United States) to induce LTBP4 OE. Cells were transfected with 10 nM of shRNAs or pcDNA3.1 when cells reached about 50% confluence. After 8 h of transfection using Lipofectamine-2000 (Life Technologies) according to the manufacturer’s protocol, cells were returned to normal medium in the incubator. When cells reached 85% confluence, cell supernatant, protein, and RNA were collected for later experiments.

A101D cells transfected with pcDNA3.1-LTBP4 were treated with 0, 5 ng/ml TGF-β1 (Zhang et al., 2017) (biolab Science and Technology Ltd., Beijing, China) for 48 h. Then, protein and mRNA were collected for later experiments.

### Luciferase Reporter Assay

A luciferase reporter assay was performed to detect YAP transcriptional activity in A101D and A375 cells exposed to pcDNA3.1-LTBP4 transfection, or shRNA-1-LTBP4 transfection. Briefly, the 8xGTIIC-luciferase plasmid, which contains a YAP -responsive synthetic promoter driving luciferase expression, was co-transfected into cells with a β-gal plasmid (Ambion, United States) using LipofectamineTM 2000. After 72 h, luciferase activity was examined. β-Gal activity was used as a normalization control for luciferase activity.

### YAP1 Gene or MST1 Genes OE and Cell Transfection

YAP1 expressing plasmids or MST1 expressing plasmids (constructed using a pcDNA 3.1 vector) (Invitrogen; Thermo Fisher Scientific, Inc.) to induce YAP1 OE or MST1 OE. SK-MEL-1 and A375 cells were transfected with 10 nM of pcDNA3.1 when cells reached 30–50% confluence using Lipofectamine-2000 (Life Technologies) according to the manufacturer’s protocol. After transfection for 48 h, the transfection efficiency was determined using RT-PCR assay.

### Rescue Experiment

This experiment was divided into the following four groups:1) SK-MEL-1 or A375 cells without any treatment was viewed as CTRL group; 2) SK-MEL-1 or A375 cells transfected with LTBP4 OE was viewed as LTBP4-OE group; 3) SK-MEL-1 or A375 cells co-transfected with LTBP4 OE and YAP1 OE was viewed as LTBP4-OE + YAP1-OE group; 4) SK-MEL-1 or A375 cells co-transfected with LTBP4 OE and MST1 OE was viewed as LTBP4-OE + MST1-OE group. Cell proliferation, apoptosis, migration and invasion in above groups were measured using CCK-8, colony formation, flow cytometry, transwell and wound healing assays.

### Cell Viability Assay

Cell viability was detected using CCK-8 kit instructions (Beyotime, Shanghai, China). Logarithmically growing cells were picked out, digested with 0.25% trypsin (Gibco). Transfected cells were seeded at a density of 5 × 10^3^ cells/well in 96-well plates and cultured in a 5% CO_2_ at 37°C incubators for 2 h to adhere to cells. After cells being cultured for 0, 6, 12, 24, 48 and 72 h, CCK-8 reagent was added (10 μL/well) and incubated for 4 h. The optical density was determined at 450 nm by the microplate reader. The dual-wavelength microplate reader (Beckman Coulter, United States) was used to measure the detection wavelength of 450 nm.

### Colony Formation

For the colony formation assay, 600 cells per group were plated in triplicates in a six-well plate. Dishes were taken out when the cell colonies in each well were more than 25. The numbers of cell clones were counted following staining with 1% crystal violet (Beyotime) for 5 min at room temperature, and images were captured by an optical microscope (Olympus, CX23).

### Flow Cytometry Analysis

For apoptosis measurements, the percentages of apoptotic cells were determined by flow cytometry using the Annexin V-FITC/PI cell apoptosis detection kit (Promega) according to the manufacturer’s instructions. The Q2 and Q3 were identified as the apoptotic quadrant.

### Wound-Healing Assay

Cells (4.5 × 10^5^/ml) were seeded on a six-well plate to form a confluent monolayer in a 10% FBS-containing medium. The monolayer cells were scratched by a plastic tip and washed with PBS to remove cell debris; 0.5% FBS-containing F12K or RPMI1640 were then added to each well, and the scratched monolayer was incubated in a 37 °C incubator with 5% CO_2_ for 24 h. Wound closure was measured in five random fields at × 200 magnification using ImageJ software and an inverted microscope (Olympus). Percentage of wound healing was calculated as follows: migrated cell surface area/total surface area × 100, in which, migrated cell surface area = length of cell migration (mm) × 2 × length of defined areas, total surface area = beginning width × length of defined areas.

### Transwell Invasion Assays

Cells (4×10^5^/ml) were seeded onto the upper chamber of each 24-well plate (Corning, NY, United States) with serum-free medium. The pore size of upper chamber was 8.0 µm. The lower chamber was filled with 600 µL of medium with 10% FBS. After the cells were incubated for 48 h, the cells attached to the reverse phase of the membrane were fixed with 4% paraformaldehyde for 15 min and the cells on the upper chamber were removed using cotton swabs. Then the cells located on the lower surface were stained with 0.1% crystal violet for 5 min. Cells were photographed at least five fields using a light microscope (Olympus).

### Total and Active TGFβ1 Detection

1) After determining the number of cells, the plates were washed 4 times and treated with 50 µL assay buffer C. 2) This was followed by the addition of 50 µL of diluted standards or samples. 3) The plates were washed another four times and 100 µL of detection antibody solution was added. 3) The plates were washed four more times and 100 µL of avidin-HRP D solution was added. 4) The plates were washed five times and 100 µL of substrate solution was added. 5) Finally, 100 µL of stop solution was added to the plates. The absorbance was read at 450 nM.

### *In vivo* Tumorigenesis in Nude Mice

Animal experiments were done according to Institutional Animal Care and Use Committee (IACUC) protocol and approved by Tianjin Medical University Animal Center for Use and Care of Animals. The establishment of the subcutaneous melanoma tumor model was treated by subcutaneous injection of SK-MEL-1 with transiently transfected siRNA-LTBP4 and A101D cells with transiently transfected pcDNA 3.1-LTBP4. Four-week-old female BALB/C nude mice (n = 24; Tianjin Medical University Animal Center, Tianjin, China) were required to establish melanoma model mice for 4 weeks (Wu et al., 2020a). Tumor volumes were measured every 3 days. The tumor volume was calculated using the following formula: volume = (length × width^2^)/2. Mice were sacrificed 32 days after the injection and the size of the tumor was measured by vernier caliper and the weight of the tumor was measured by electronic balance.

### Immunohistochemistry (IHC) Staining

LTBP4 expression between melanoma tissues and adjacent noncancerous tissues in patients with melanoma was determined by IHC staining. For IHC, sections were incubated with anti-LTBP4 (1:300 dilution) antibody. LTBP4 staining was scored by two independent pathologists. LTBP4 or cleaved caspase-3 expression between LTBP4-OE group and LTBP4-KD group in tumor tissues of BALB/C nude mice was determined by IHC staining. Sections were photographed in at least five fields using a light microscope. For IHC, sections were incubated with anti-LTBP4 (1:500 dilution) antibody, anti-cleaved caspase-3 (1:200 dilution) antibody, anti-Ki67 (1:600 dilution) antibody, anti-E-cadherin (1:500 dilution) antibody, anti-YAP1 (1:800 dilution) antibody, and anti-N-cadherin (1: 500 dilution) antibody. Protein’s staining was scored by researcher. The scoring system was based on the staining intensity and extent. Staining intensity was classified as 0 (negative), 1 (weak), 2 (moderate), and 3 (strong). Staining extent depended on the percentage of positive cells and was divided into 0 (<5%), 1 (5–25%), 2 (26–50%), 3 (51–75%), and 4 (>75%). According to the staining intensity and the staining extent scores, the IHC result was classified as 0–1, negative (−); 2–4, weakly positive (+); 5–8, moderately positive (++); and 9–12, strongly positive (+++).

### Western Blotting

Protein lysates were prepared from cells using 500 µL of RIPA buffer with 1 mM phenylmethane sulfonyl fluoride. A total of 40 ug protein was separated by 10% (SDS)-polyacrylamide gel for electrophoresis and then transferred onto polyvinylidene difluoride (PVDF) membrane. The membranes were blocked with PBS containing 0.1% Tween-20 (PBST) and 5% nonfat milk (w/v) for 1 h at room temperature. After they were washed with PBST, the membranes were probed with antibodies overnight at 4°C. Antibody against LTBP4 was obtained from Shanghai Yu Bo Biotech Co.,Ltd. (Shanghai, China); antibody against cleaved caspase-3/Ki67/E-cadherin/YAP1/MST1/CTGF/Cyr61//TGFβR2 and phosph-YAP1/MST1 antibody was obtained from Abcam (Cambridge, UK); antibody against MOB1 and phospho-MOB1 were obtained from Cell Signaling Technology, Inc. (United States); antibody against Birc5 and phosphor-Birc5 were obtained from CUSABIO engineering co. Ltd. (Wuhan, Hubei, China); antibody against β-actin or LaminB were obtained from Beyotime (China). The membranes were washed again with PBST, then horseradish peroxidase-labeled IgG at 1:5,000 dilution was added at room temperature for 1 h, and the blots were developed using enhanced chemiluminescence western blotting reagents. β-actin or LaminB was used as an internal control.

### Real-Time PCR (RT-PCR)

Real-time PCR was performed on a Step Two Real-Time PCR System (Applied Biosystems) using the comparative Ct quantization method. Real-time PCR Master Mix (Toyobo) was used to detect and quantify the expression level of the target gene. β-actin and GAPDH were used as the internal control (Lin and Redies, 2012). The primers used were as follows: CTGF, 5′- AGT​GCA​TCC​GTA​CTC​CCA​AA-3' (F) and 5′- CCG​TCG​GTA​CAT​ACT​CCA​CA-3' (R); Cyr61, 5′- GCA​GCG​TTT​CCC​TTC​TAC​AG-3' (F) and 5′- ATG​AGT​CCC​ATC​ACC​CAC​AC-3' (R); Birc5, 5′- AAC​AGT​GGC​TGC​TTC​TCT​CT-3' (F) and 5′- GCC​TTC​TTC​CTC​CCT​CAC​TT-3' (R); β-actin, 5′- ACT​CTT​CCA​GCC​TTC​CTT​CC-3' (F), 5′-CAA​TGC​CAG​GGT​ACA​TGG​TG-3' (R); LTBP4, 5′- CGA​CAT​GCC​AGA​CTT​TGA​GG-3' (F) and 5′- ACC​AGC​ATA​GCT​TCC​ACC​TT-3' (R); TGFβR2, 5′-CCC​CAG​GTA​AGG​ATA​GCA​G-3' (F) and 5′-CCA​GGT​AGG​CAG​TGG​AAA-3' (R); GAPDH, 5′-CCT​TCC​GTG​TCC​CCA​CT-3' (F) and 5′-GCC​TGC​TTC​ACC​ACC​TTC-3' (R).

### Statistical Analysis

Statistical analyses were performed using SPSS 19.0 (IBM, Armonk, NY, United States). Correlations between LTBP4 expression and the clinicopathological variables were analyzed using the Pearson χ2 analysis. The average value of LTBP4 > 1.925 as high expression. Survival was analyzed using the Kaplan-Meier method, and differences were evaluated using the log-rank test. The Cox proportional hazards model was used for univariate analysis to examine the potential prognostic value of different variables on OS. Data were evaluated using ANOVA with LSD test for multiple comparisons and Student’s t-test between two groups. *p* < 0.05 was considered to indicate a statistically significant difference.

## Results

### LTBP4 Is a Feature Biomarker for Melanoma

**FIGURE 1:**
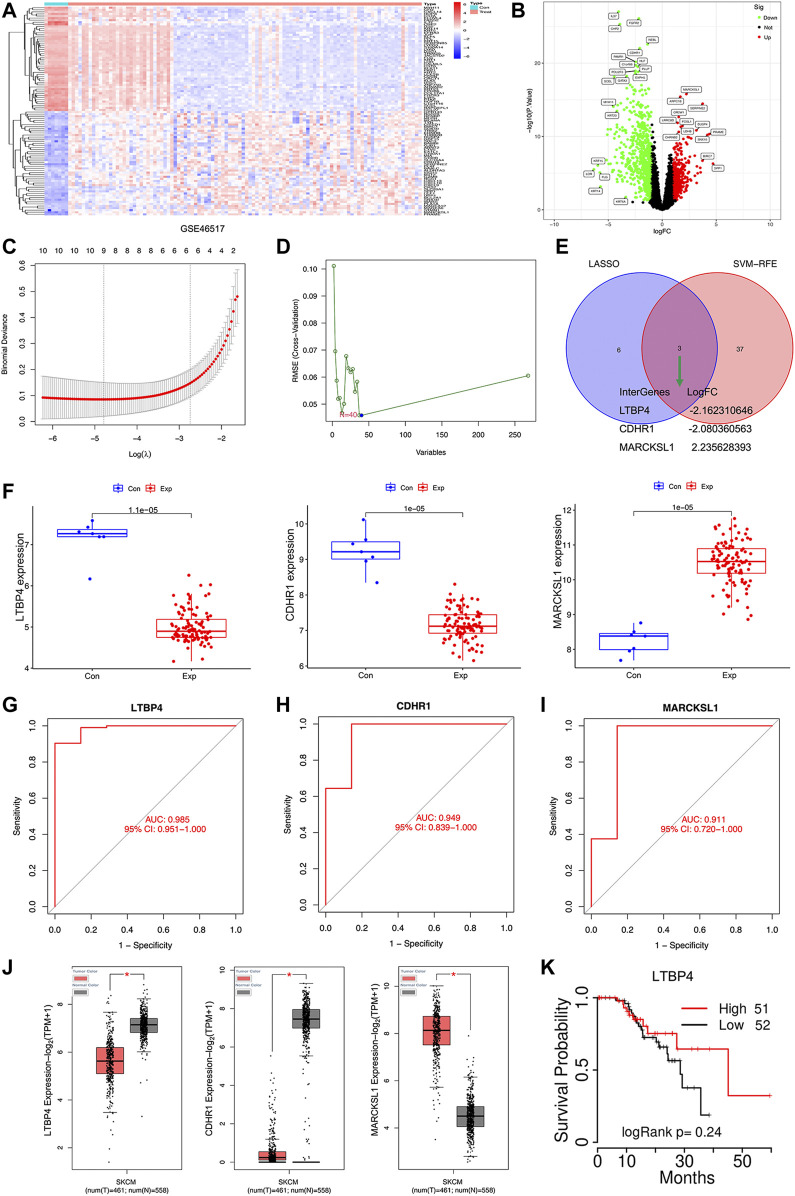
Bioinformatics analysis of DEGs. The heatmap plot of DEGs **(A)** and Venn plot of DEGs **(B)** in the GSE46517 dataset. **(C)** Tuning feature selection in the least absolute shrinkage and selection operator model. **(D)** A plot of biomarkers selection via support vector machine-recursive feature elimination (SVM-RFE) algorithm. **(E)** Venn diagram demonstrating four diagnostic markers shared by the least absolute shrinkage and selection operator and SVM-RFE algorithms. **(F)** Validation of the expression of diagnostic biomarkers (LTBP4, CDHR1, and MARCKSL1) in the GSE46517 dataset. The receiver operating characteristic (ROC) curve of the diagnostic effectiveness of LTBP4 **(G)**, CDHR1 **(H)**, and MARCKSL1 **(I)** **(J)** LTBP4 expression was determined via using the GEPIA database **(K)** The survival probability of LTBP4 was determined via using the TCGAportal database.

There were 1,155 DEGs (including 738 down-regulated and 417 up-regulated) between normal tissue samples and melanoma samples in the GSE46517 dataset (Figures 1A,B). Two different algorithms were used to screen potential biomarkers. Thereafter, the number of DEGs was narrowed down using the LASSO regression algorithm, and nine variables were identified as the diagnostic biomarkers for melanoma (Figure 1C). A subset of 40 features among the DEGs was determined using the SVM-RFE algorithm (Figure 1D). Ultimately, the three overlapping features (LTBP4, CDHR1, and MARCKSL1) between these two algorithms were selected (Figure 1E). Furthermore, to generate the more accurate and reliable results, the GSE46517 dataset was utilized to verify the expression levels of the three features. The expression levels of LTBP4 and CDHR1 in melanoma tissues were notably lower than those in the control group, while MARCKSL1 expression in melanoma tissues was notably higher than that in the control group (Figure 1F). Therefore, the three identified genes were incorporated to establish a diagnostic model using the logistic regression algorithm in the metadata cohort. Next, the diagnostic ability of these three biomarkers in discriminating melanoma from the control samples demonstrated favorable diagnostic value, with the AUC values of 0.985 (95% CI 0.951–1.000) in LTB4 (Figure 1G), 0.949 (95% CI 0.839–1.000) in CDHR1 (Figure 1H), and 0.911 (95% CI 0.720–1.000) in MARCKSL1 (Figure 1I). Moreover, more powerful discrimination ability was confirmed in the GSE60993 dataset, with the AUC value of 0.985 (95% CI 0.951–1.000) in LTB4, indicating that these feature biomarkers achieved high diagnostic ability. Then, the GEPIA database similarly showed that LTBP4 was significantly down-regulated in melanoma tissues (Figure 1J), and the TCGAportal database demonstrated no significant difference in the ability of LTBP4 to predict survival probability between its high expression group and low expression group (Figure 1K). But when the survival time exceeded 15 months, the survival rate of patients with low LTBP4 expression was lower than that of patients with high LTBP4 expression.

### Down-Regulation of LTBP4 Is Closely Related to the Poor Survival of Patients With Melanoma

**FIGURE 2:**
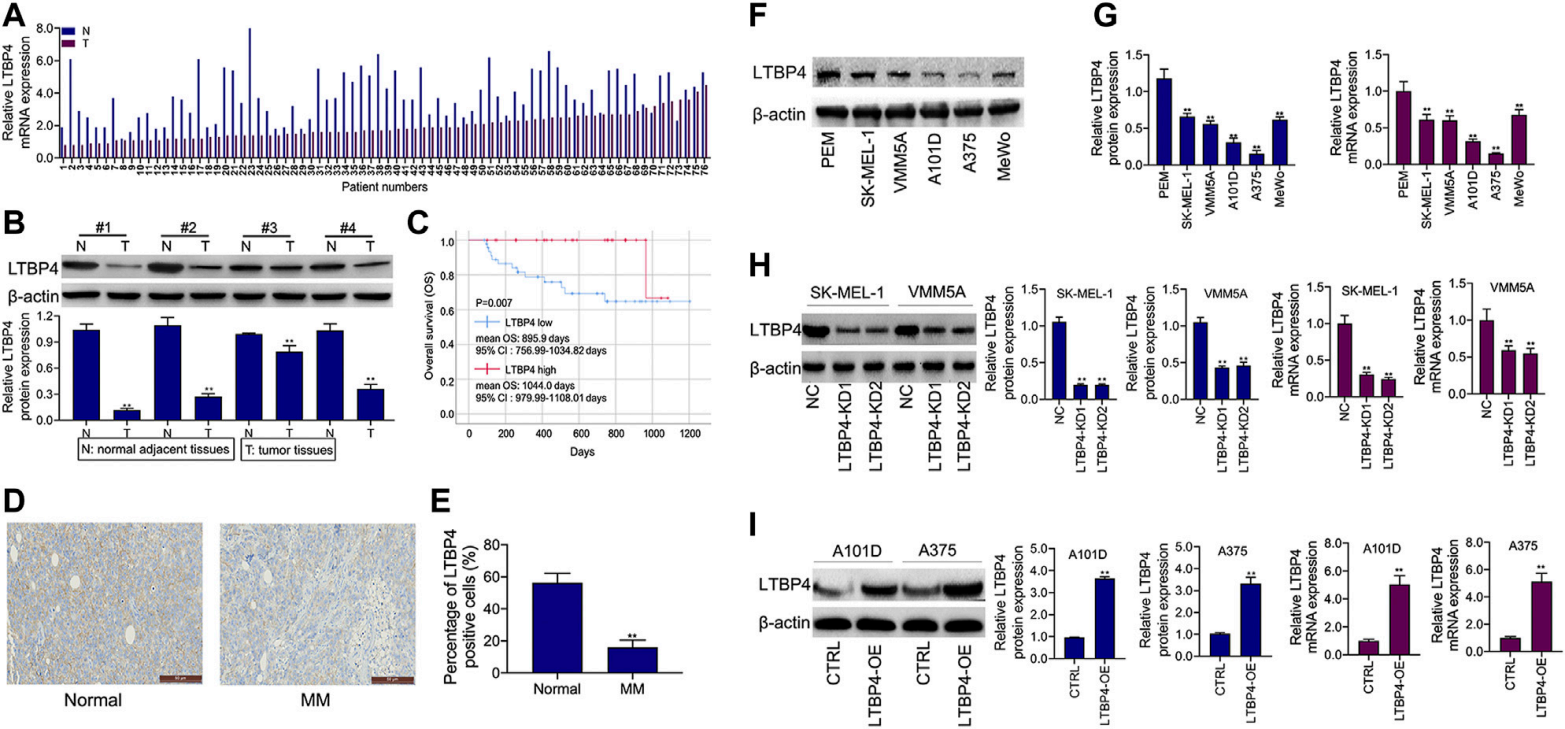
LTBP4 expression was down-regulation in melanoma tissues and cell lines, which was closely related to the poor survival for patients with melanoma. **(A)** The mRNA level of LTBP4 in melanoma tissues was detected by RT-PCR assay. **(B)** The protein level of LTBP4 in melanoma tissues was detected by western blotting assay. **(C)** Survival curve was performed by using the Kaplan-Meier method, and differences between the curves in LTBP4 high expression group and LTBP4 low expression group were determined by log‐rank test **(D** and **E)** LTBP4 expression in melanoma tissues was determined by IHC staining. **(F)** The protein level of LTBP4 in melanoma cell lines was detected by western blotting assay. **(G)** The mRNA level of LTBP4 in melanoma cell lines was detected by RT-PCR assay. **(H)** shRNA-LTBP4#1 or #2 was transfected into SK-MEL-1 and VMM5A cells, the protein and mRNA levels of LTBP4 were detected by western blotting and RT-PCR assays. **(I)** LTBP4 expressing plasmids were transfected into A101D and A375 cells, the protein and mRNA levels of LTBP4 were detected by western blotting and RT-PCR assays. β-actin was used as a load control. Data are presented as the mean ± standard deviation. **p < 0.01 vs NC/normal/HaCaT group.

As shown by the results of RT-PCR and Western blotting assays, LTBP4 was significantly down-regulated in cancer tissues from 76 patients with melanoma, which was associated with invasion, TNM stage, distal metastasis, and lymph node metastasis (Figures 2A,B and Table 1). We divided the 76 melanoma patients into high and low LTBP4 expression groups according to the average LTBP4 expression level (average = 1.925). Kaplan‐Meier curve was performed to estimate survival, and the log‐rank test was used to compare the curves. The OS of high LTBP4 expression group was longer (mean OS: 1,044.0 days, 95% CI: 979.99–1,108.01 days) than that of low LTBP4 expression group (mean OS: 895.9 days, 95% CI: 756.99–1,034.82 days) (*p* = 0.007) (Figure 2C). Univariate Cox regression analysis revealed that low LTBP4 expression [hazard ratio (HR), 0.062; 95% confidence interval (CI), 0.006–0.694; *p* = 0.024] was associated with patient survival (Table 2). While invasion, TNM stage, distal metastasis, lymph node metastasis, age, and sex were not associated with survival (all *p* > 0.05). Likewise, the LTBP4 protein expression was significantly downregulated in melanoma tissues, as revealed by IHC staining (Figures 2D,E). Subsequently, we found that LTBP4 expression was significantly down-regulated in melanoma cell lines, as evidenced by RT-PCR and Western blotting assays (Figures 2F,G). LTBP4 expression in SK-MEL-1 and VMM5A cells was significantly higher than that in A101D and A375 cells. shRNA-LTBP4 # one or # two was transfected into SK-MEL-1 and VMM5A cells to inhibit LTBP4 expression, whereas the LTBP4 expressing plasmids were transfected into A101D and A375 cells to over-express LTBP4 expression (Figures 2H,I).

**TABLE 2:** Univariate Cox proportional hazards analyses of LTBP4 expression and overall survival for patients with melanoma.

Parameters	Univariate analysis	
	HR (95% CI)	*p-*value
Invasion		
T0-T2 vs T3-T3	0.799 (0.228–2.797)	0.726
TNM stage		
I-II vs III-IV	0.650 (0.165–2.556)	0.537
Distal metastasis		
Yes vs No	1.278 (0.342–4.781)	0.715
Lymph node metastasis		
Yes vs No	1.176 (0.365–3.790)	0.786
Age		
<60 vs ≥ 60	1.406 (0.434–4.550)	0.570
Sex		
Male vs Female	1.013 (0.303–3.390)	0.983
LTBP4 expression		
Low vs High	0.062 (0.006–0.694)	0.024*

TNM stage, Tumor-Lymph Node-Metastasis stage; LTBP4, Latent transforming growth factor beta binding protein 4; HR, hazard ratio; CI, confidence interval. *p < 0.05 was considered as significant.

### LTBP4 Regulates the Proliferation and Apoptosis of Melanoma Cell Lines

**FIGURE 3:**
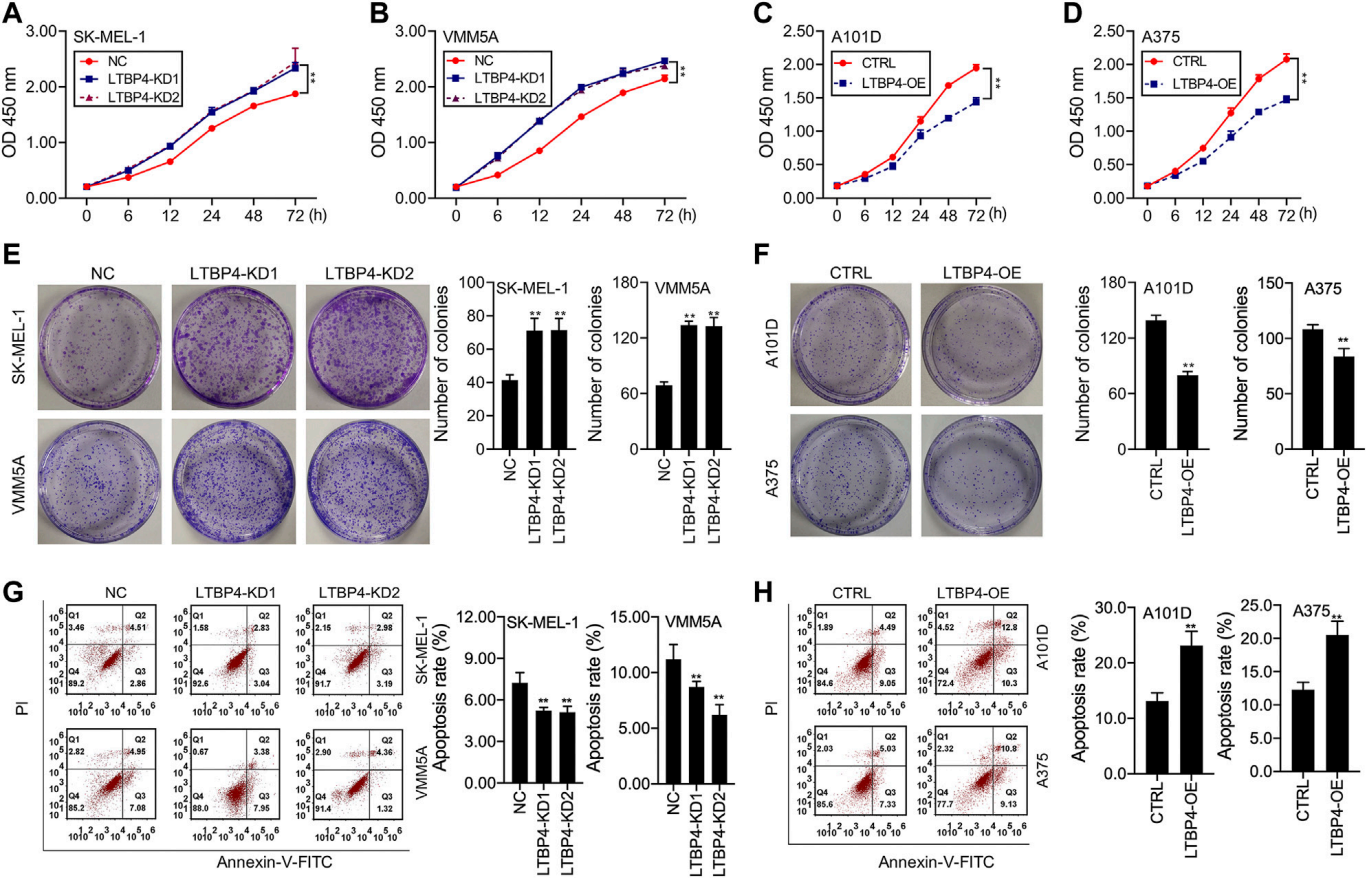
LTBP4 significantly regulated the proliferation and apoptosis in melanoma cell lines **(A** and **B)** The viability of shRNA-LTBP4#1 or #2 transfected SK-MEL-1 and VMM5A cells was detected by CCK-8 assay **(C** and **D)** The viability of LTBP4 expressing plasmids transfected A101D and A375 cells was detected by CCK-8 assay. **(E)** The proliferation of shRNA-LTBP4#1 or #2 transfected SK-MEL-1 and VMM5A cells was detected by colony formation assay. **(F)** The proliferation of LTBP4 expressing plasmids transfected A101D and A375 cells was detected by colony formation assay. **(G)** The apoptosis level of shRNA-LTBP4#1 or #2 transfected SK-MEL-1 and VMM5A cells was detected by flow cytometry assay. **(H)** The apoptosis level of LTBP4 expressing plasmids transfected A101D and A375 cells by flow cytometry assay. Data are presented as the mean ± standard deviation. **p < 0.01 vs NC/CTRL group.

Our results showed that LTBP4 knockdown promoted the viability of SK-MEL-1 and VMM5A cells (Figures 3A,B), whereas LTBP4 overexpression inhibited the viability of A101D and A375 cells (Figures 3C,D). Besides, CCK8 analysis showed that the doubling time of SK-MEL-1 cells was 12 h (Figure 3A), while that of VMM5A cells was 8 h (Figure 3B), that of A101D cells was 18 h (Figure 3C), and that of A375 cells was 16 h (Figure 3D). Unsurprisingly, the results of clone formation assay indicated that LTBP4 knockdown promoted the proliferation of SK-MEL-1 and VMM5A cells (Figure 3E), while LTBP4 overexpression inhibited the proliferation of A101D and A375 cells (Figure 3F). Flow cytometry results demonstrated that the apoptosis of SK-MEL-1 and VMM5A cells was significantly suppressed by LTBP4-KD1 or LTBP4-KD2; meanwhile, the apoptosis of A101D and A375 cells was significantly promoted by LTBP4 OE (Figures 3G,H). However, there was no significant difference in cell viability, proliferation or apoptosis between LTBP4-KD1 group and LTBP4-KD2 group.

### LTBP4 Significantly Regulates the Invasion and Migration of Melanoma Cell Lines

**FIGURE 4:**
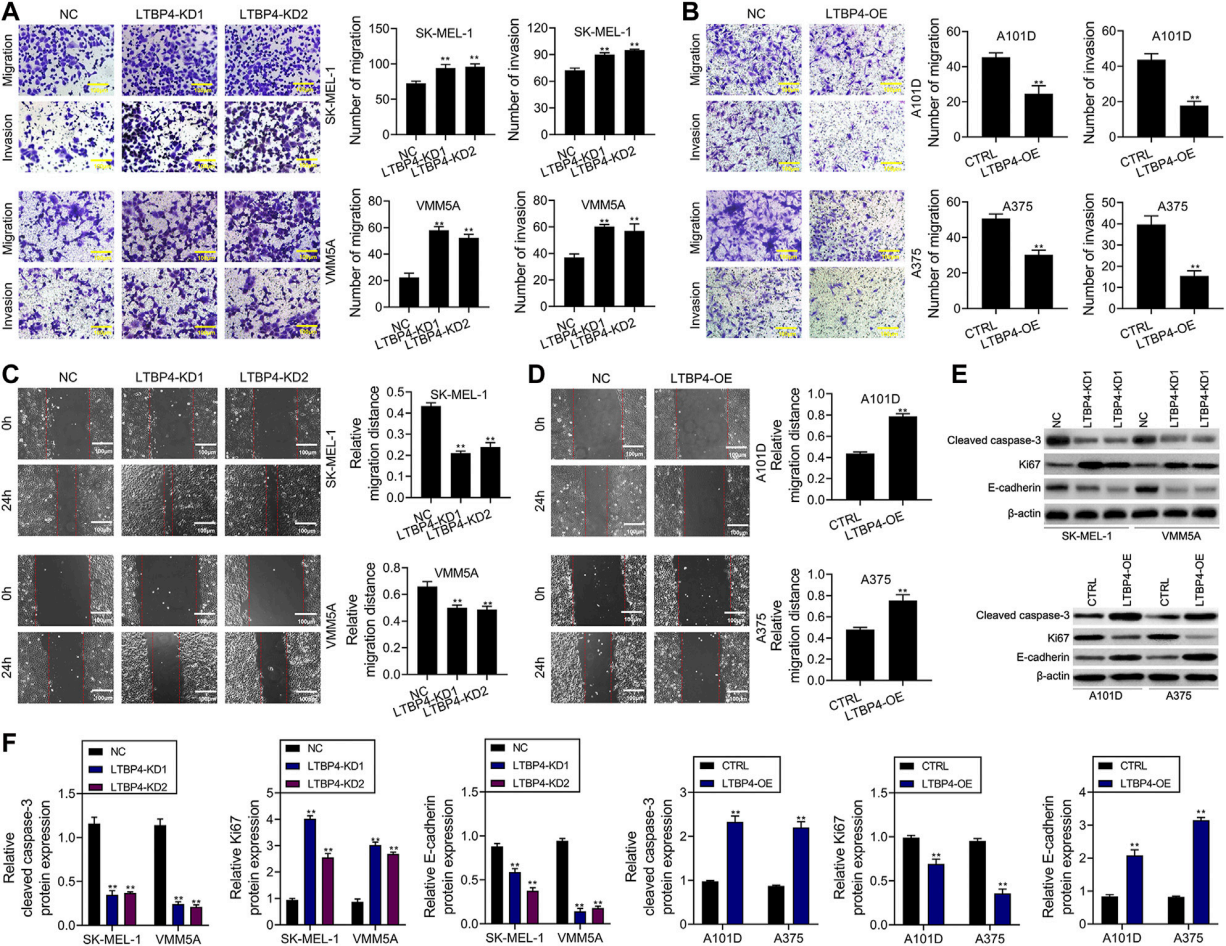
LTBP4 significantly regulated the invasion and migration in melanoma cell lines, and closely related to the expressions of cleaved caspase-3, Ki67 and E-cadherin. **(A)** The migration and invasion of shRNA-LTBP4#1 or #2 transfected SK-MEL-1 and VMM5A cells was detected by transwell assay. **(B)** The migration and invasion of LTBP4 expressing plasmids transfected A101D and A375 cells was detected by transwell assay. **(C)** The migration of shRNA-LTBP4#1 or #2 transfected SK-MEL-1 and VMM5A cells was detected by transwell assay. **(D)** The migration of LTBP4 expressing plasmids transfected A101D and A375 cells was detected by wound healing assay **(E** and **F)** The protein levels of cleaved caspase-3, Ki67, and E-cadherin in shRNA-LTBP4#1 or #2 transfected SK-MEL-1 and VMM5A cells or LTBP4 expressing plasmids transfected A101D and A375 cells were detected by western blotting assay. β-actin was used as a load control. Data are presented as the mean ± standard deviation. **p < 0.01 vs NC/CTRL group.

According to results of Transwell assay, LTBP4 silencing significantly promoted the invasion of SK-MEL-1 and VMM5A cells (Figure 4A), whereas LTBP4 overexpression markedly restrained the invasion of A101D and A375 cells (Figure 4B). Conversely, wound healing assay suggested that LTBP4 silencing significantly promoted the migration of SK-MEL-1 and VMM5A cells (Figure 4C), while LTBP4 over-expression markedly restrained the migration of A101D and A375 cells (Figure 4D). Next, changes in the expression of cleaved caspase-3, Ki67, and E-cadherin proteins within melanoma cell lines with LTBP4 knockdown or overexpression were determined by Western blotting. The results showed that LTBP4 silencing significantly inhibited the expression levels of cleaved caspase-3 and E-cadherin, and increased Ki67 expression in SK-MEL-1 and VMM5A cells. Meanwhile, LTBP4 overexpression significantly increased the expression levels of cleaved caspase-3 and E-cadherin, but suppressed Ki67 expression in A101D and A375 cells (Figures 4E,F).

### LTBP4 Affects the Oncogenicity of Melanoma Cells *in vivo*

**FIGURE 5:**
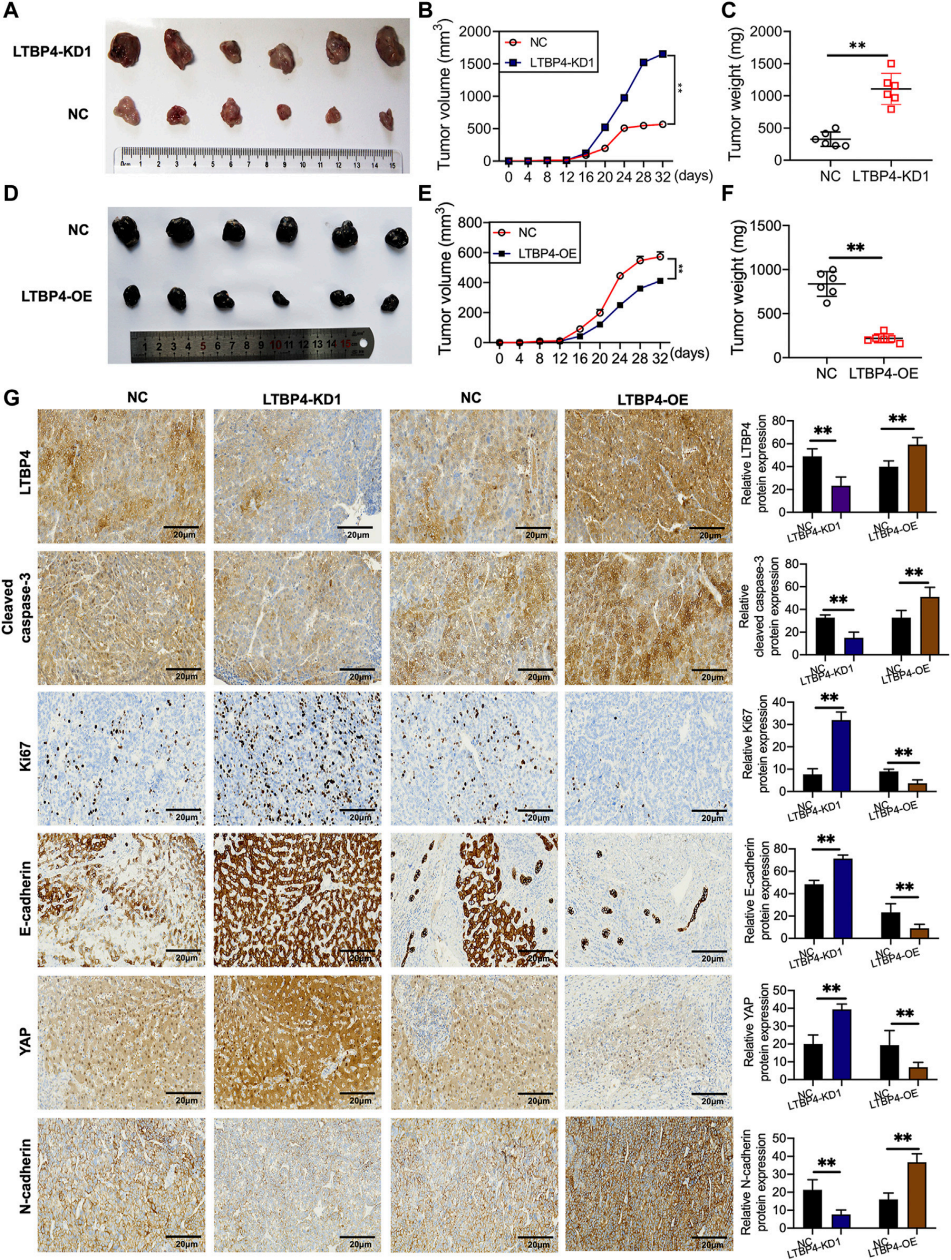
LTBP4 affected the oncogenicity of melanoma cell *in vivo*. SK-MEL-1 cells transfected with shRNA-LTBP4, or NC were subcutaneously injected into the nude mice, **(A)** The nude mice were sacrificed, and the tumors were collected after 32 days; **(B)** the volume of the tumors were determined; **(C)** the weight of the tumors were determined; A101D cells transfected with LTBP4 overexpression or NC were subcutaneously injected into the nude mice, **(D)** The nude mice were sacrificed, and the tumors were collected after 30 days; **(E)** the volume of the tumors were determined; **(F)** the weight of the tumors were determined; **(G)** the expression of Ki67, cleaved caspase-3, Ki67, E-cadherin, YAP, and N-cadherin in the tumors collected from different groups were determined using IHC staining. Data are presented as the mean ± standard deviation. **p < 0.01 vs NC group.

shRNA-LTBP4 was first of all transfected into SK-MEL-1 cells, then the transfected cells were injected into the nude mice subcutaneously, and the tumor weight and volume were measured using the electronic balance and vernier caliper, respectively (Figure 5A). As a result, LTBP4 knockdown promoted tumor growth (Figures 5B,C), whereas LTBP4 overexpression inhibited tumor growth (Figures 5D–F). Next, IHC staining results showed that the expression of LTBP4, cleaved caspase-3, and N-cadherin was inhibited by LTBP4 knockdown, while that of Ki67, E-cadherin, and YAP1 was promoted by LTBP4 knockdown (Figure 5G). Unsurprisingly, overexpression of LTBP4 increased the expression of LTBP4, cleaved caspase-3, and N-cadherin, but inhibited that of Ki67, E-cadherin, and YAP1 in tumor tissues of mice.

### LTBP4 Inhibited the Transcriptional Activity of YAP1 by Promoting YAP1 Nuclear-Cytoplasmic Translocation and Its Phosphorylation

**FIGURE 6:**
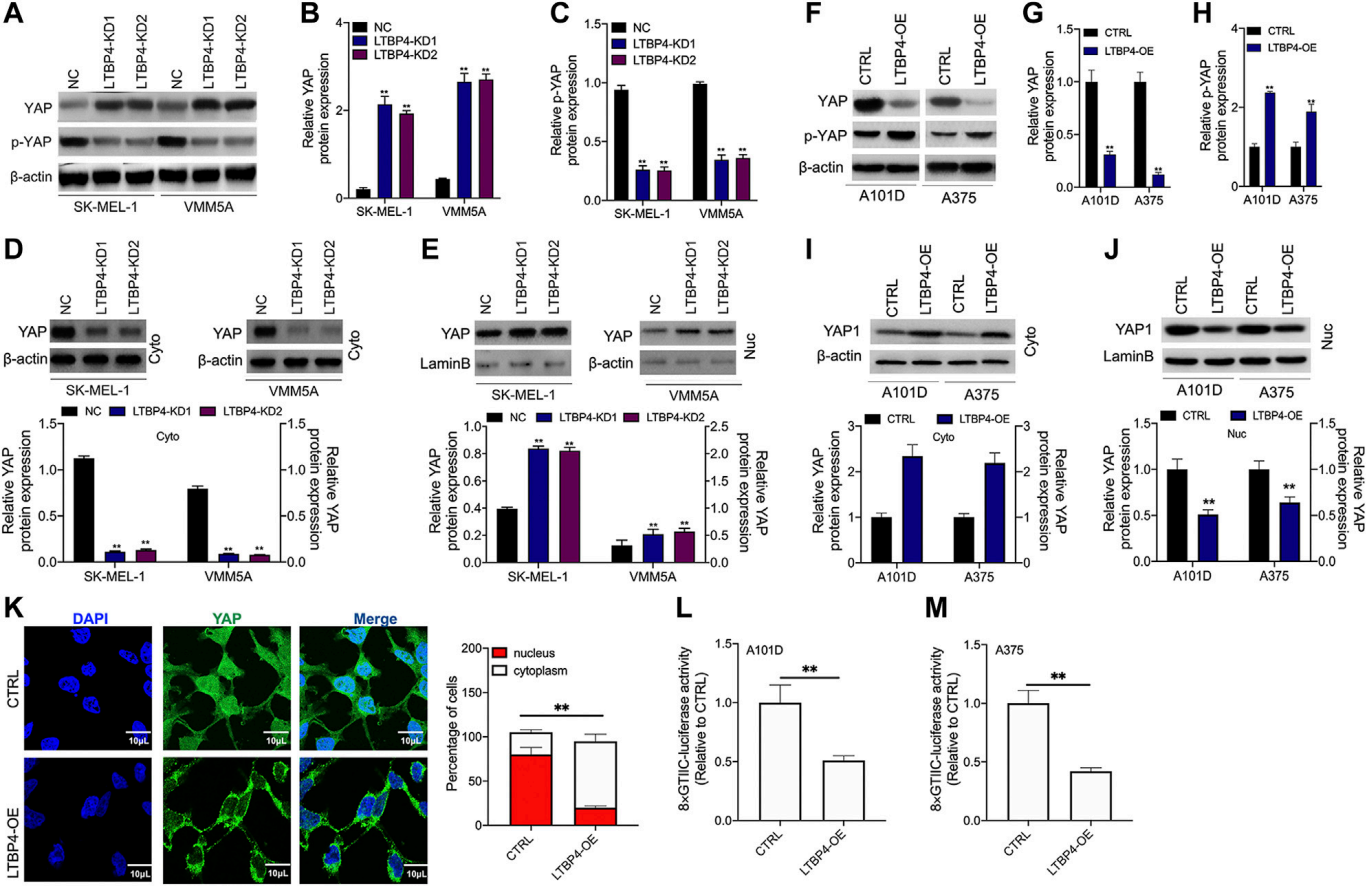
LTBP4 inhibited the transcriptional activity of YAP1 by promoting YAP1 nuclear-cytoplasmic translocation and its phosphorylation. After shRNA-LTBP4#1 or #2 was transfected into SK-MEL-1 and VMM5A cells, the expressions of MST1 and MOB1 **(A**,**B)**, the phosphorylation level of MST1 and MOB1 **(C)**, and cytoplasmic YAP1 expression **(D)** and nuclear YAP1 expression **(E)** were detected by western blotting. After LTBP4 expressing plasmids transfected A101 and A375 cells, the expressions of MST1 and MOB1 **(F**,**G)**, the phosphorylation level of MST1 and MOB1 **(H)**, and cytoplasmic YAP1 expression **(I)** and nuclear YAP1 expression **(J)** were detected by western blotting **(K)** The nuclear-cytoplasmic translocation of YAP1 in A101D cells transfected with LTBP4-overexpression vector was examined by immunofluorescence assay **(L** and **M)** The luciferase activity of 8xGTIIC-luciferase, a YAP-responsive synthetic promoter driving luciferase expression plasmid, was evaluated in A101D and A375 cells following LTBP4-overexpression vector transfection or no transfection. β-actin and LaminB were used as a load control. Data are presented as the mean ± standard deviation. **p < 0.01 vs CTRL group.

YAP1 has been shown to contribute to melanoma progression, therefore, we wondered whether LTBP4 affected YAP1 activity. Our Western blotting results showed that silencing of LTBP4 up-regulated YAP1 expression and suppressed the phosphorylation level of YAP1 in SK-MEL-1 and VMM5A cells (Figures 6A–C). Furthermore, YAP1 was lowly expressed in the cytoplasm of SK-MEL-1 and VMM5A cells transfected with shRNA-LTBP4 (Figure 6D). At the same time, YAP1 was highly expressed in the nuclei of SK-MEL-1 and VMM5A cells transfected with shRNA-LTBP4 (Figure 6E). Afterwards, LTBP4 overexpression was found to suppress YAP1 expression and enhance the phosphorylation of YAP1 in A101D and A375 cells (Figures 6F–H). Differently, LTBP4 overexpression increased the cytoplasmic YAP1 expression and inhibited its nuclear expression (Figures 6I,J). Consistently, the nuclear-cytoplasmic translocation of YAP1 was promoted by LTBP4 overexpression (Figure 6K). Furthermore, we conducted luciferase reporter assay, which showed that LTBP4 overexpression decreased the YAP1 transcriptional activity, which was characterized by the decreased activity of 8xGTIIC luciferase, a YAP-responsive synthetic promoter that drove luciferase expression (Fig. 6L-m).

### LTBP4 Inhibits the Activation of the Hippo Signaling Pathway *in vitro*

**FIGURE 7:**
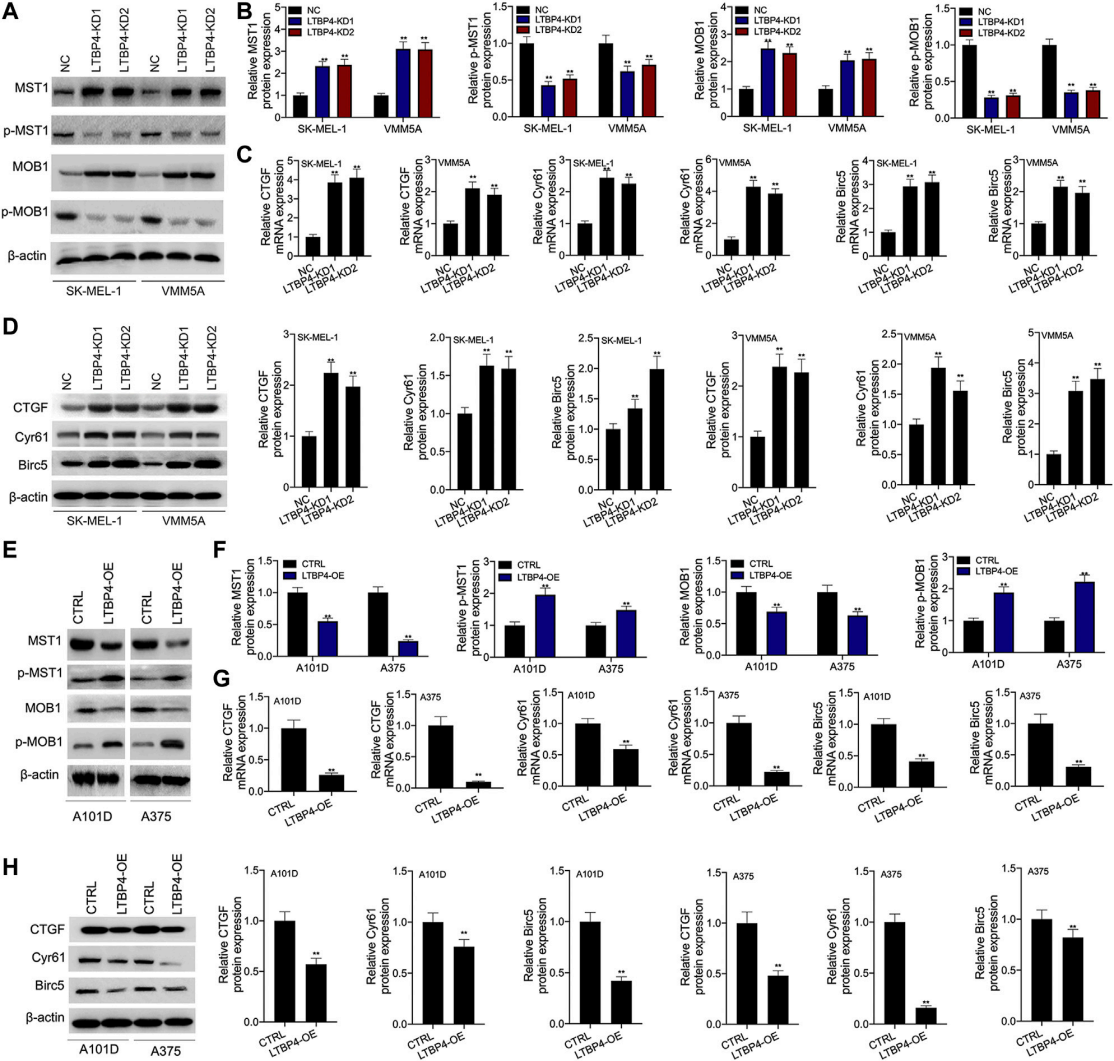
LTBP4 inhibited the activation of Hippo signaling pathway *in vitro*. After shRNA-LTBP4#1 or #2 was transfected into SK-MEL-1 and VMM5A cells **(A** and **B)** the expressions of MST1 and MOB1 and the phosphorylation level of MST1 and MOB1 were detected by western blotting assay, **(C)** the mRNA levels of CTGF, Cyr61, and Birc5 were detected by RT-PCR assay, **(D)** the protein levels of CTGF, Cyr61, and Birc5 were detected by western blotting assay. After LTBP4 expressing plasmids transfected A101 and A375 cells **(E** and **F)** the expressions of MST1 and MOB1 and the phosphorylation level of MST1 and MOB1 were detected by western blotting assay, **(G)** the mRNA levels of CTGF, Cyr61, and Birc5 were detected by RT-PCR assay, and **(H)** the protein levels of CTGF, Cyr61, and Birc5 were detected by western blotting assay. β-actin was used as a load control. Data are presented as the mean ± standard deviation. **p < 0.01 vs CTRL/or NC group.

Our Western blotting results indicated that the silencing of LTBP4 up-regulated the expression of MST1 and MOB1, but suppressed the phosphorylation levels of MST1 and MOB1 in SK-MEL-1 and VMM5A cells (Figures 7A,B). Next, the mRNA and protein levels of CTGF, Cyr61, and Birc5 in SK-MEL-1 and VMM5A cells transfected with shRNA-LTBP4 were significantly up-regulated, as evidenced by RT-PCR and Western blotting assays (Figure 7D). In addition, there was no significant difference in the expression of YAP1, MST1, MOB1, CTGF, Cyr61, or Birc5 within SK-MEL-1 and VMM5A cells between LTBP4-KD1 group and LTBP4-KD2 group.

Furthermore, our results demonstrated that LTBP4 overexpression down-regulated the expression of MST1 and MOB1, but promoted the phosphorylation of MST1 and MOB1 in A101D and A375 cells (Figures 7E,F). Next, the mRNA and protein levels of CTGF, Cyr61, and Birc5 in A101D and A375 cells transfected with LTBP4 expression plasmids were significantly down-regulated, as evidenced by RT-PCR and Western blotting assays (Figures 7G,H).

### LTBP4 Suppresses the Secretion of Active TGFβ1, the Active TGFβ1-Stimulated YAP1 Nuclear-Cytoplasmic Translocation and the Hippo Signaling Pathway

**FIGURE 8:**
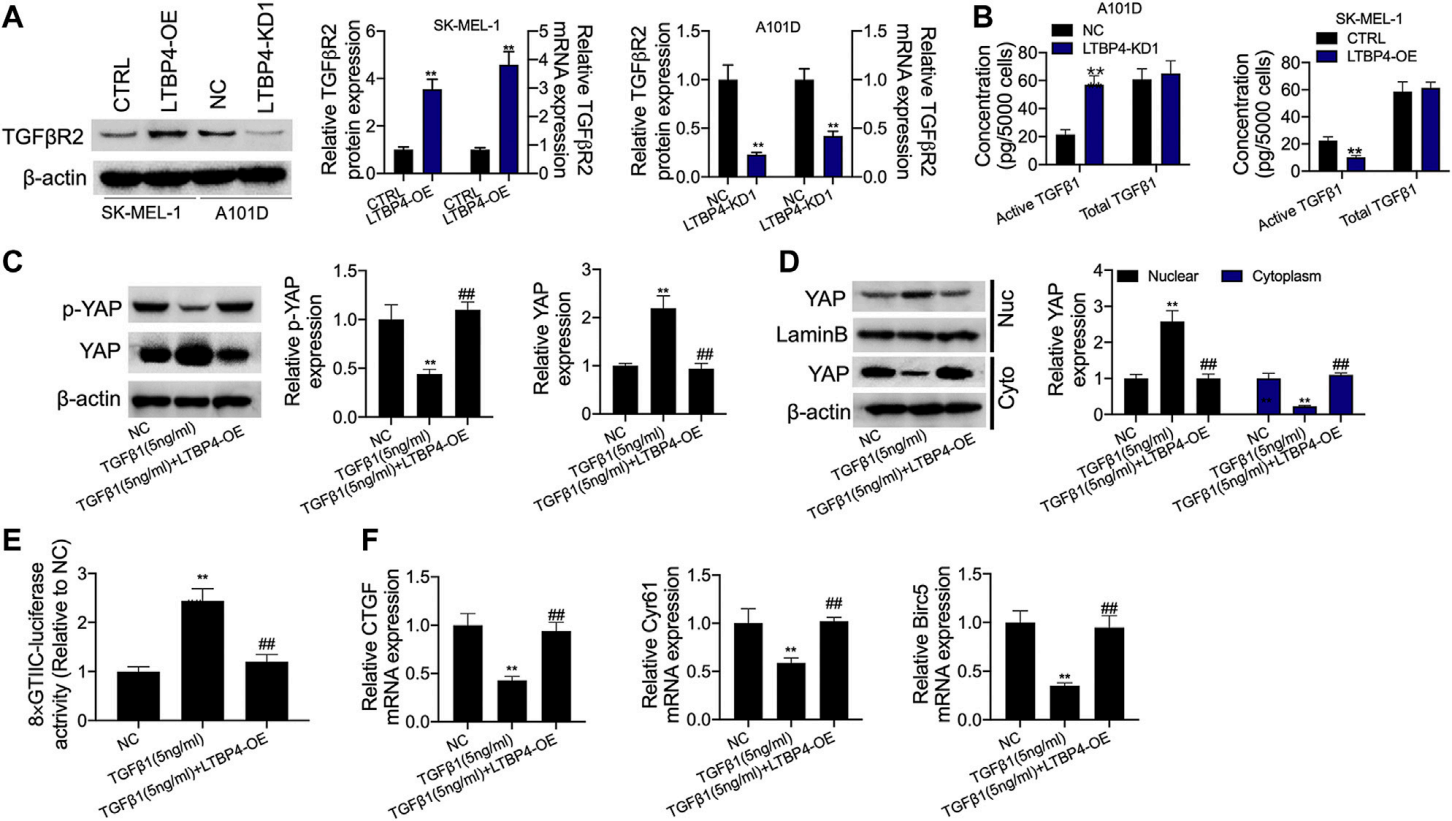
LTBP4 suppressed secretion of active TGFβ1 as well as active TGFβ1-stimulated YAP1 nuclear-cytoplasmic translocation and the Hippo signaling inhibition. **(A)** Western blot, RT-PCR and ELISA analysis of TGFβRII expression **(A)** and total and active TGFβ1 **(B)** in shRNA-LTBP4#1 transfected SK-MEL-1 cells and LTBP4 expressing plasmids transfected A101 cells. A101D cells transfected with pcDNA3.1-LTBP4 were treated with 5 ng/ml TGF-β1, then, western blot analysis of YAP phosphorylation **(C**), cytoplasmic YAP1 expression and nuclear YAP1 expression **(D)**, the luciferase activity of 8xGTIIC-luciferase analysis of YAP1 transcriptional activity **(E)**, and RT-PCR analysis of CTGF, Cyr61, and Birc5 expression **(F)**. β-actin and LaminB were used as a load control. Data are presented as the mean ± standard deviation. **p < 0.01 vs CTRL/or NC group.

According to our findings, LTBP4 overexpression promoted TGFβR2 expression, while LTBP4 knockdown inhibited TGFβR2 expression (Figure 8A). The secretion of active TGFβ1 significantly increased in LTBP4 knockdown transfected A101D cells but decreased in LTBP4 overexpression transfected SK-MEL-1 cells, and the total TGFβ1 did not significantly increase in the two states (Figure 8B). We further investigated the activation of Hippo-YAP1 signaling pathway in A101D cells treated with TGF-β1. As a result, TGF-β1 treatment inhibited YAP1 phosphorylation (Figure 8C). Consistent with the decreased phosphorylation of YAP1, TGF-β1 treatment induced YAP1 translocation from the cytoplasm to the nucleus (Figure 8D). Next, the luciferase reporter assay showed that TGFβ1 treatment increased the YAP1 transcriptional activity (Figure 8E) but inhibited the expression of CTGF, Cyr61, and Birc5 (Figure 8F). However, these effects were reversed by the transfection of LTBP4 overexpression.

### LTBP4 Overexpression Inhibits the Proliferation, Invasion, and Migration, and Promoted the Apoptosis of Melanoma Cells via the Hippo-YAP Signaling Pathway

**FIGURE 9:**
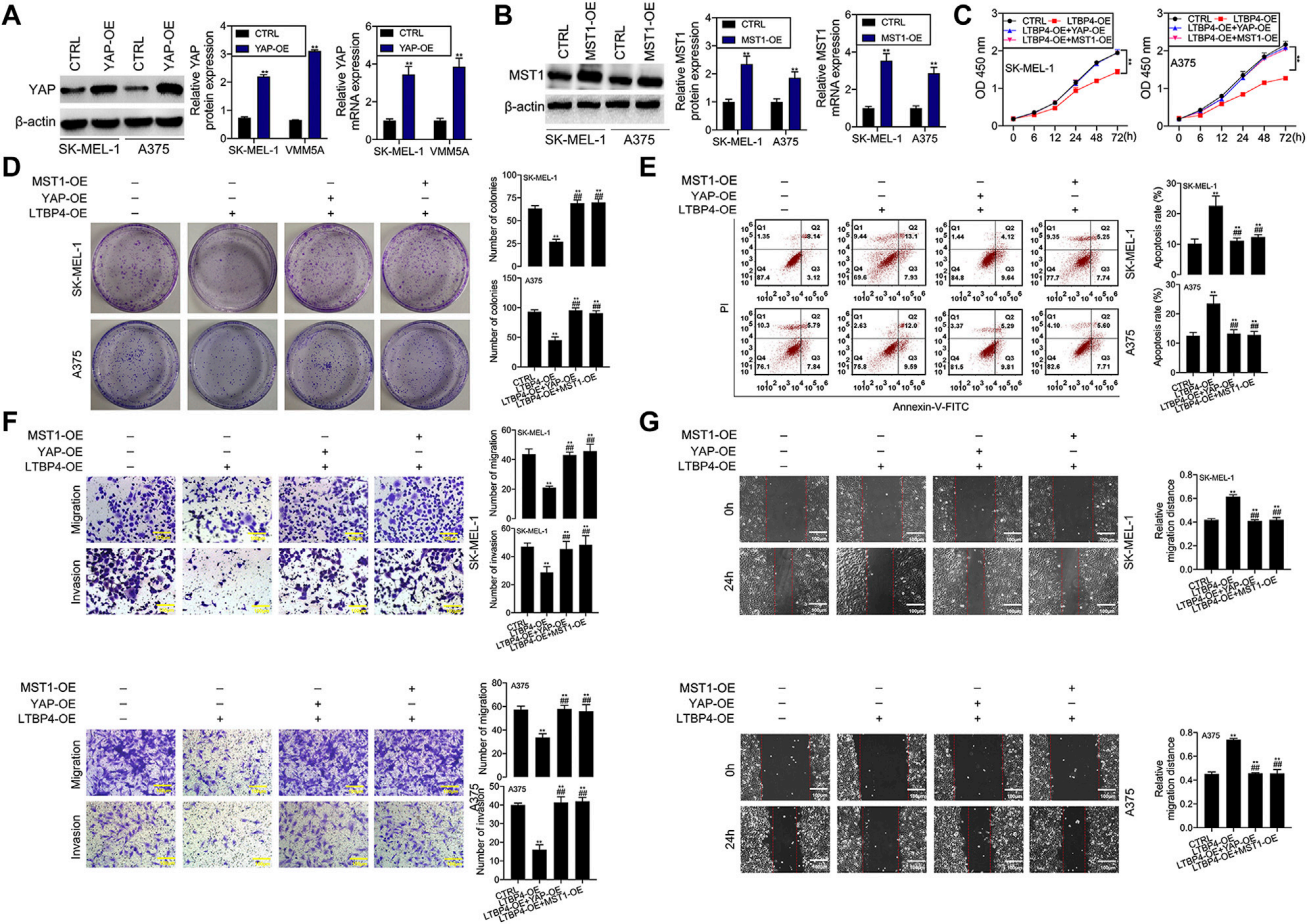
Overexpression of LTBP4 inhibited the proliferation, invasion and migration, and promoted the apoptosis of melanoma cells via the Hippo-YAP1 signaling. **(A)** The protein and mRNA levels of YAP1 in YAP1 expressing plasmids transfected SK-MEL-1 and A375 cells were detected by western blotting and RT-PCR assays. **(B)** The protein and mRNA levels of MST1 in MST1 expressing plasmids transfected SK-MEL-1 and A375 cells were detected by western blotting and RT-PCR assays. After LTBP4 expressing plasmids and YAP1/or MST1 expressing plasmids were co-transfected into SK-MEL-1 and A375 cells, **(C)** the viability was detected by CCK-8 assay, **(D)** the proliferation was detected by colony formation assay, **(E)** the apoptosis level was detected by flow cytometry assay, **(F)** the migration and invasion were detected by transwell assay, and **(G)** the migration was detected by wound healing assay. β-actin was used as a load control. Data are presented as the mean ± standard deviation. **p < 0.01 vs CTRL group and ^##^ *p* < 0.01 vs LTBP4-OE group.

**FIGURE 10:**
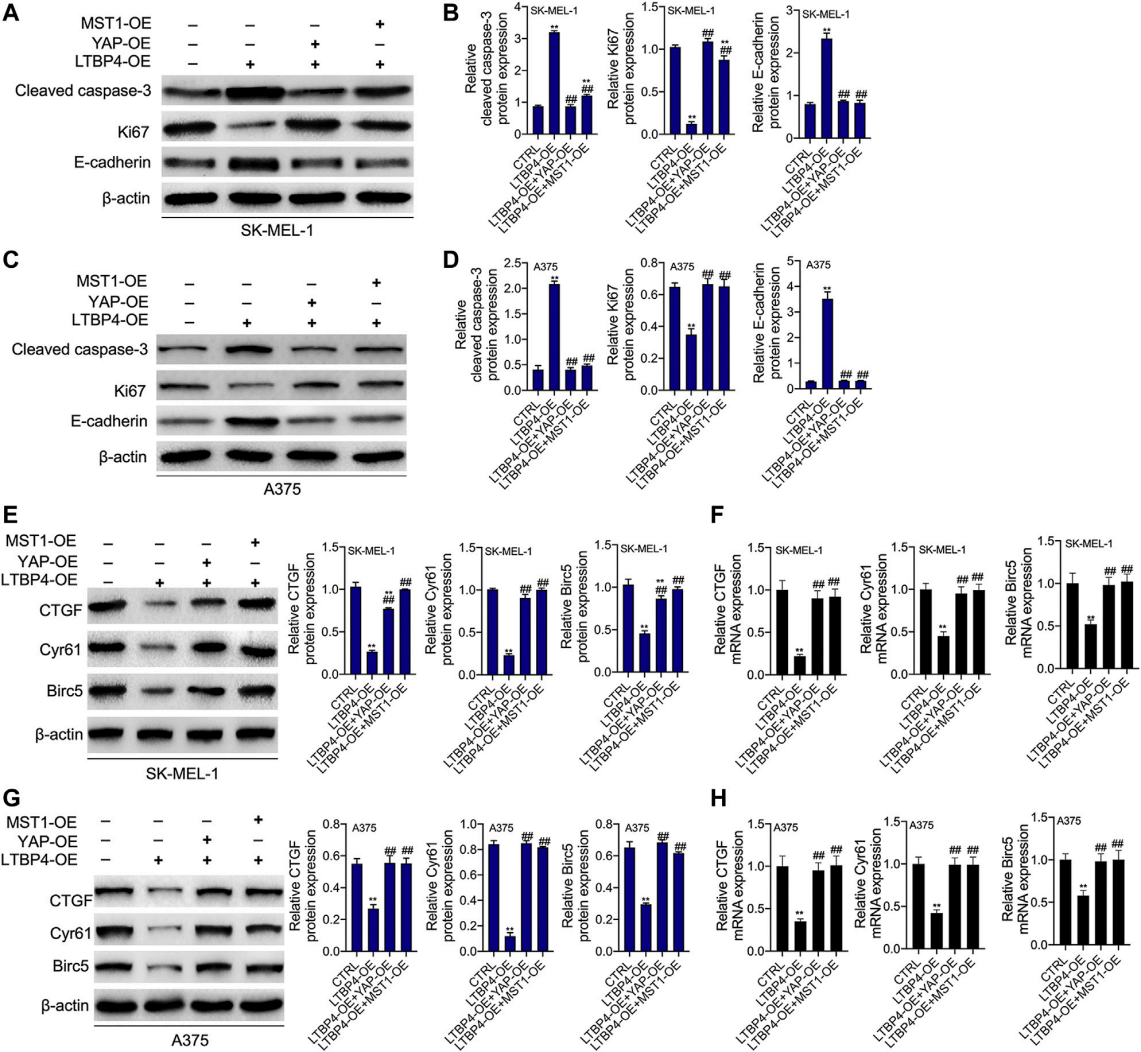
The functions of overexpression of LTBP4 regulating cleaved caspase-3, Ki67, E-cadherin, CTGF, Cyr61, and Birc5 were reversed by YAP1 OE or MST1 OE in melanoma cells. After LTBP4 expressing plasmids and YAP1/or MST1 expressing plasmids were co-transfected into SK-MEL-1 and A375 cells **(A** and **B)** the protein levels of cleaved caspase-3, Ki67, and E-cadherin in SK-MEL-1 cells were detected by western blotting assay **(C** and **D)** the protein levels of cleaved caspase-3, Ki67, and E-cadherin in A375 cells were detected by western blotting assay **(E** and **F)** the protein and mRNA levels of CTGF, Cyr61, and Birc5 in SK-MEL-1 cells were detected by western blotting and RT-PCR assays, and **(G** and **H)** the protein and mRNA levels of CTGF, Cyr61, and Birc5 in A375 cells were detected by western blotting and RT-PCR assays. β-actin was used as a load control. Data are presented as the mean ± standard deviation. **p < 0.01 vs CTRL group and ^##^ *p* < 0.01 vs LTBP4-OE group.

YAP1 or MST1 expression plasmid was transfected into SK-MEL-1 and A375 cells to induce the overexpression of YAP1 or MST1 by RT-PCR and Western blotting assays (Figures 9A,B). The results of CCK-8 and colony formation assays indicated that the functions of LTBP4 overexpression in inhibiting the viability and proliferation of SK-MEL-1 and A375 cells were reversed by YAP1 overexpression or MST1 overexpression (Figures 9C,D). In addition, YAP1 overexpression or MST1 overexpression significantly abolished the effects of LTBP4 overexpression on promoting the apoptosis of SK-MEL-1 and A375 cells (Figure 9E). In addition, the functions of LTBP4 overexpression in inhibiting the invasion and migration of SK-MEL-1 and A375 cells were reversed by YAP1 overexpression or MST1/2 overexpression (Figures 9F,G). Similarly, Western blotting results showed that the effects of LTBP4 overexpression on increasing the expression of cleaved caspase-3 and E-cadherin and reducing the Ki67 expression were counteracted by YAP1 overexpression or MST1 overexpression in SK-MEL-1 and A375 cells (Figures 10A–D). Next, both YAP1 overexpression and MST1 overexpression inhibited the functions of LTBP4 overexpression in decreasing CTGF, Cyr61, and Birc5 expression in SK-MEL-1 and A375 cells (Figures 10E–H).

## Discussion

The GEO database possesses expression profile data of a wide range of cancers, which has greatly reduced the time needed for the researchers to screen genes closely associated with cancer development (Song et al., 2020). We selected one melanoma-related dataset from the GEO data, namely GSE46517 (Xu et al., 2020). LASSO analysis and SVM-RFE analysis identified LTBP4 as a key feature biomarker for melanoma. Besides, results from the TCGaportal database revealed no significant difference in the survival between patients with low and high LTBP4 expression. However, our results indicated that patients with low LTBP4 expression had significantly lower survival than those with high LTBP4 expression. This may be ascribed to the small sample size (n = 76) and individual differences. However, the TCGaportal database also suggested that after 15 months, the survival rate of patients with low LTBP4 expression began to be significantly lower than that of patients with high LTBP4 expression. Based on the above results, it was confirmed that patients with low LTBP4 expression had poorer prognosis. Next, further experimental results indicated that LTBP4 showed significantly lower expression in tumor tissues from melanoma patients and melanoma cell lines. As revealed by cell and animal studies, LTBP4 silencing promoted cell proliferation, invasion, and migration, inhibited cell apoptosis, and significantly enhanced the tumorigenicity of melanoma cells, which in turn inhibited the progression of malignant melanoma. Of course, the expression of proteins related to proliferation, apoptosis, and migration, and invasion changed significantly during this process. Caspase-3 and Ki67 are the important proteins related to proliferation and apoptosis, which are abnormally expressed in numerous types of malignant tumors (Rodrigues et al., 2016). E-cadherin is a critical tumor suppressor gene related to tumor metastasis, whose expression is significantly down-regulated in malignant tumor cells with great migration and invasion (van Roy and Berx, 2008). Unsurprisingly, our results showed that LTBP4 silencing significantly inhibited the expression of cleaved caspase-3 and E-cadherin, but up-regulated that of Ki67 in melanoma cells, while these effects were abolished by LTBP4 overexpression, consistent with the above studies. These data suggest that LTBP4 is downregulated in malignant melanoma patients, which promotes tumor growth by accelerating cell proliferation, thus aggravating the occurrence and development of melanoma.

Studies have shown that LTBP4, a key molecule in the stabilization of the TGF-β receptor complex, can regulate the activity of TGF-β1, while TGF-β1 can regulate the nuclear translocation of YAP protein (Su et al., 2015; Patel et al., 2019). Thus, it is speculated that LTBP4 regulates the nuclear translocation of YAP protein by affecting TGF-β1 activity through acting on the TGF-β receptors, ultimately affecting the activation of the Hippo signaling pathway. The inactivation of the Hippo pathway fails to promote the phosphorylation of YAP protein so that cell proliferation is promoted (Boopathy and Hong, 2019). The high expression of YAP has been reported to promote tumor growth and accelerate cancer progression (Zanconato et al., 2016; Mello et al., 2017; Huang et al., 2020). In this study, tumor formation experiments in nude mice showed that YAP was highly expressed in the cytoplasm and nuclei of tumor tissues in mice transfected with LTBP4-silencing cells. In addition, LTBP4 silencing also increased the expression of YAP and inhibited its phosphorylation. At the same time, expression of the downstream transcriptional activators of the Hippo signaling pathway, including CTGF, CYR61, and BIRC5 (Wu et al., 2020b), elevated by LTBP4 silencing. Certainly, the effect of LTBP4 overexpression was also investigated in the above processes, and the results showed that high expression of LTBP4 promoted the phosphorylation of YAP, but inhibited the expression of CTGF, CYR61, and BIRC5. Therefore, these data confirm that LTBP4 plays an anticancer role in melanoma by promoting YAP phosphorylation to activate the Hippo signaling pathway, thereby inhibiting tumor growth and metastasis. However, the unphosphorylated YAP will translocate into the nucleus and accumulate in the nucleus, where it plays a role of transcriptional co-activator to promote the expression of related genes that contribute to cell proliferation and survival (Hasegawa et al., 2021). Our results also showed that LTBP4 silencing promoted the nuclear translocation of YAP and increased the enrichment of YAP in the nucleus. The nuclear enrichment of YAP also promoted the expression of CTGF, CYR61, and BIRC5. In addition, LTBP4 silencing inhibited the expression of TGFβR2 and induced the activation of TGFβ1. The functions of TGFβ1 treatment in stimulating YAP1 nuclear-cytoplasmic translocation and inhibiting the activation of the Hippo signaling were abolished by LTBP4 overexpression. This directly indicates that the active TGF-β1 enhances its role in promoting the cytoplasmic-nuclear translocation of YAP1. Moreover, rescue experimental results suggested that the functions of LTBP4 overexpression in inhibiting the viability, proliferation, invasion, and migration, and promoting the apoptosis of melanoma cells were reversed by YAP1 overexpression or MST1 overexpression (the core molecule of Hippo signaling pathway). Meanwhile, the functions of LTBP4 overexpression in inhibiting the expression of cleaved caspase-3 and E-cadherin and inhibiting that of Ki67, CTGF, Cyr61 and Birc5 in melanoma cells were abolished by YAP1 overexpression or MST1 overexpression. Therefore, these results indicate that LTBP4 activates TGF-β1 through co-expression with TGFβR2. It then promotes the phosphorylation of YAP and reduces its nuclear translocation, eventually inhibiting its nuclear enrichment to lead to the activation of the Hippo signaling pathway, which slows the pathogenesis of skin melanoma.

## Conclusion

The downregulation of LTBP4 is found in melanoma tissues and cell lines, which predicts poor survival of patients with melanoma. LTBP4 silencing promotes the growth and metastasis of melanoma *in vivo* and *in vitro*. We further prove that LTBP4 knockdown increases YAP1 transcriptional activity and promotes YAP1 cytoplasmic-nuclear translocation by inducing the secretion of the active TGFβ1. Therefore, LTBP4 regulates the progression of skin melanoma via the TGFβ1/Hippo/YAP1 signaling pathway. These results also raise the possibility that LTBP4 may function as an important new biomarker

## Data Availability

The datasets presented in this study can be found in online repositories. The names of the repository/repositories and accession number(s) can be found in the article/Supplementary Material.

## Abbreviations

GEO database, Gene Expression Omnibus database; LTBP4, Latent transforming growth factor beta binding protein 4; DEG, Differentially expression gene; OS, Overall survival; OE, Overexpression; KD, Knockdown; YAP. Yes1-associated transcriptional regulator; MST1, Macrophage stimulating 1; MOB1, Mps one binder protein 1; CTGF, Connective tissue growth factor; Birc5, Baculoviral IAP repeat containing 5; TGF-β, Transforming growth factor-β; TGF-βRII, Transforming growth factor-β receptor II.
