# Supplementary figures and images for: Disruption of LTBP4 Inhibition-Induced TGFβ1 Activation Promoted Cell Proliferation and Metastasis in Skin Melanoma by Inhibiting the Activation of the Hippo-YAP1 Signaling Pathway
Source: Front Cell Dev Biol. 2022 Feb 17;9:673904. doi: 10.3389/fcell.2021.673904 (PMC8893603; doi:10.3389/fcell.2021.673904)

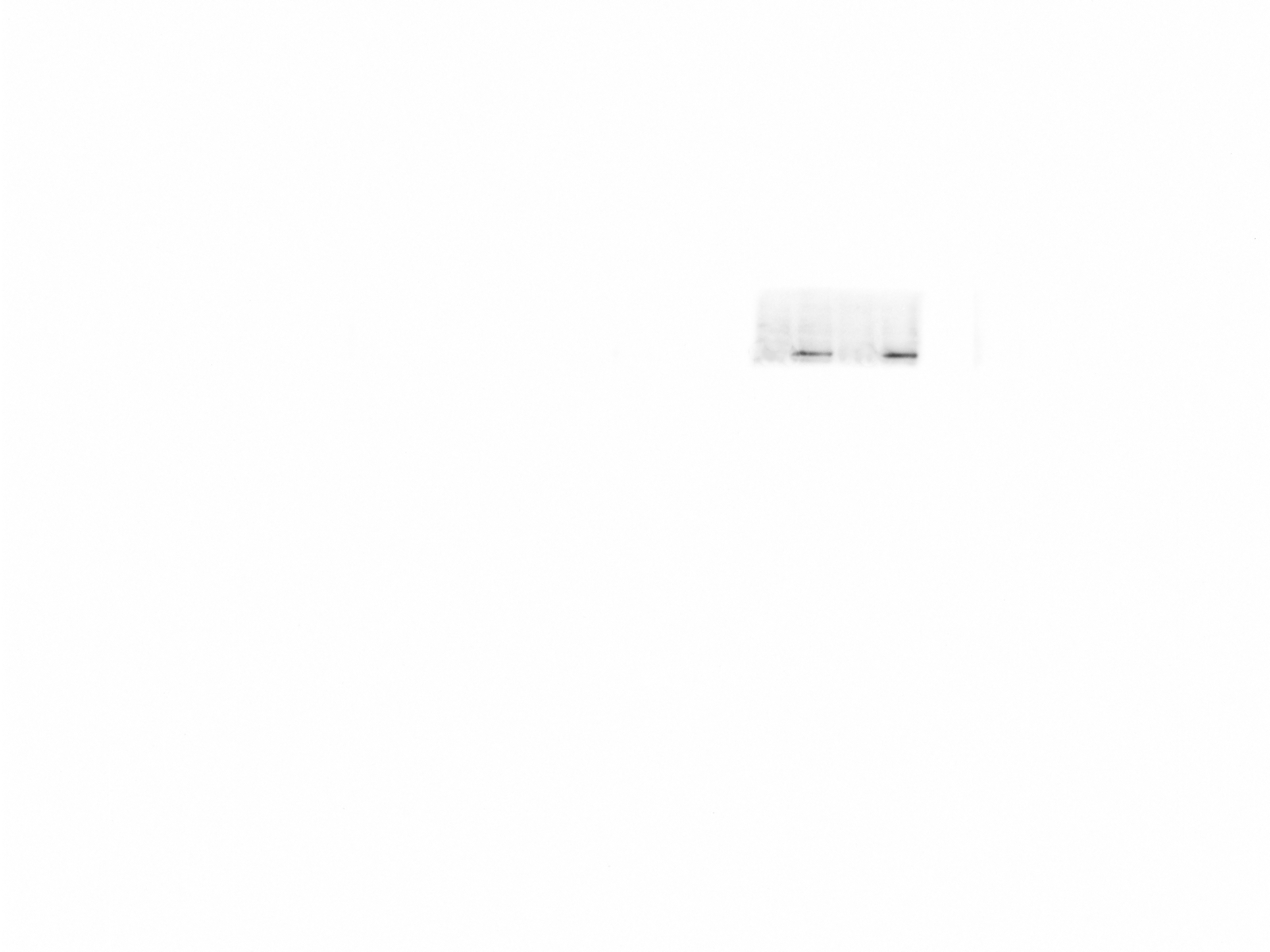

Supplement: Supplementary file 1 [file DataSheet1.zip › Suppl. Figs 1-7/7/13.tif]

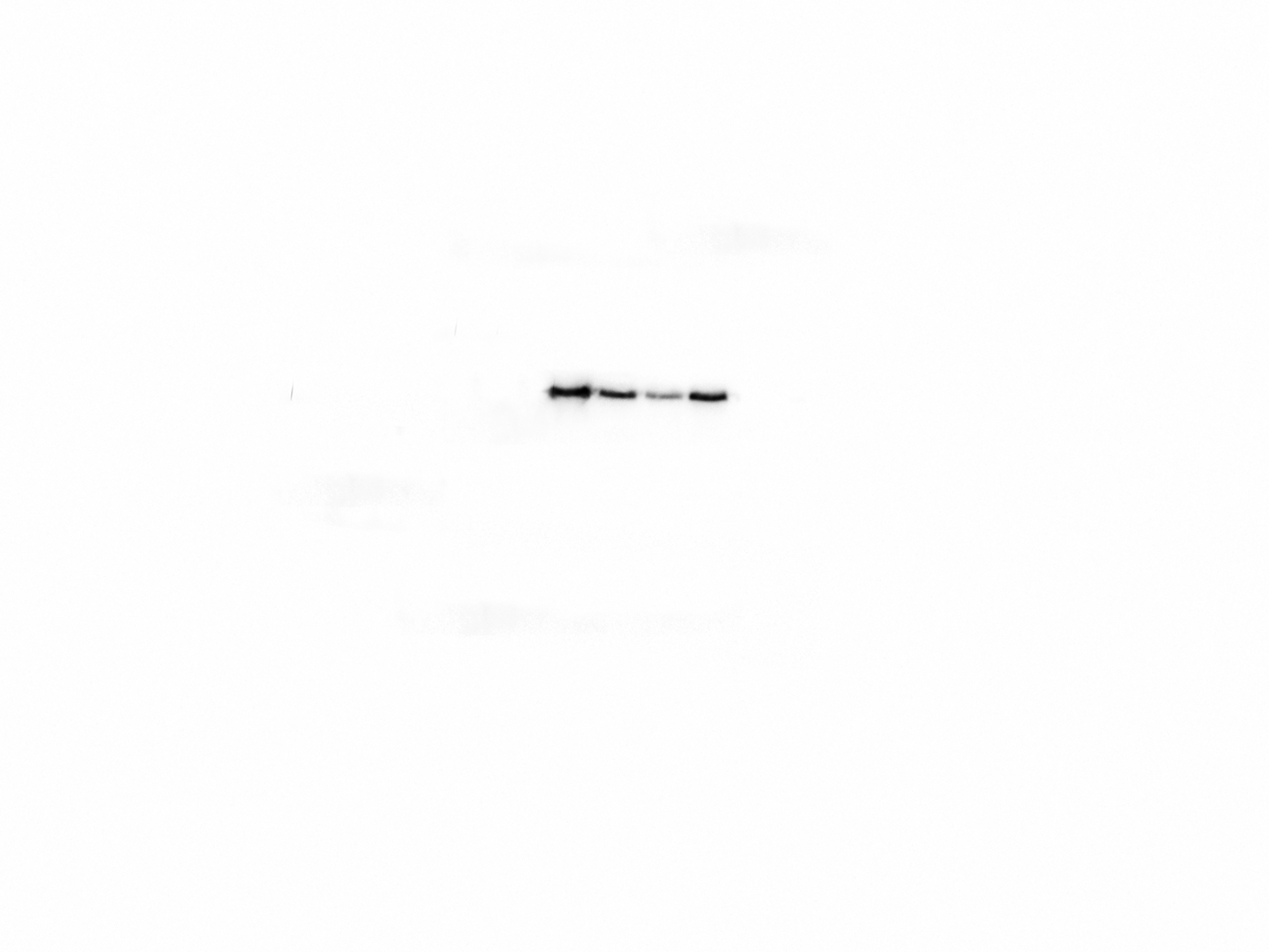

Supplement: Supplementary file 1 [file DataSheet1.zip › Suppl. Figs 1-7/7/12.tif]

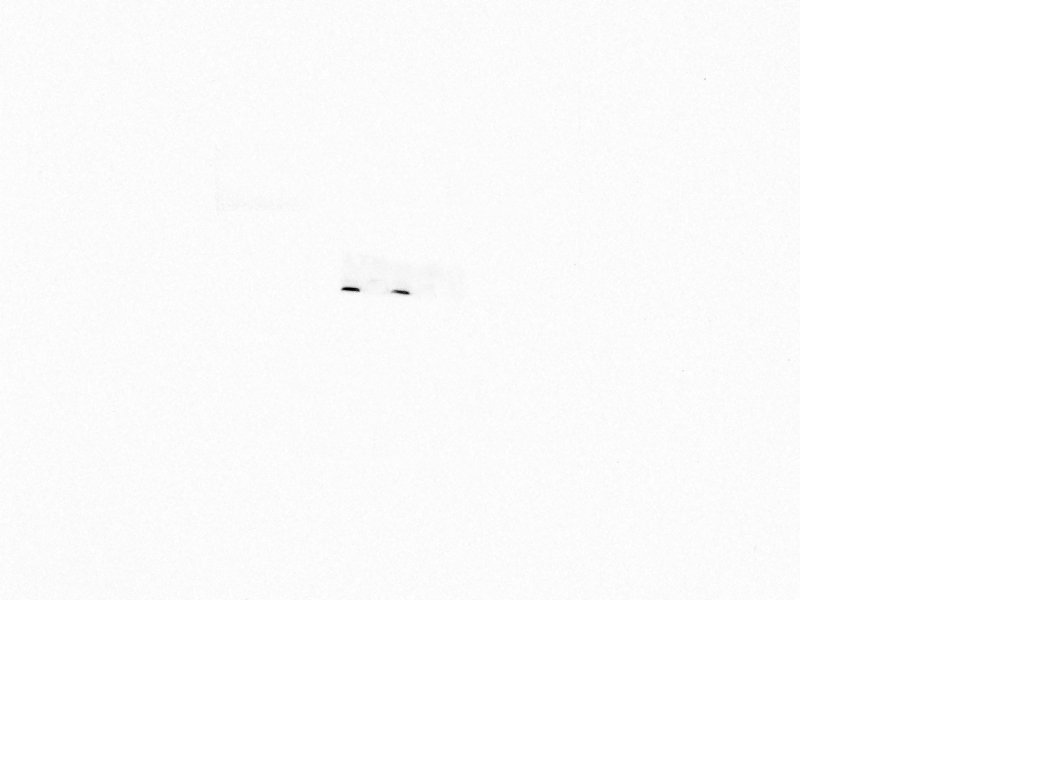

Supplement: Supplementary file 1 [file DataSheet1.zip › Suppl. Figs 1-7/7/3.tif]

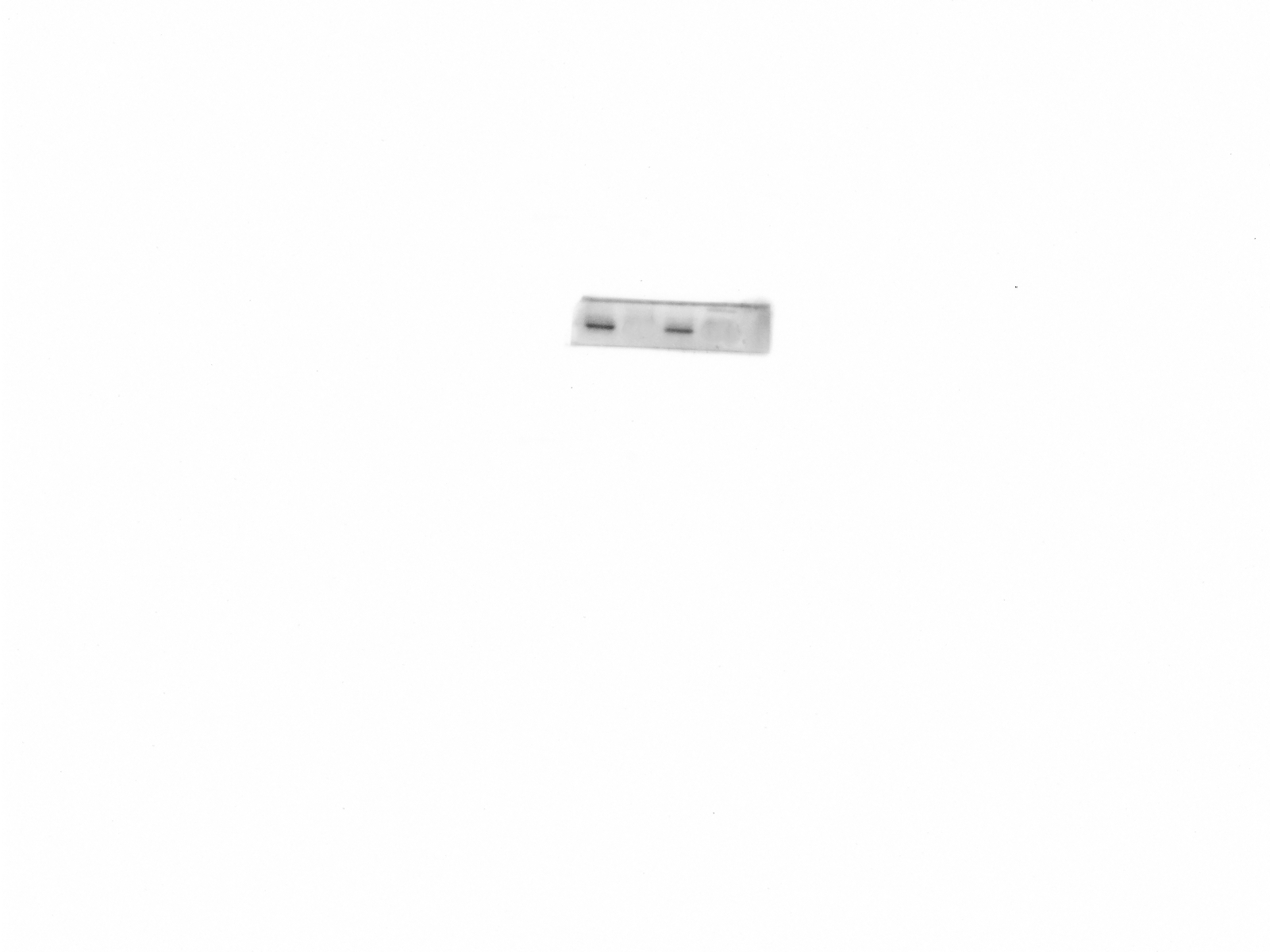

Supplement: Supplementary file 1 [file DataSheet1.zip › Suppl. Figs 1-7/7/5.tif]

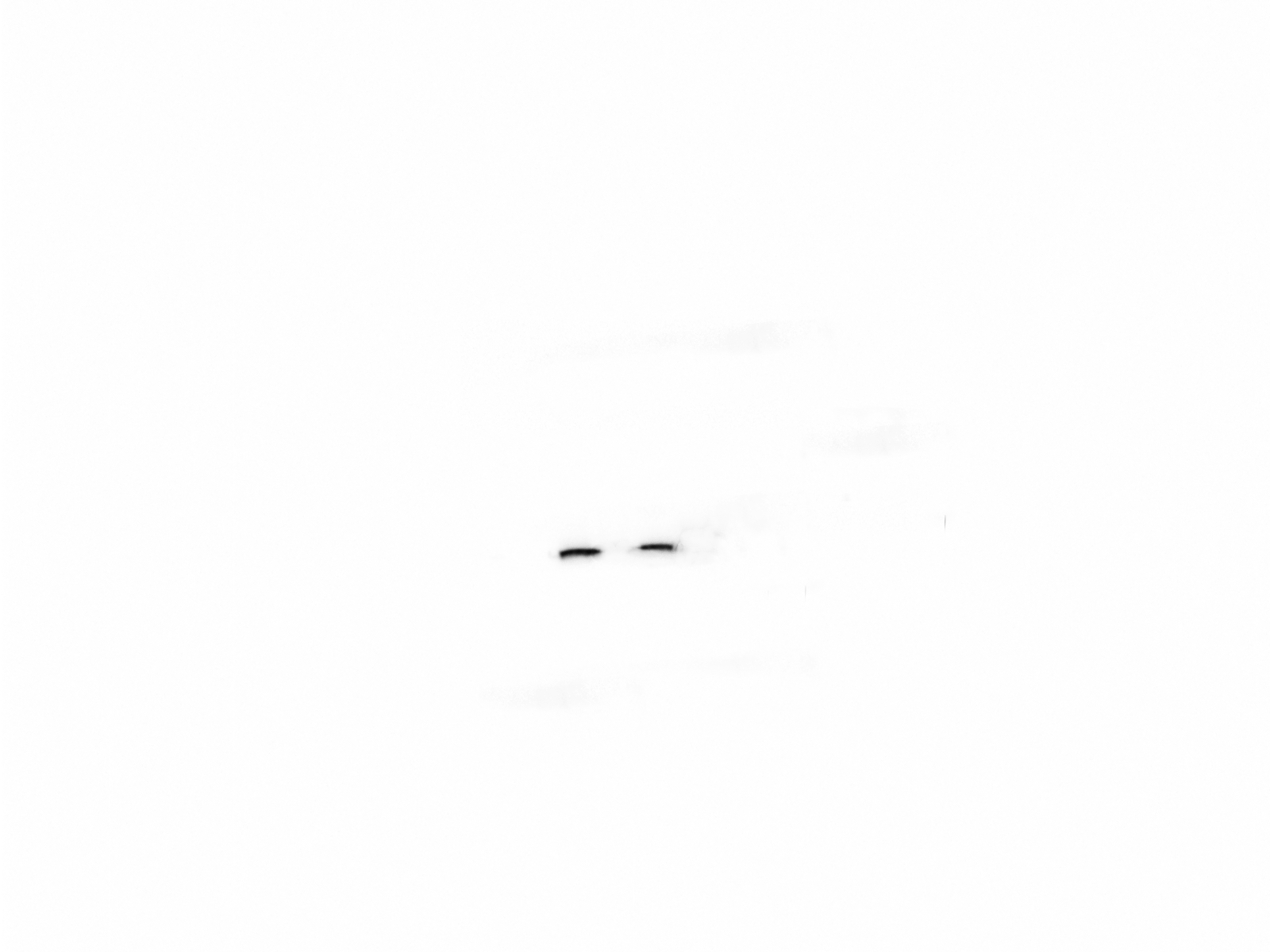

Supplement: Supplementary file 1 [file DataSheet1.zip › Suppl. Figs 1-7/7/4.tif]

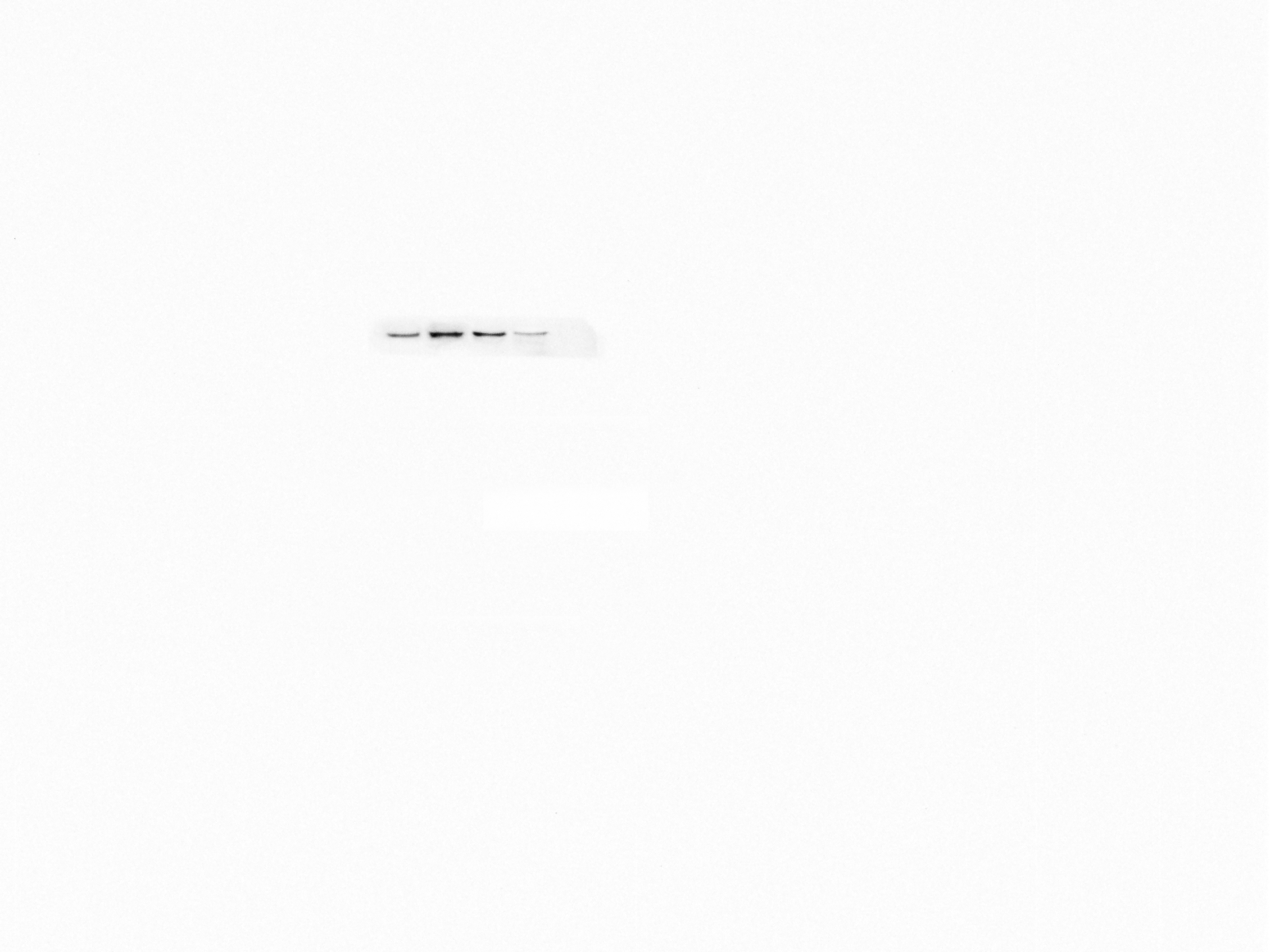

Supplement: Supplementary file 1 [file DataSheet1.zip › Suppl. Figs 1-7/7/6.tif]

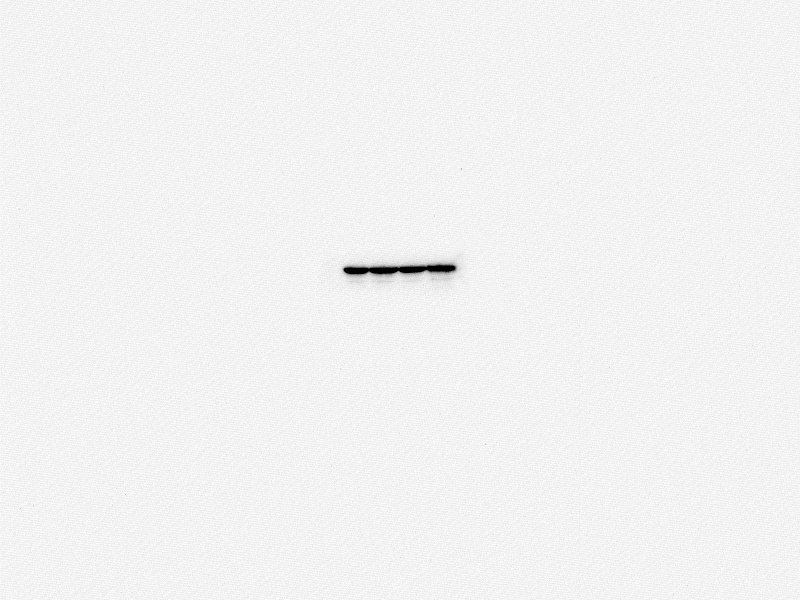

Supplement: Supplementary file 1 [file DataSheet1.zip › Suppl. Figs 1-7/7/7.tif]

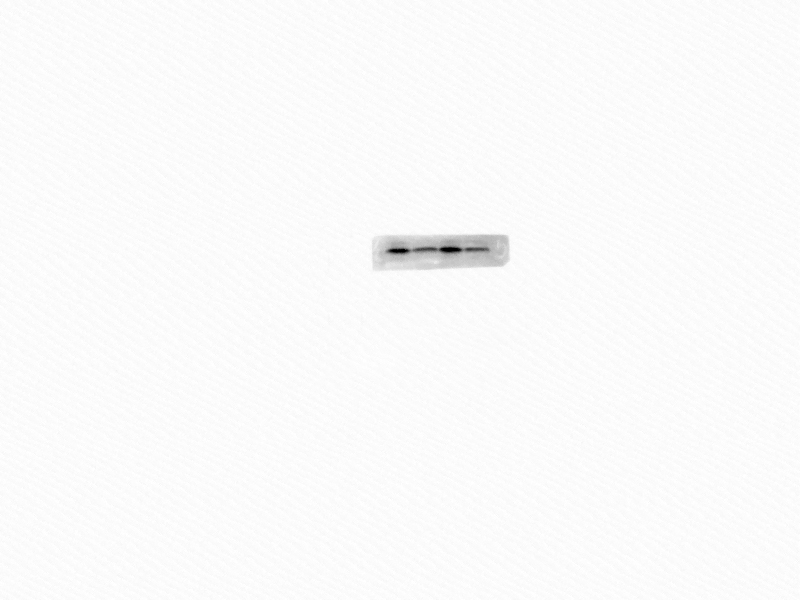

Supplement: Supplementary file 1 [file DataSheet1.zip › Suppl. Figs 1-7/6/MOB1.tif]

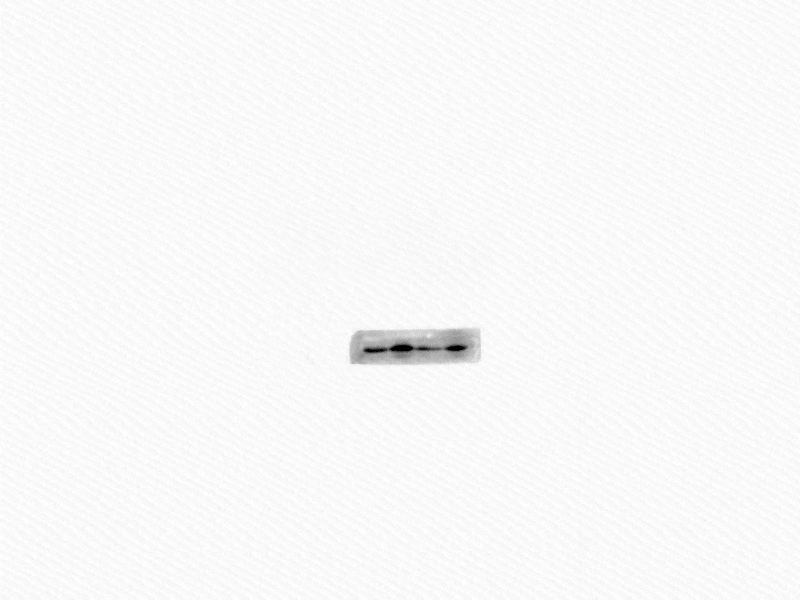

Supplement: Supplementary file 1 [file DataSheet1.zip › Suppl. Figs 1-7/6/p-MST1.tif]

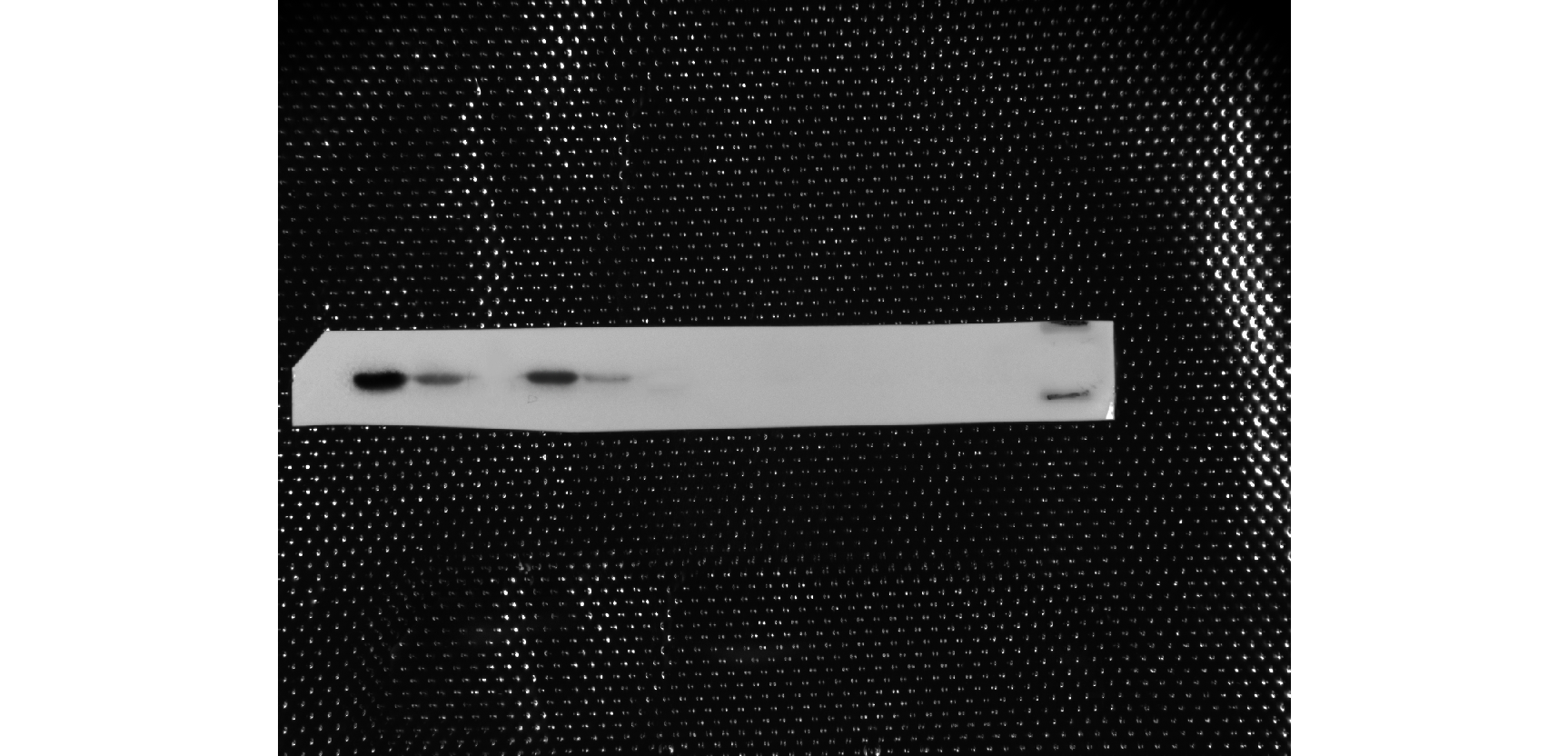

Supplement: Supplementary file 1 [file DataSheet1.zip › Suppl. Figs 1-7/6/YAP 2.tif]

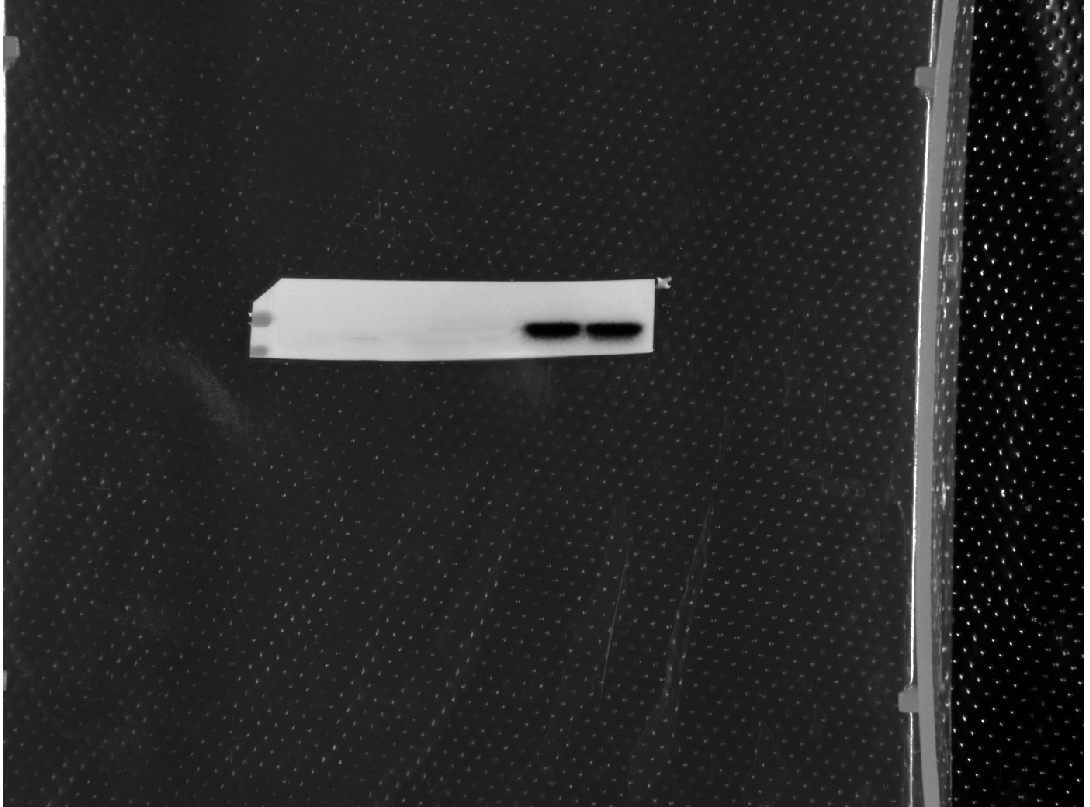

Supplement: Supplementary file 1 [file DataSheet1.zip › Suppl. Figs 1-7/6/actin 2.tif]

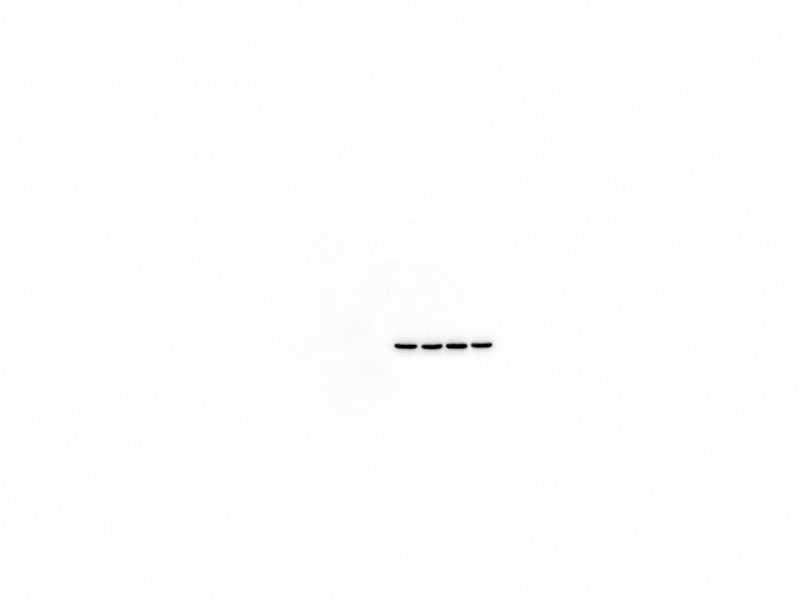

Supplement: Supplementary file 1 [file DataSheet1.zip › Suppl. Figs 1-7/6/LaminB.tif]

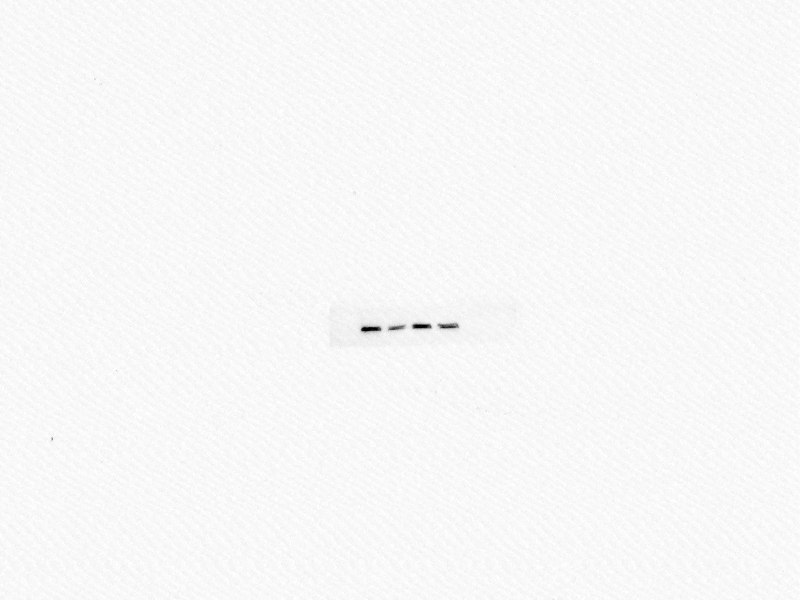

Supplement: Supplementary file 1 [file DataSheet1.zip › Suppl. Figs 1-7/6/Birc5-d.tif]

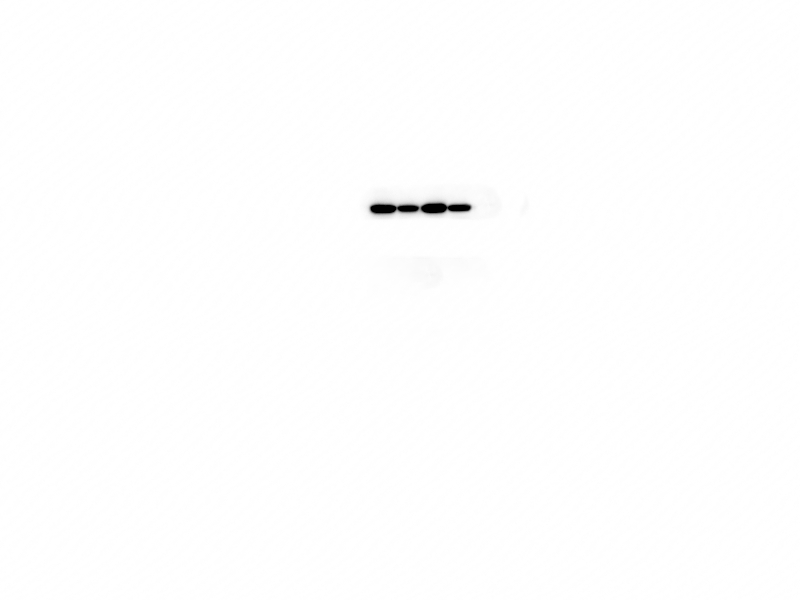

Supplement: Supplementary file 1 [file DataSheet1.zip › Suppl. Figs 1-7/6/CTGF-d.tif]

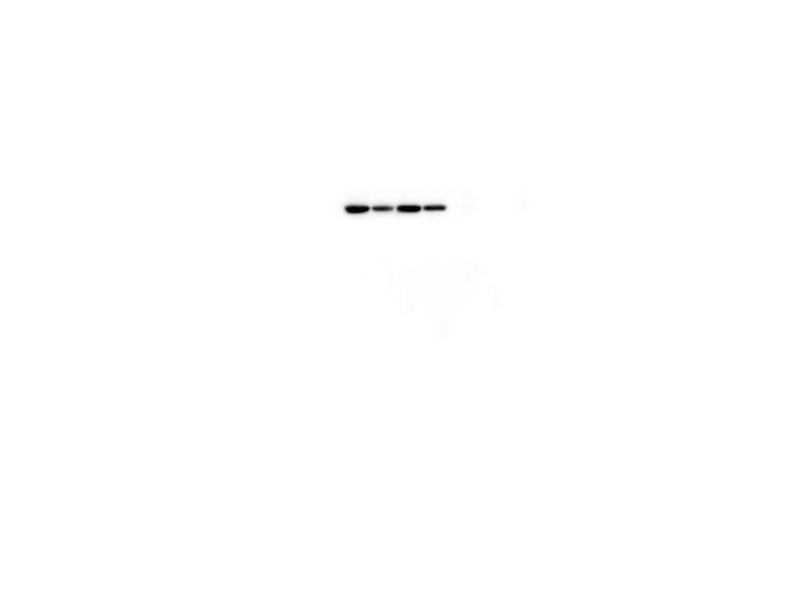

Supplement: Supplementary file 1 [file DataSheet1.zip › Suppl. Figs 1-7/6/YAP-f.tif]

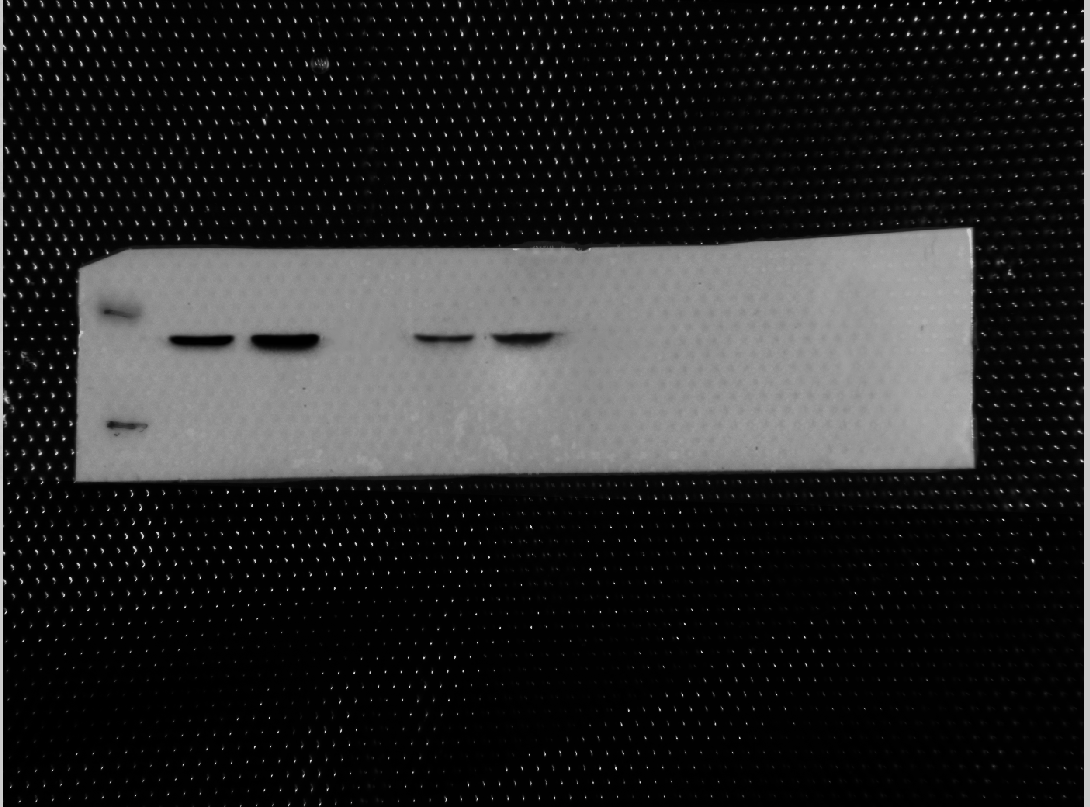

Supplement: Supplementary file 1 [file DataSheet1.zip › Suppl. Figs 1-7/6/p-YAP.tif]

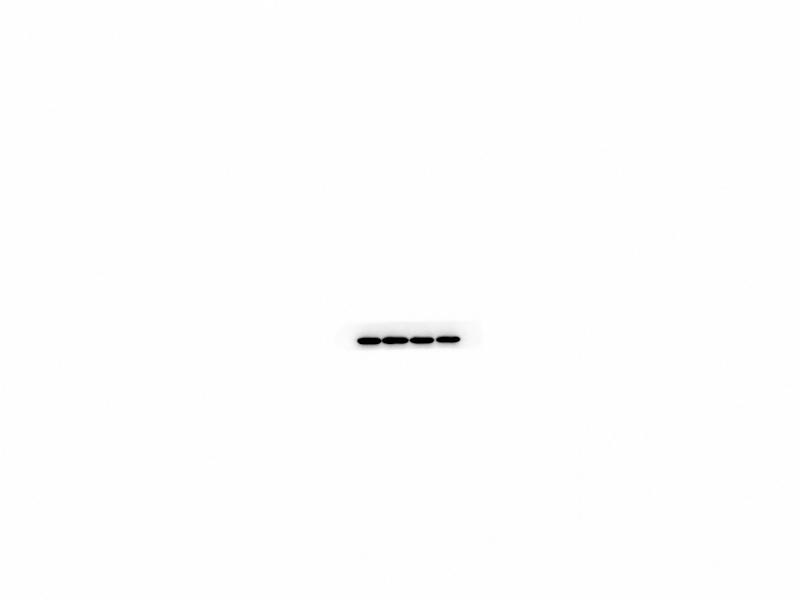

Supplement: Supplementary file 1 [file DataSheet1.zip › Suppl. Figs 1-7/6/actin-d.tif]

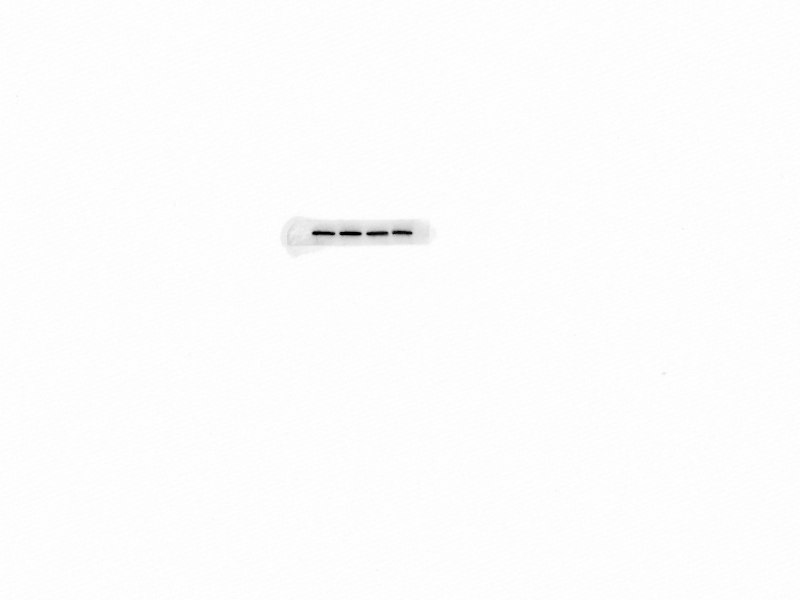

Supplement: Supplementary file 1 [file DataSheet1.zip › Suppl. Figs 1-7/6/actin.tif]

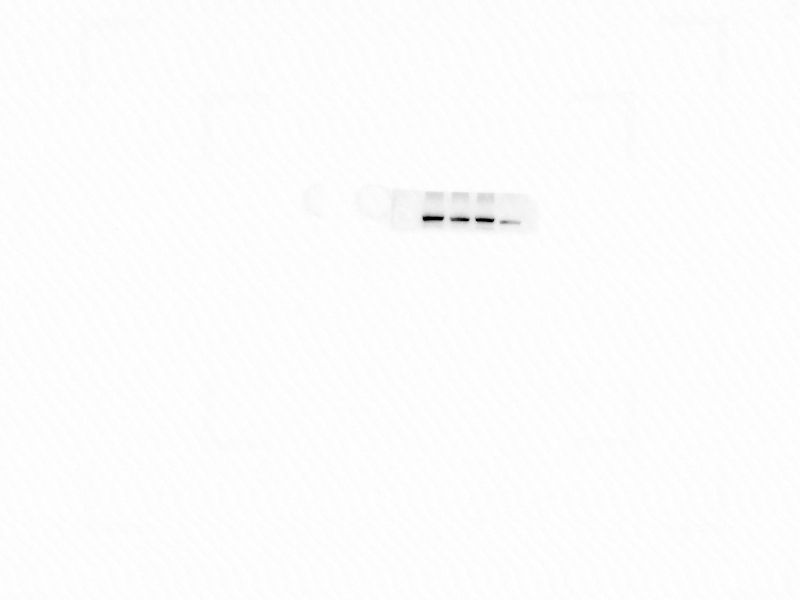

Supplement: Supplementary file 1 [file DataSheet1.zip › Suppl. Figs 1-7/6/Cyr61-d.tif]

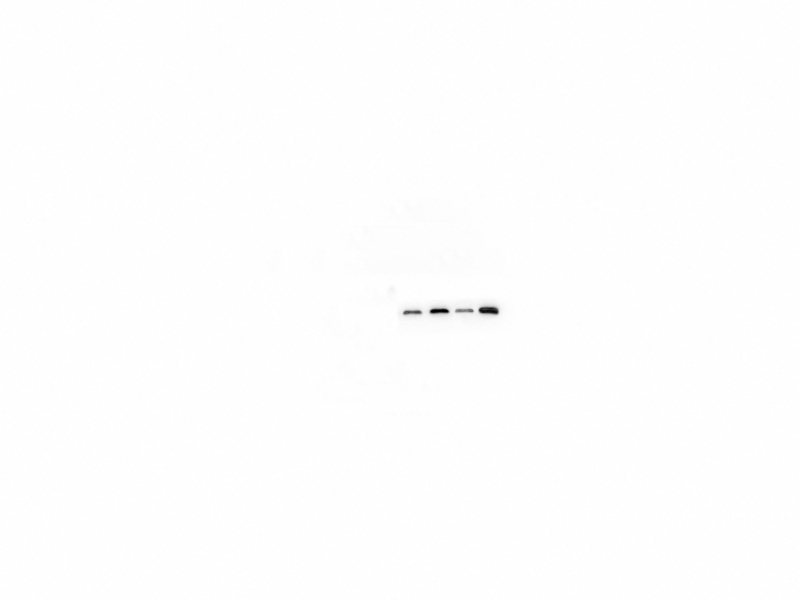

Supplement: Supplementary file 1 [file DataSheet1.zip › Suppl. Figs 1-7/6/p-MOB1.tif]

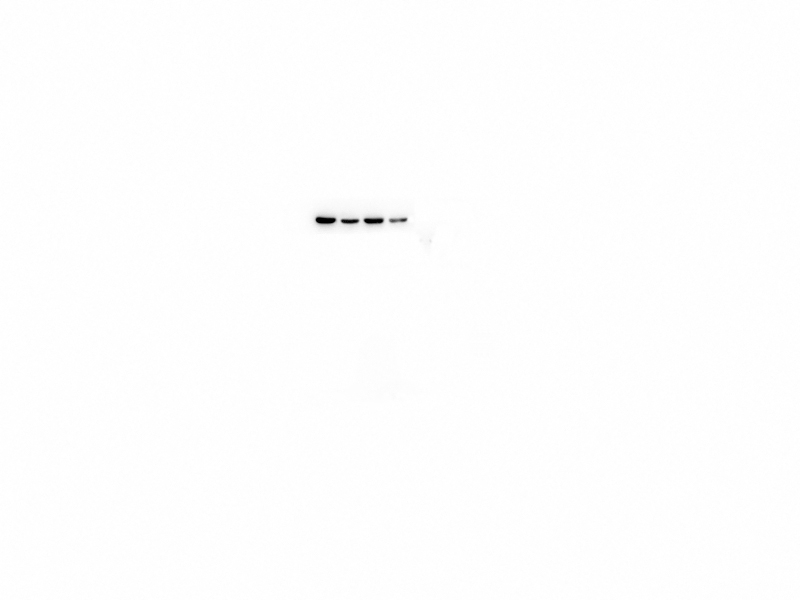

Supplement: Supplementary file 1 [file DataSheet1.zip › Suppl. Figs 1-7/6/MST1.tif]

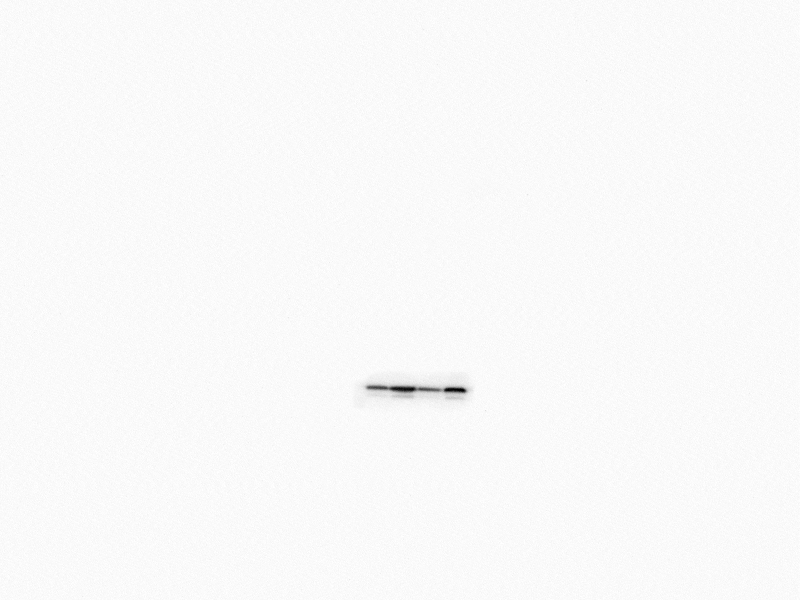

Supplement: Supplementary file 1 [file DataSheet1.zip › Suppl. Figs 1-7/6/YAP.tif]

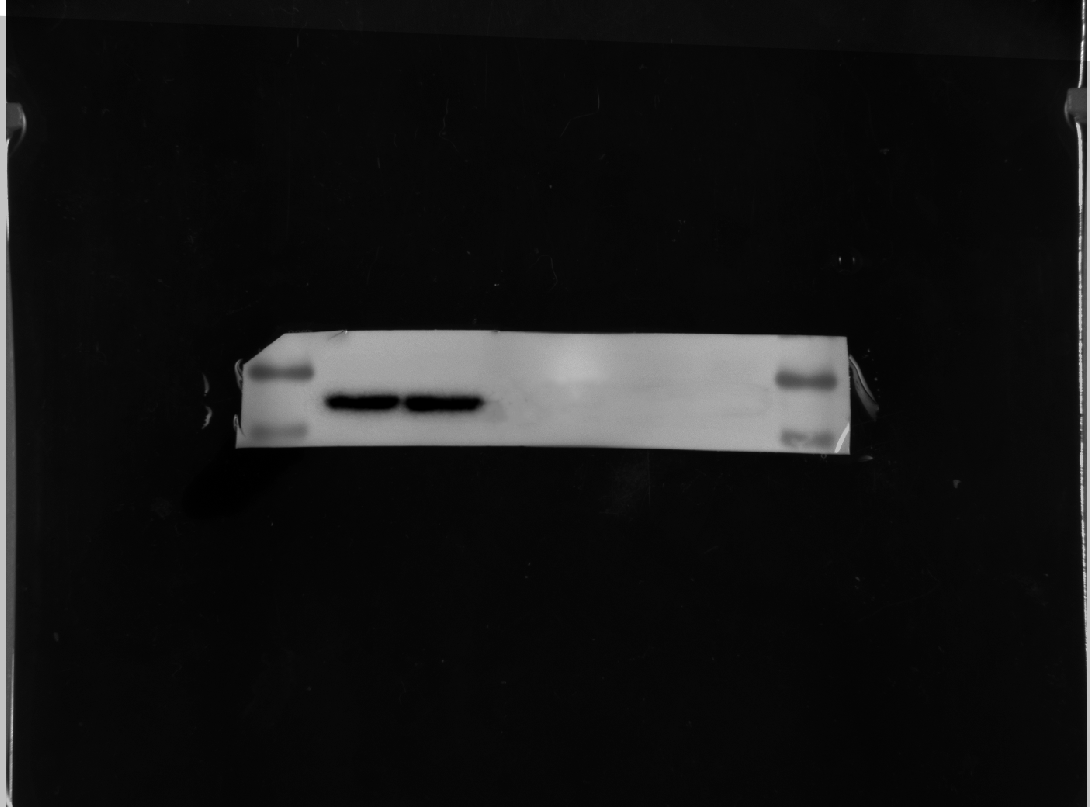

Supplement: Supplementary file 1 [file DataSheet1.zip › Suppl. Figs 1-7/6/╬▓-actin-2.tif]

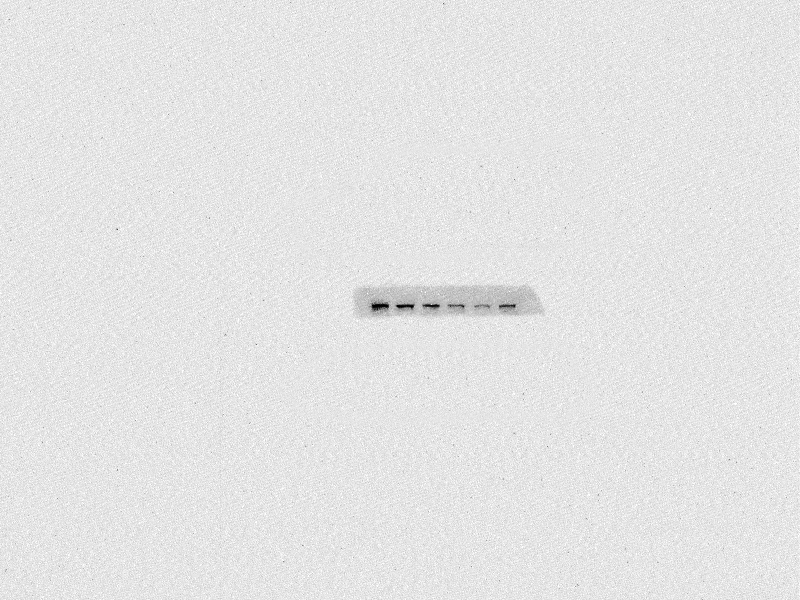

Supplement: Supplementary file 1 [file DataSheet1.zip › Suppl. Figs 1-7/1/LTBP4-f.tif]

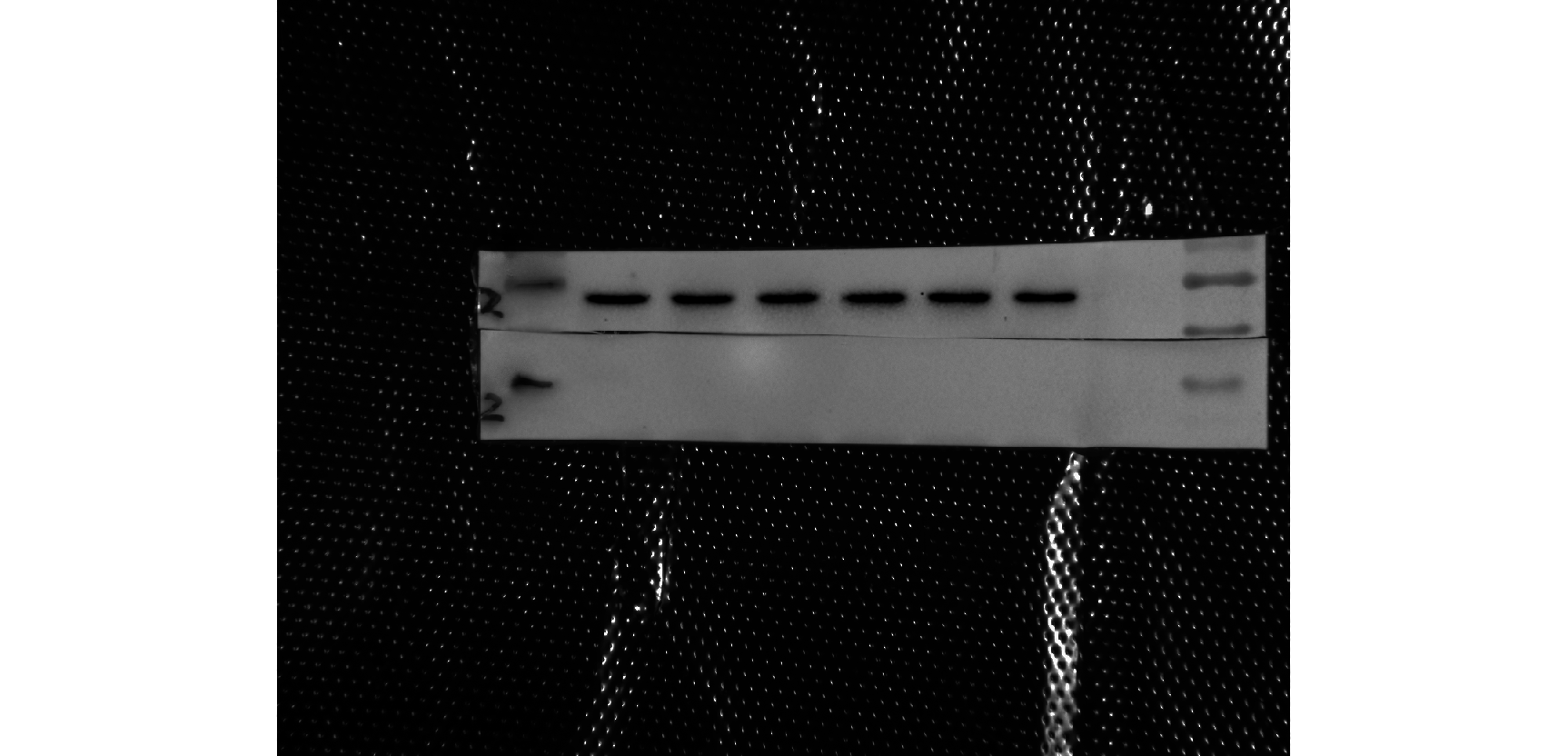

Supplement: Supplementary file 1 [file DataSheet1.zip › Suppl. Figs 1-7/1/╬▓-actin-h.tif]

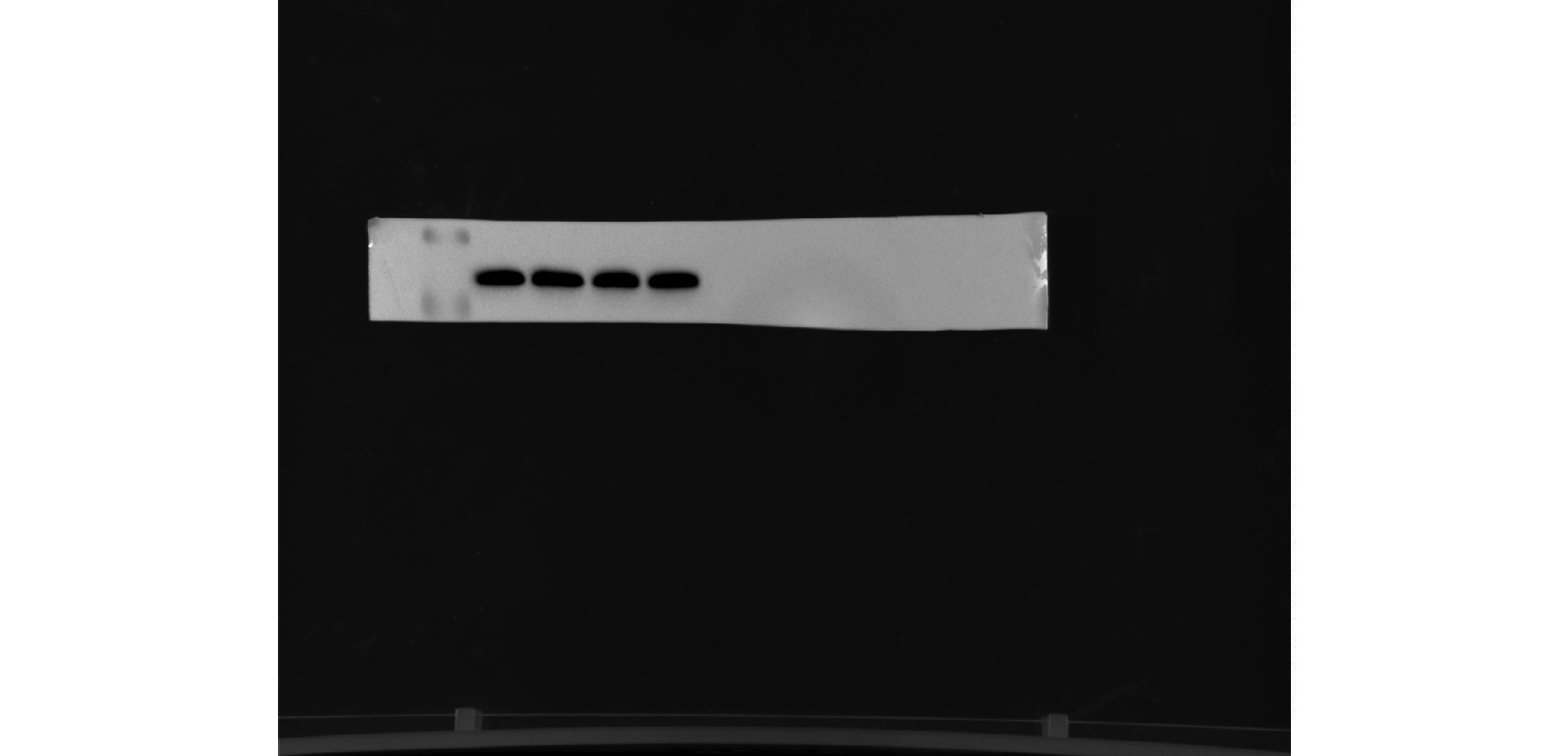

Supplement: Supplementary file 1 [file DataSheet1.zip › Suppl. Figs 1-7/1/╬▓-actin-i.tif]

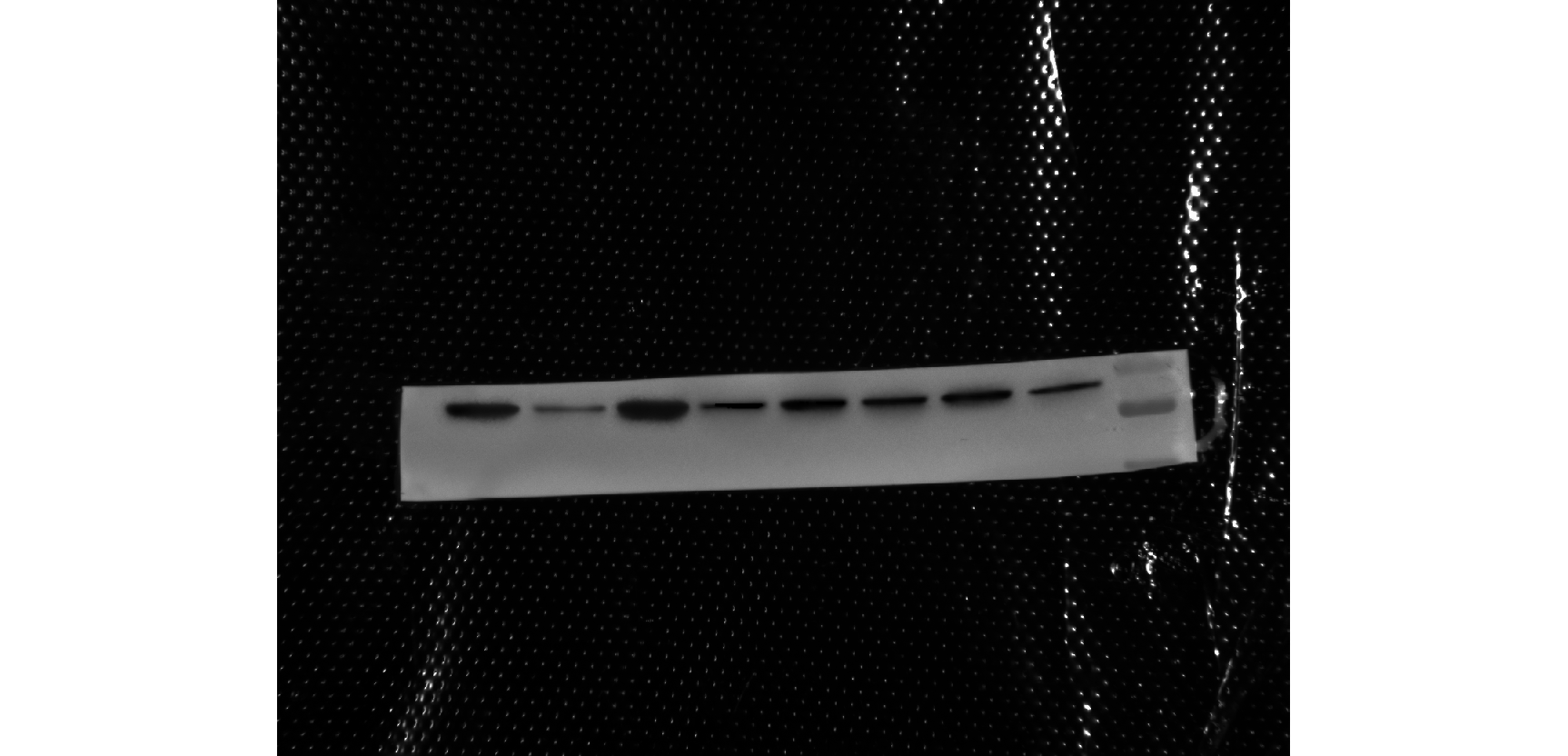

Supplement: Supplementary file 1 [file DataSheet1.zip › Suppl. Figs 1-7/1/LTPB4.tif]

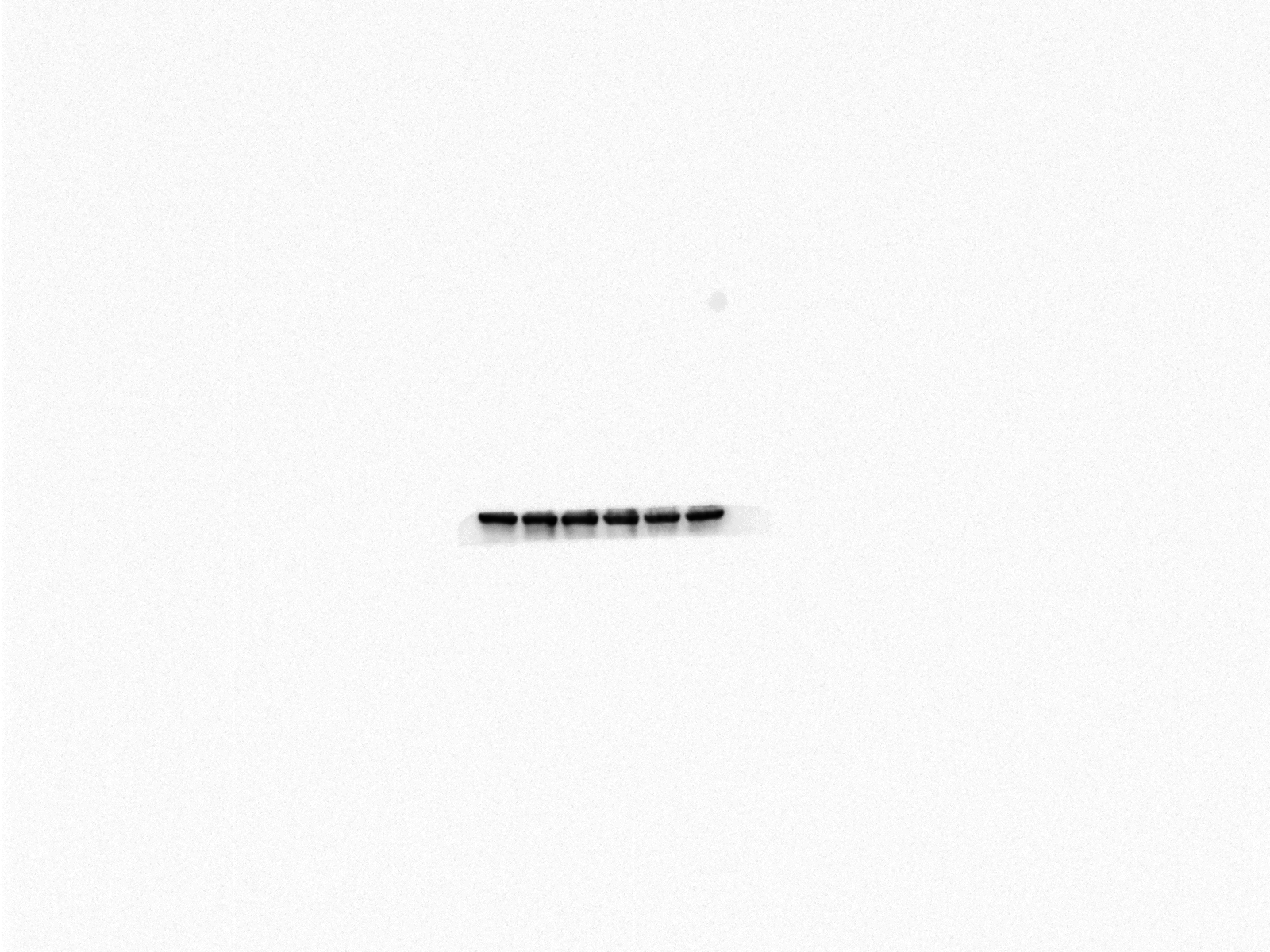

Supplement: Supplementary file 1 [file DataSheet1.zip › Suppl. Figs 1-7/1/╬▓-actin-f.tif]

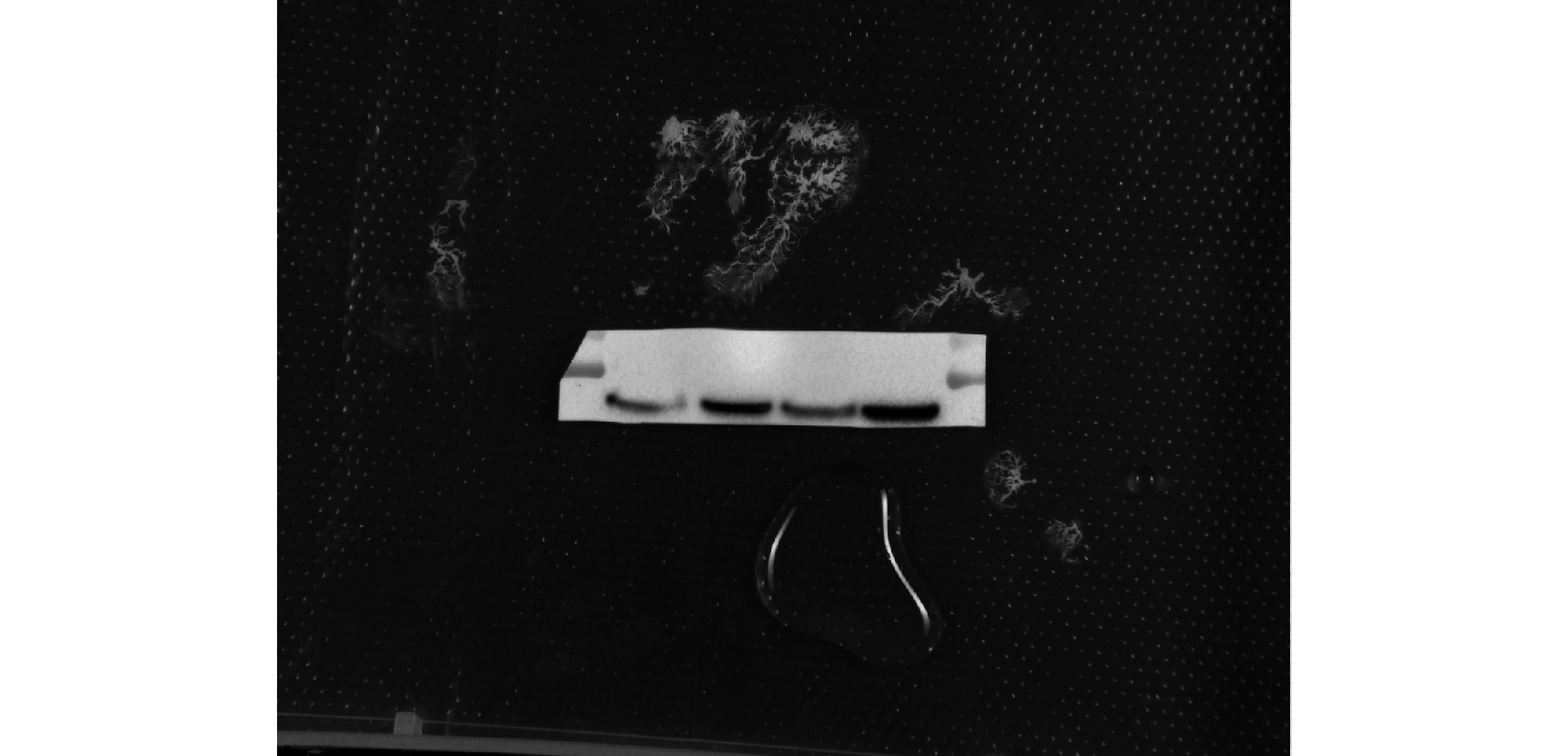

Supplement: Supplementary file 1 [file DataSheet1.zip › Suppl. Figs 1-7/1/LTBP4-i.tif]

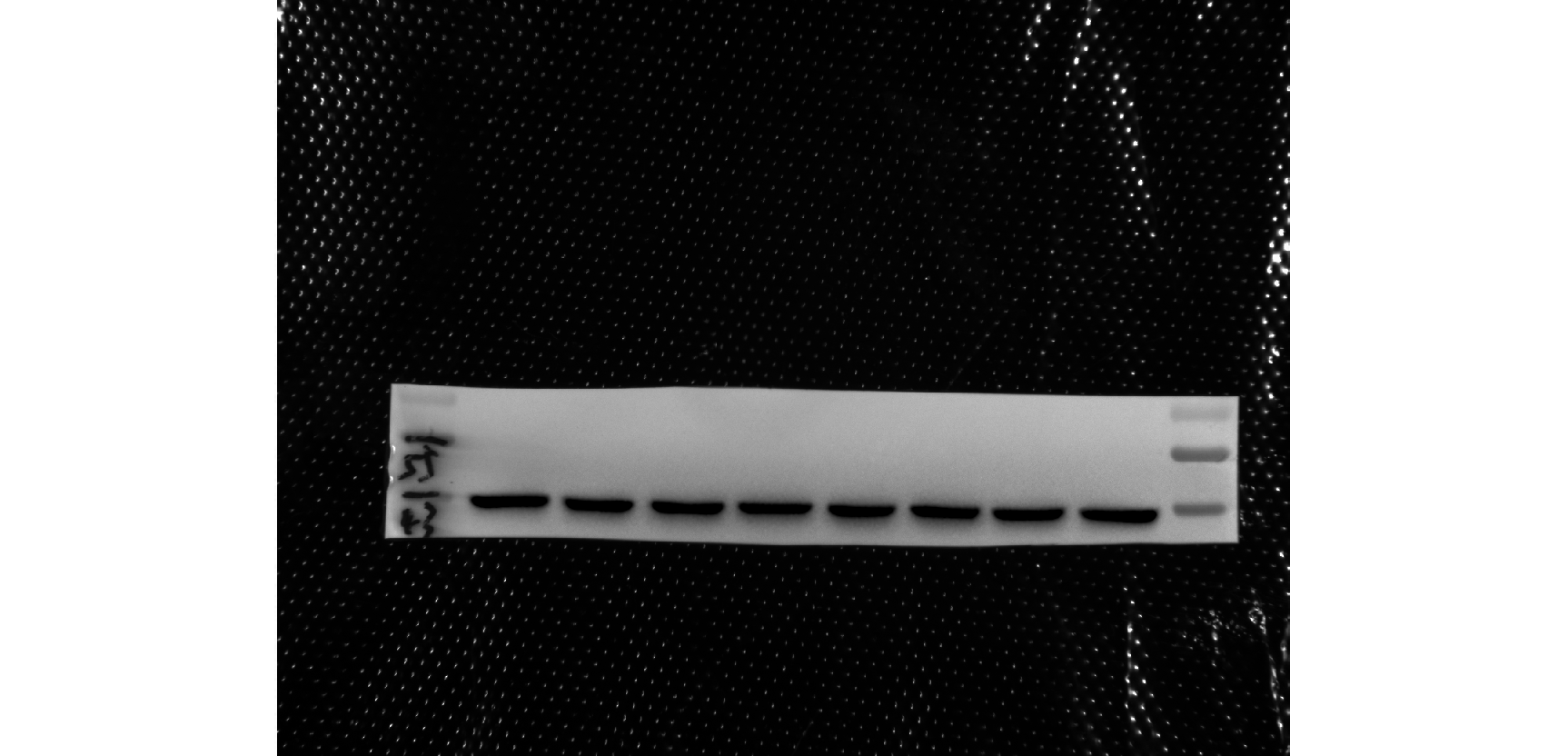

Supplement: Supplementary file 1 [file DataSheet1.zip › Suppl. Figs 1-7/1/╬▓-actin 2.tif]

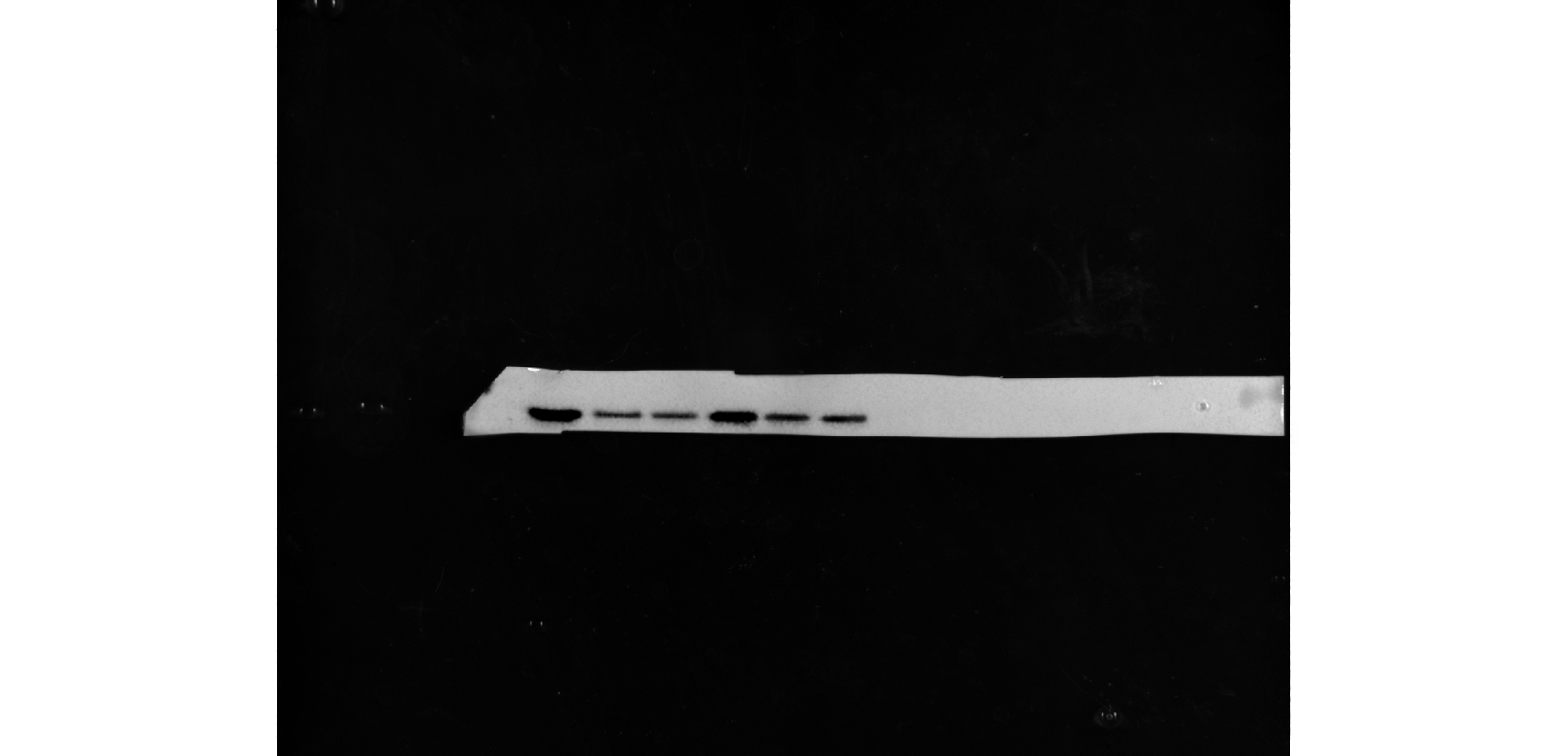

Supplement: Supplementary file 1 [file DataSheet1.zip › Suppl. Figs 1-7/1/LTBP4-h.tif]

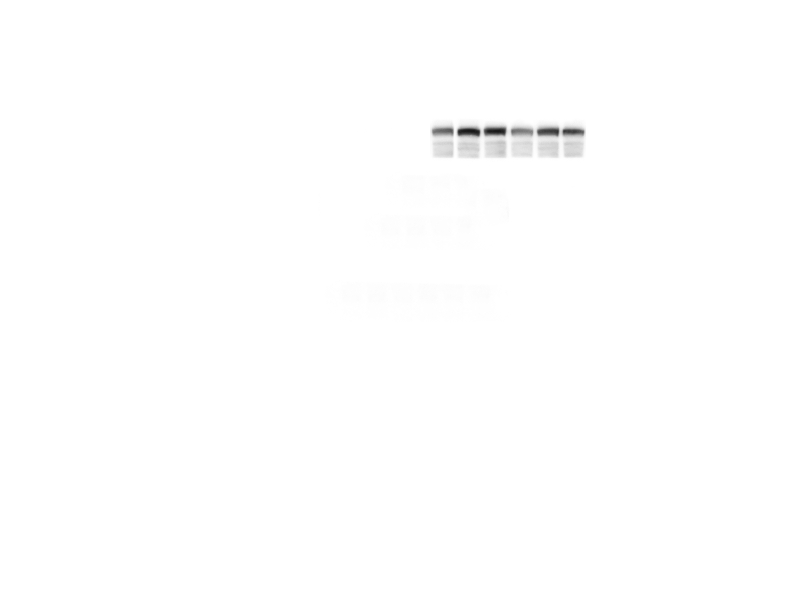

Supplement: Supplementary file 1 [file DataSheet1.zip › Suppl. Figs 1-7/4/Cyr61.tif]

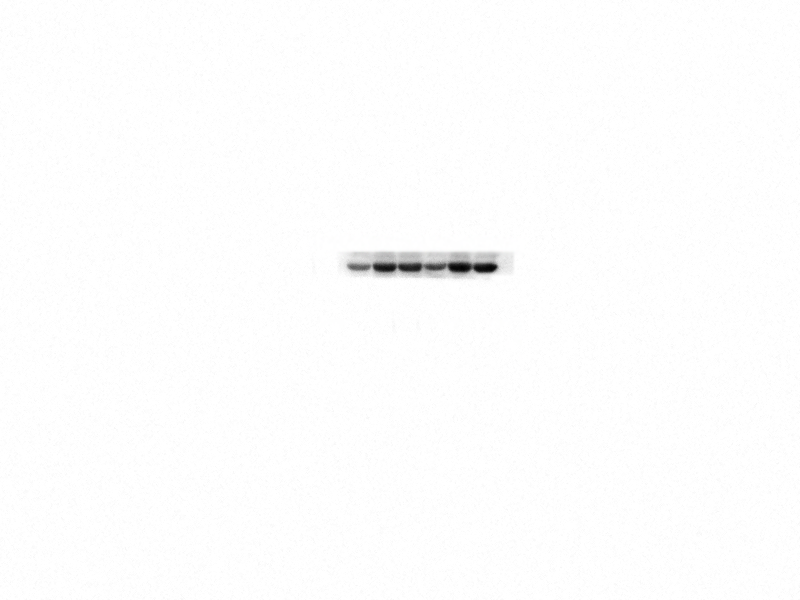

Supplement: Supplementary file 1 [file DataSheet1.zip › Suppl. Figs 1-7/4/CTGF.tif]

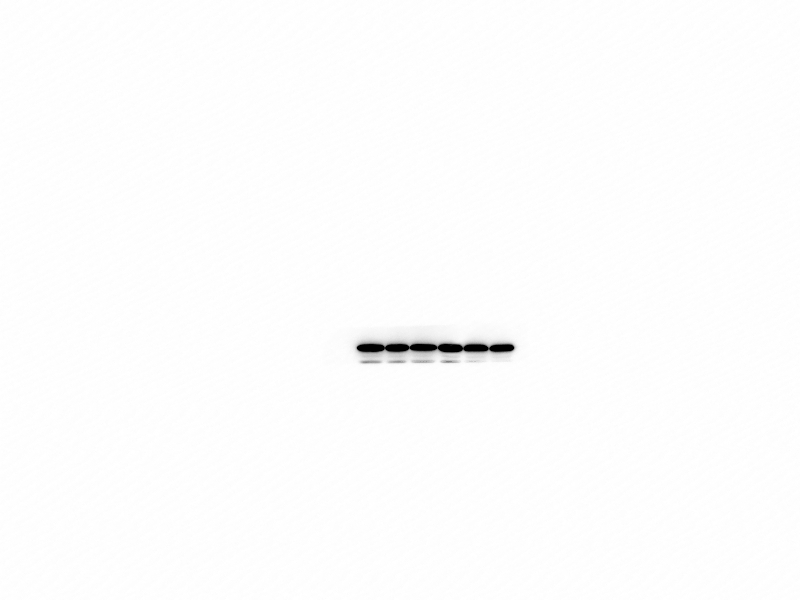

Supplement: Supplementary file 1 [file DataSheet1.zip › Suppl. Figs 1-7/4/╬▓-actin.tif]

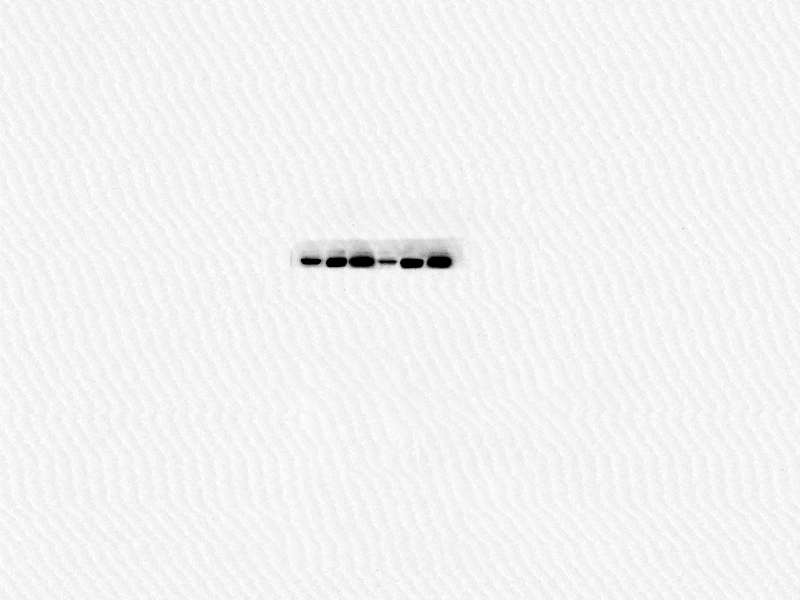

Supplement: Supplementary file 1 [file DataSheet1.zip › Suppl. Figs 1-7/4/Birc5.tif]

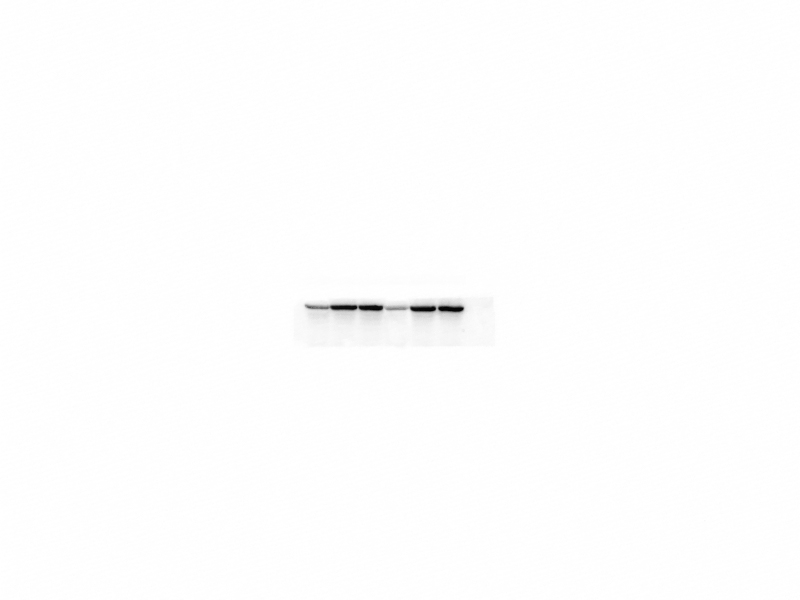

Supplement: Supplementary file 1 [file DataSheet1.zip › Suppl. Figs 1-7/3/MOB1.tif]

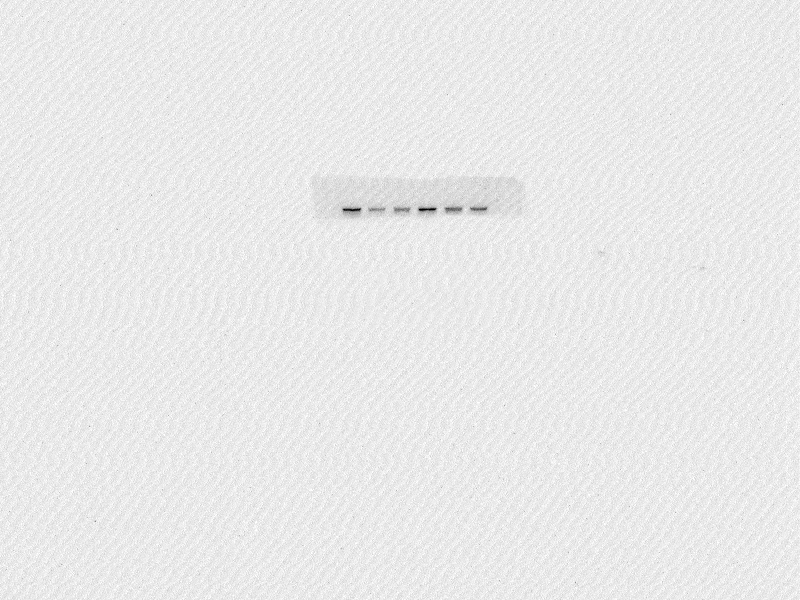

Supplement: Supplementary file 1 [file DataSheet1.zip › Suppl. Figs 1-7/3/p-MST1.tif]

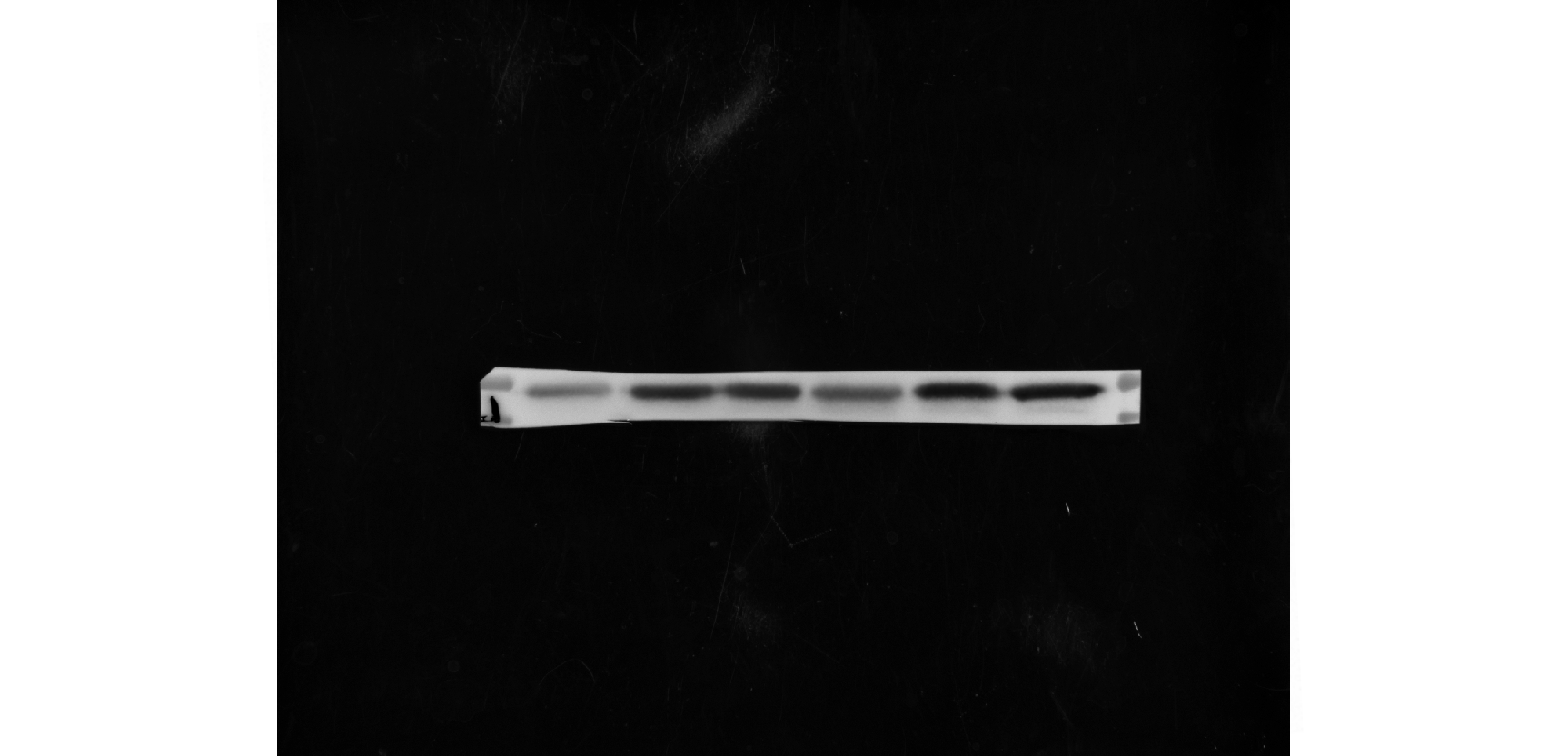

Supplement: Supplementary file 1 [file DataSheet1.zip › Suppl. Figs 1-7/3/p-YAP.tif]

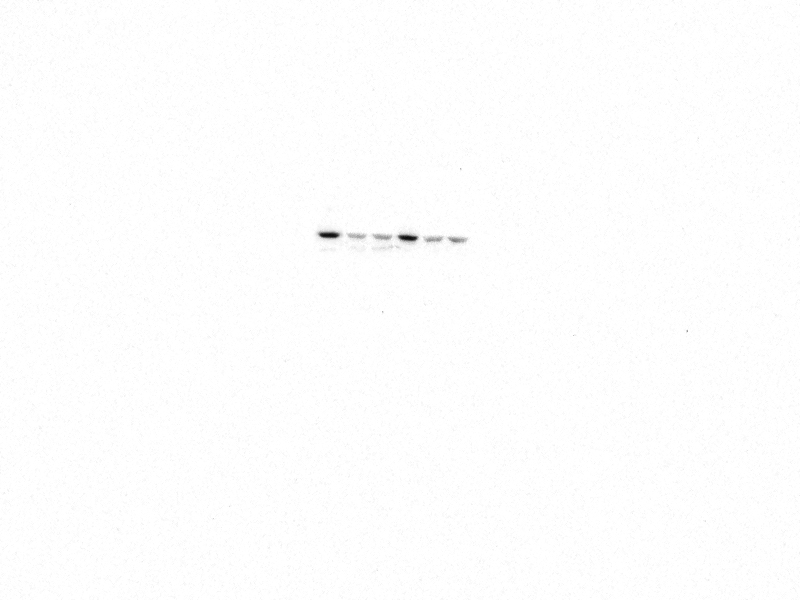

Supplement: Supplementary file 1 [file DataSheet1.zip › Suppl. Figs 1-7/3/p-MOB1.tif]

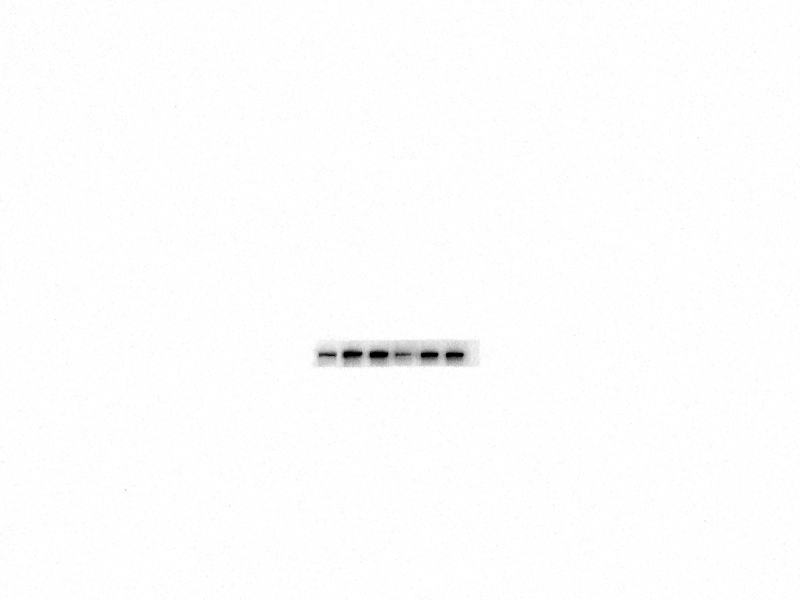

Supplement: Supplementary file 1 [file DataSheet1.zip › Suppl. Figs 1-7/3/MST1.tif]

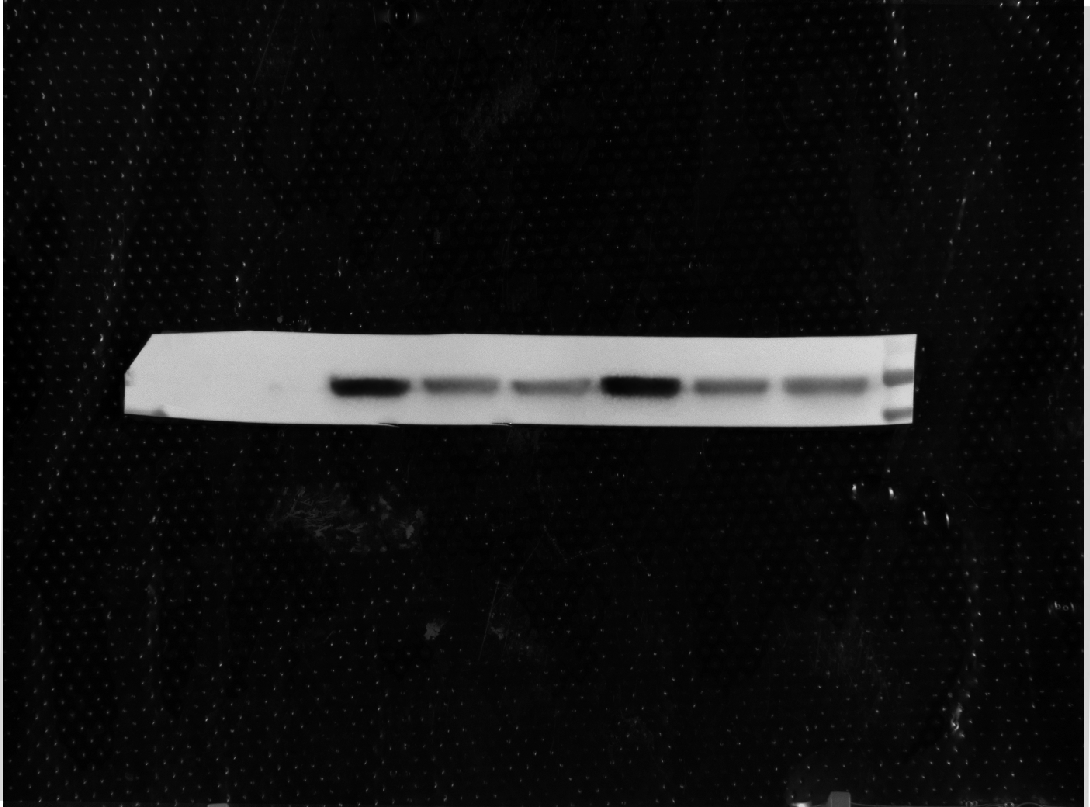

Supplement: Supplementary file 1 [file DataSheet1.zip › Suppl. Figs 1-7/3/YAP.tif]

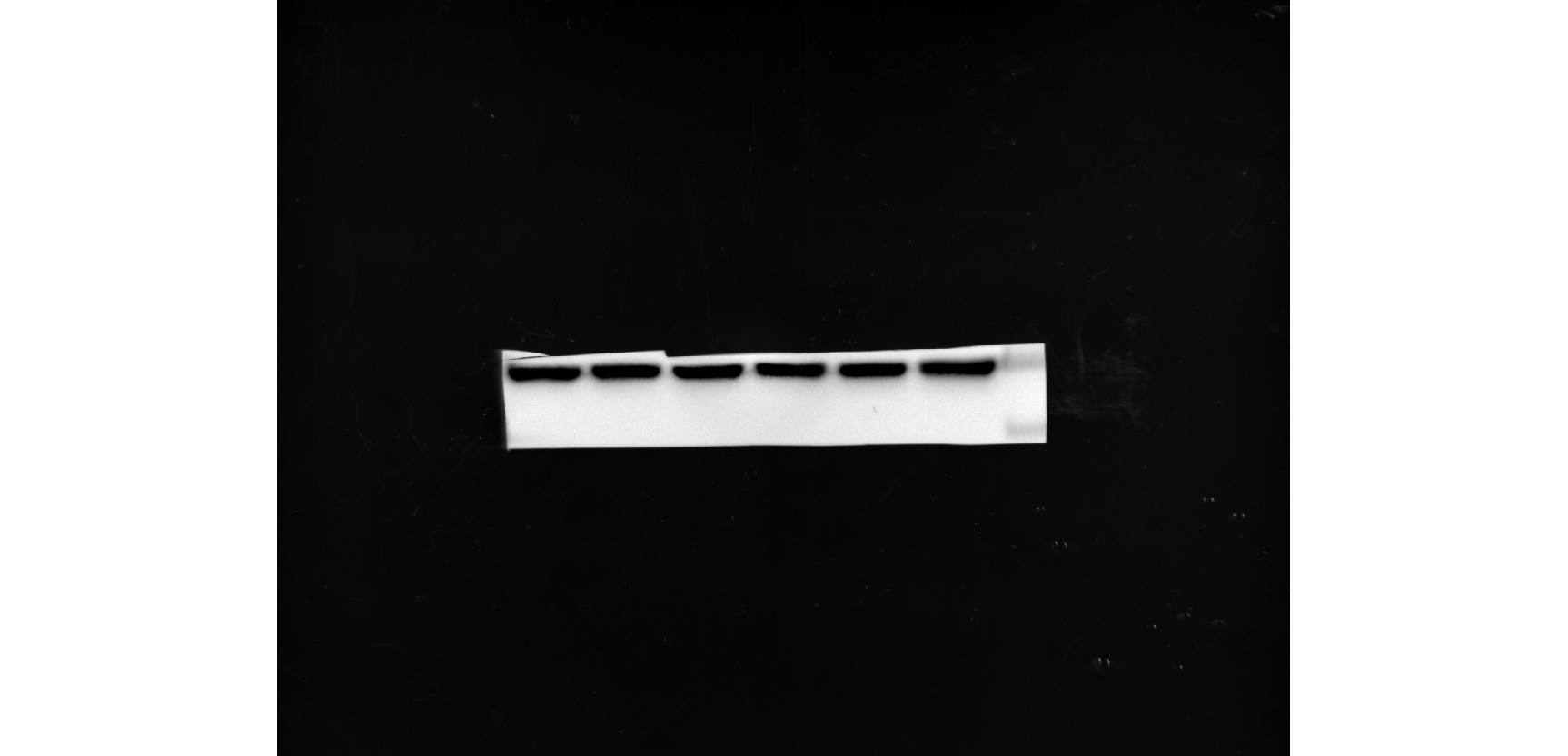

Supplement: Supplementary file 1 [file DataSheet1.zip › Suppl. Figs 1-7/3/╬▓-actin.tif]

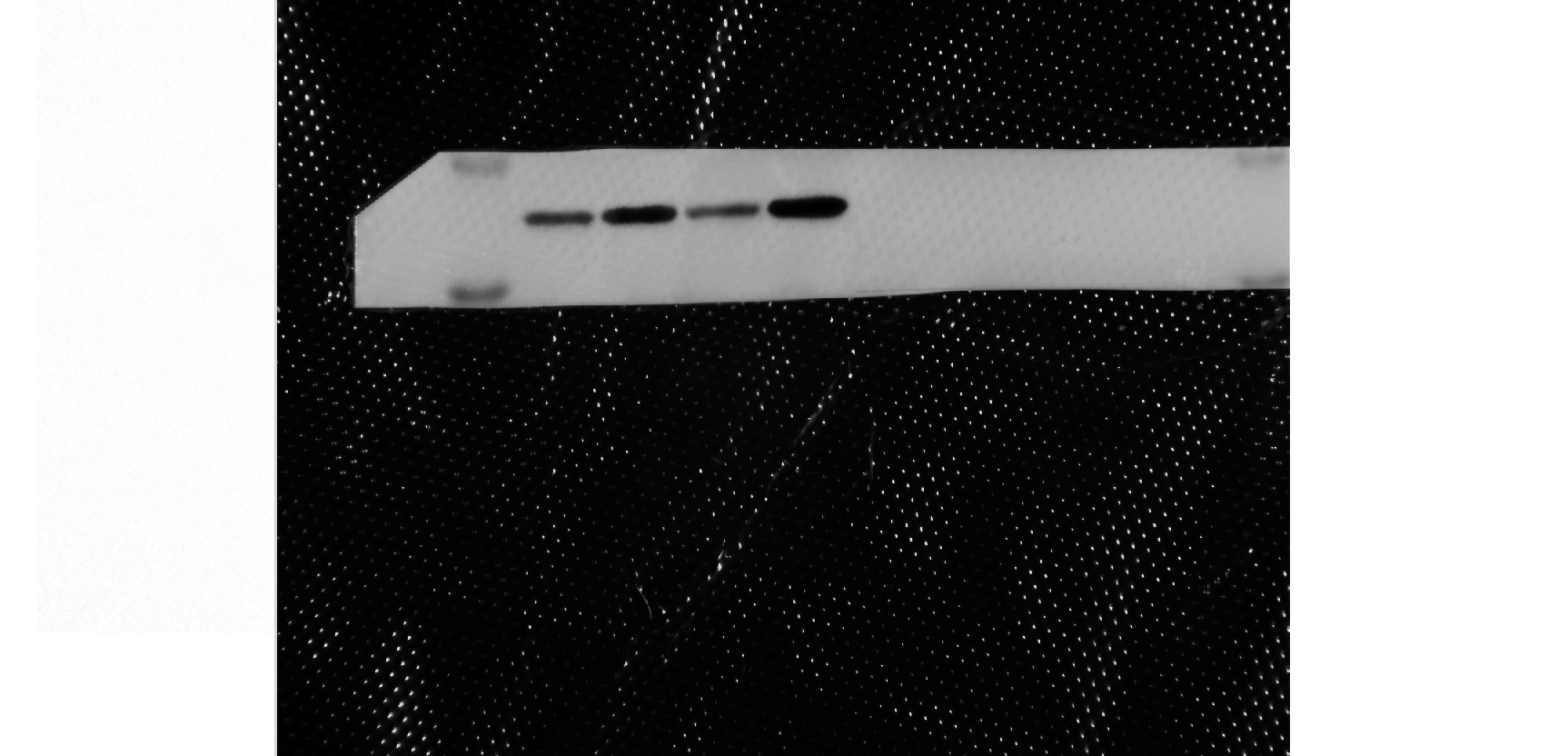

Supplement: Supplementary file 1 [file DataSheet1.zip › Suppl. Figs 1-7/2/E-cadherin.tif]

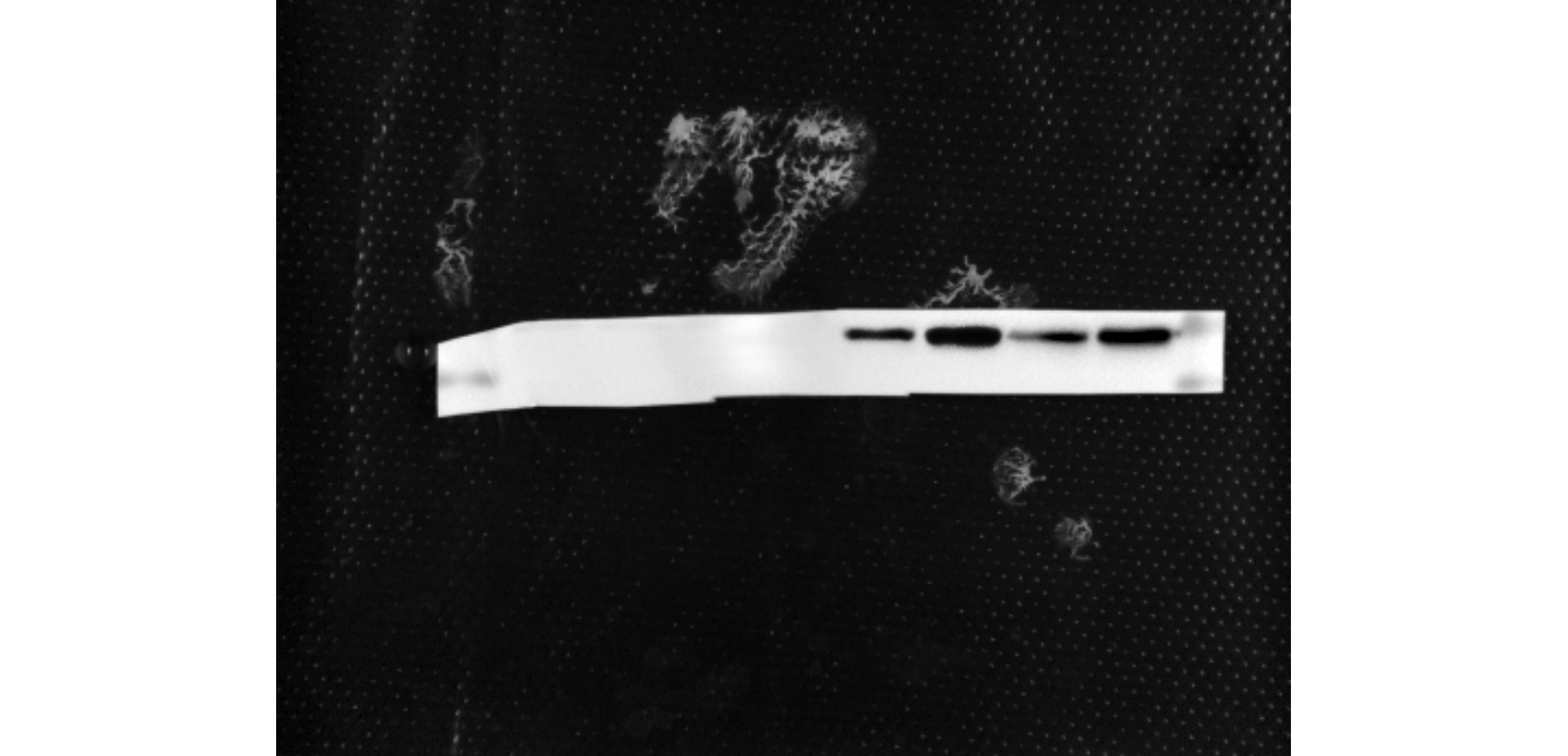

Supplement: Supplementary file 1 [file DataSheet1.zip › Suppl. Figs 1-7/2/cleaved caspase-3 2.tif]

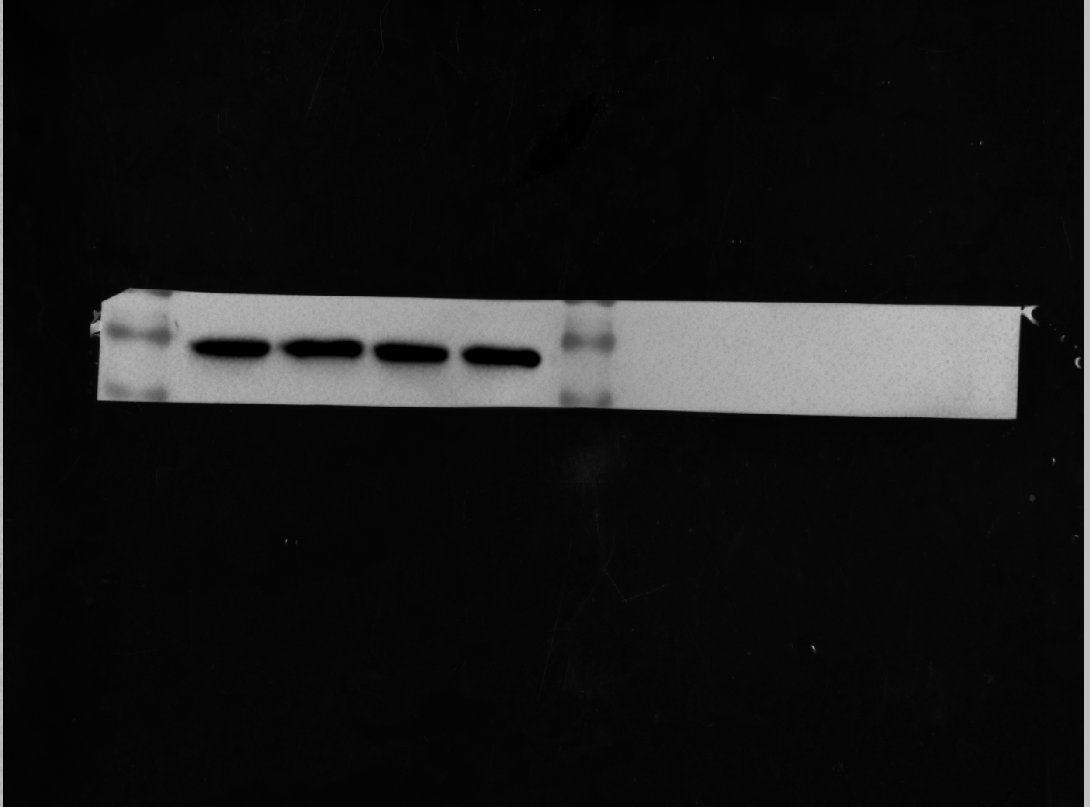

Supplement: Supplementary file 1 [file DataSheet1.zip › Suppl. Figs 1-7/2/╬▓-actin.tif]

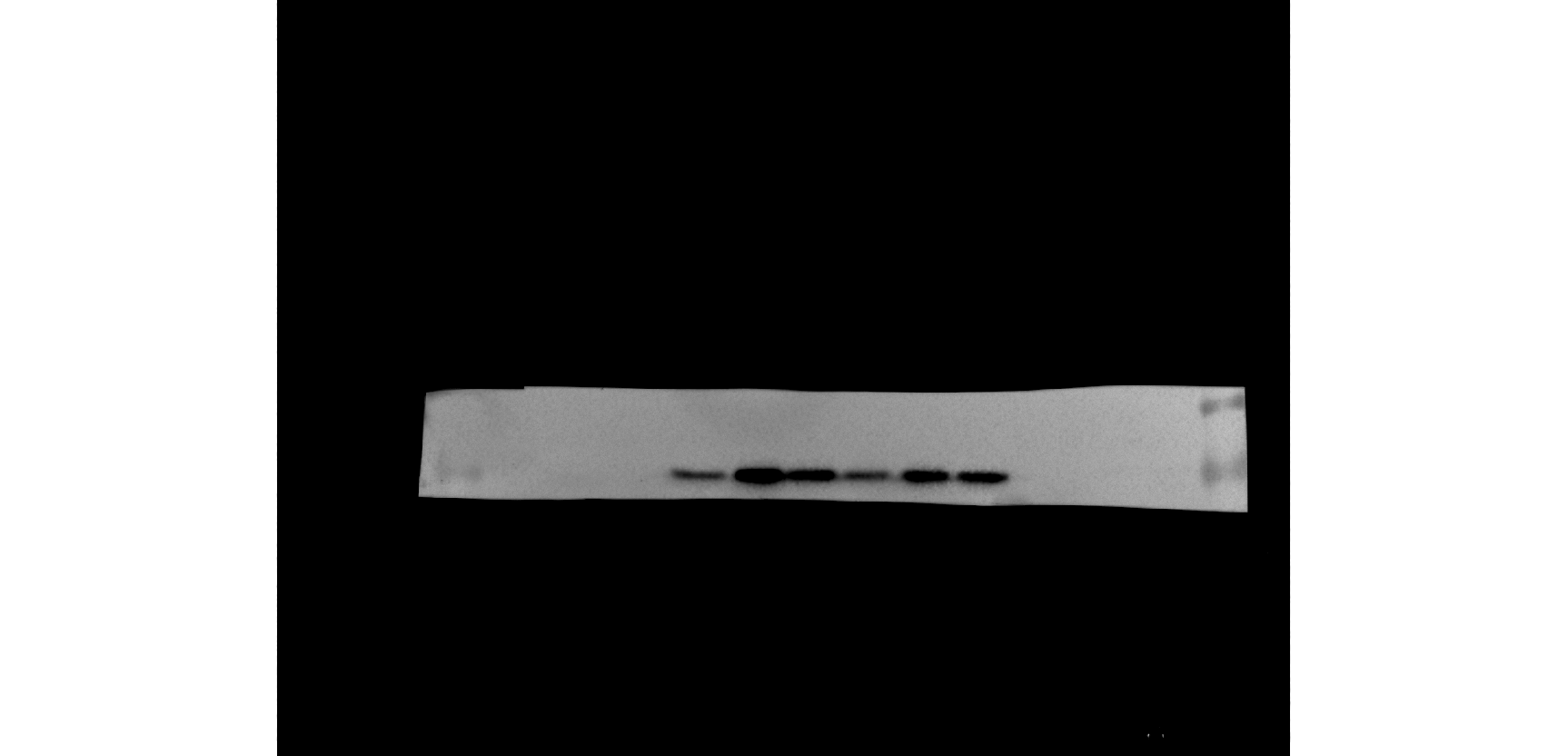

Supplement: Supplementary file 1 [file DataSheet1.zip › Suppl. Figs 1-7/2/Ki67.tif]

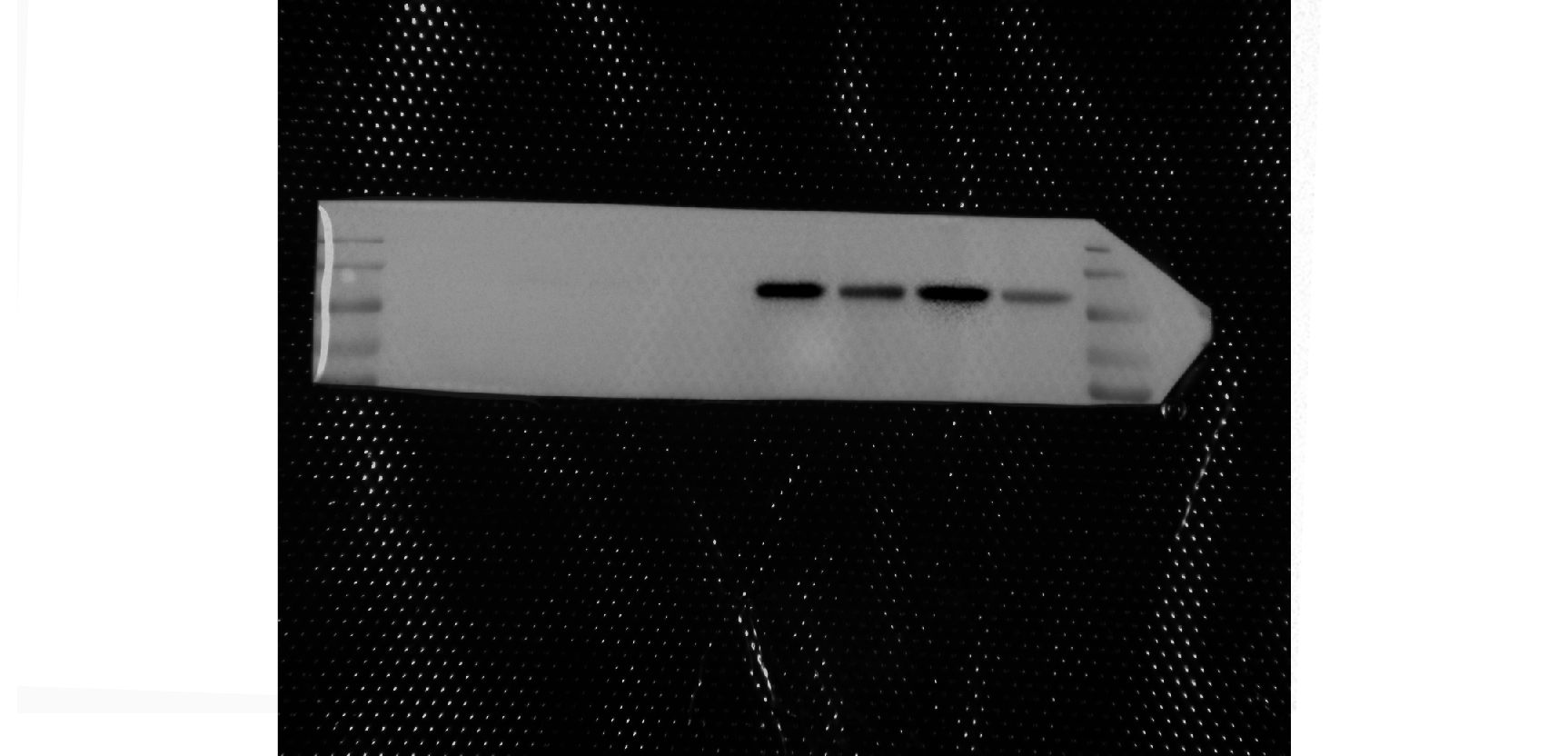

Supplement: Supplementary file 1 [file DataSheet1.zip › Suppl. Figs 1-7/2/Ki67 2.tif]

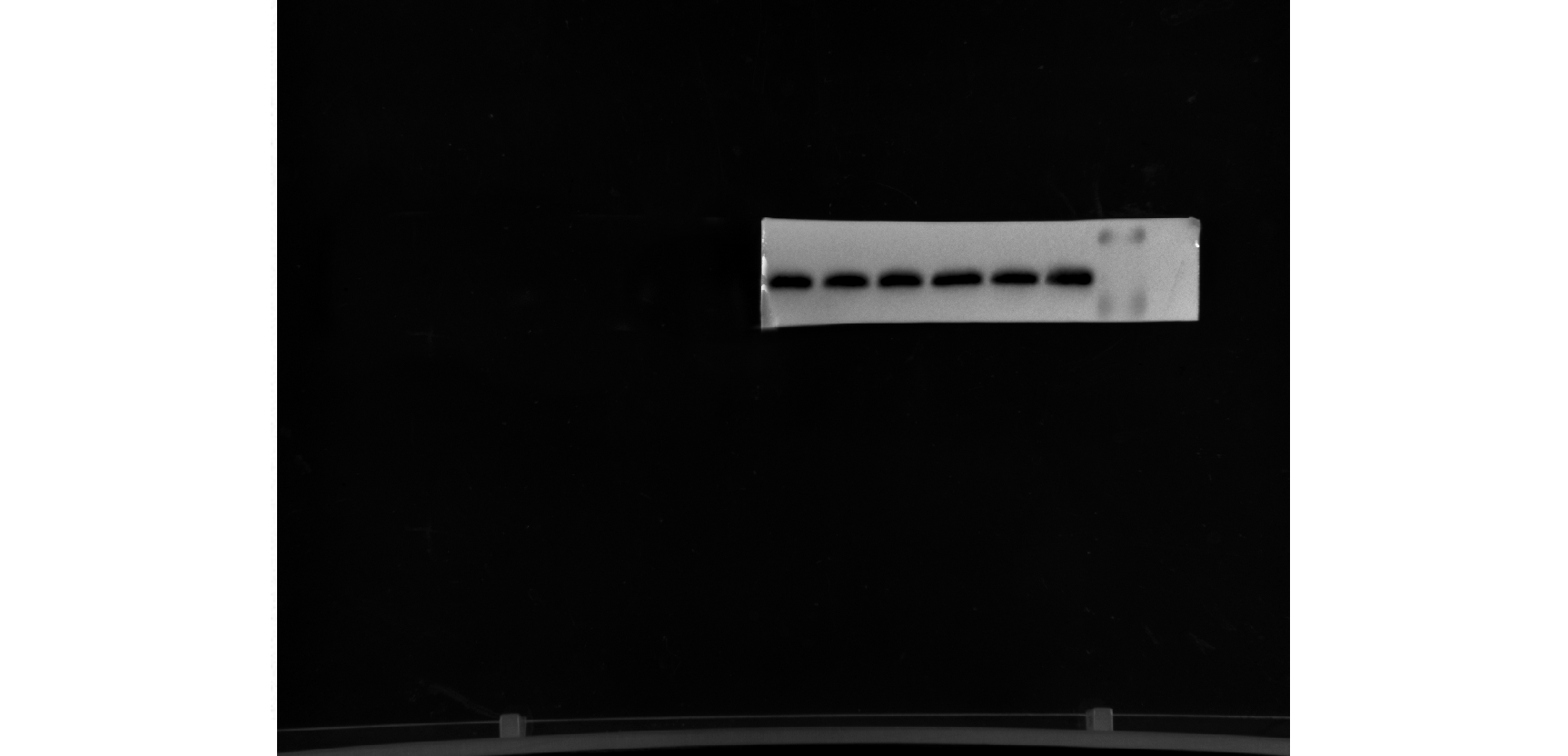

Supplement: Supplementary file 1 [file DataSheet1.zip › Suppl. Figs 1-7/2/╬▓-actin !.tif]

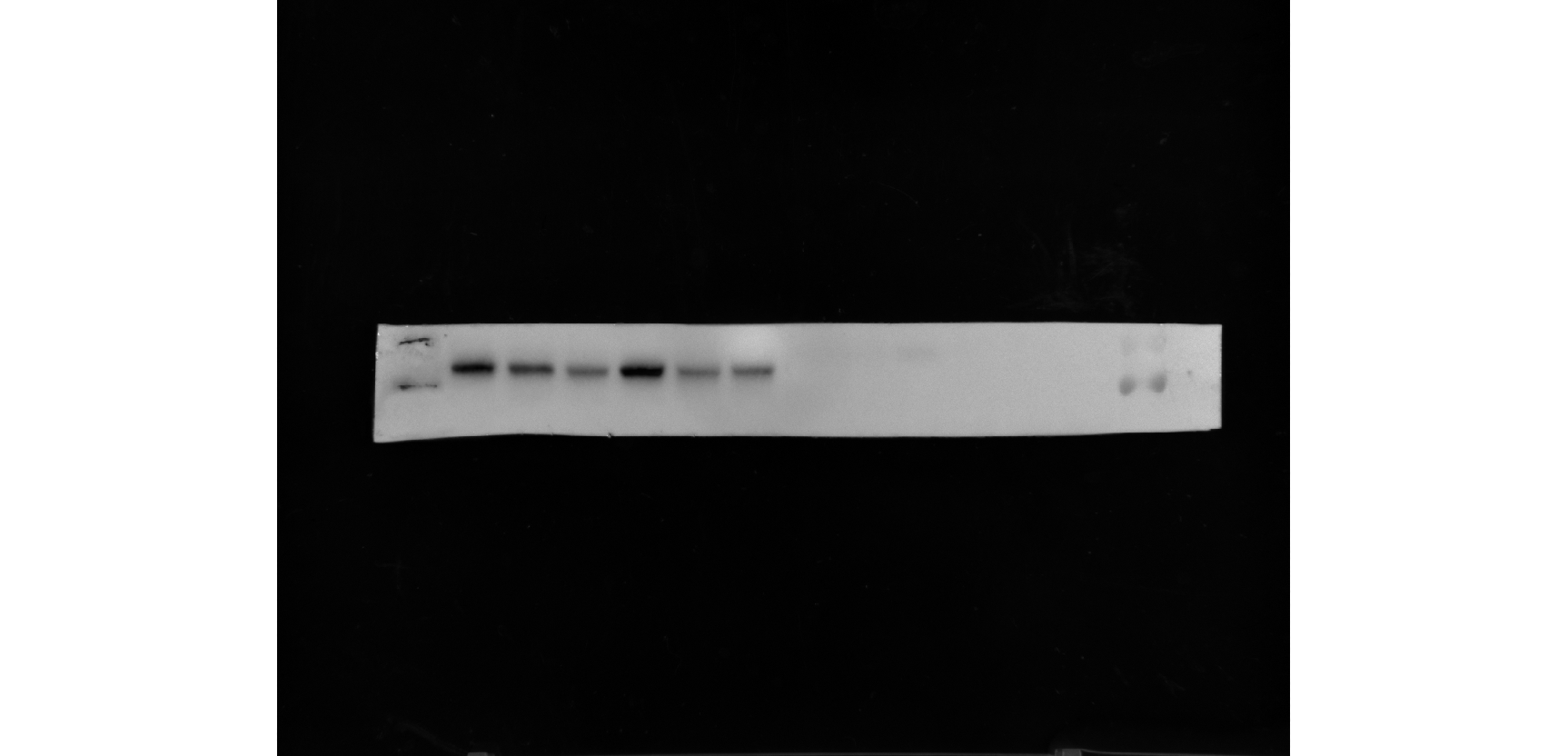

Supplement: Supplementary file 1 [file DataSheet1.zip › Suppl. Figs 1-7/2/E-cadherin !.tif]

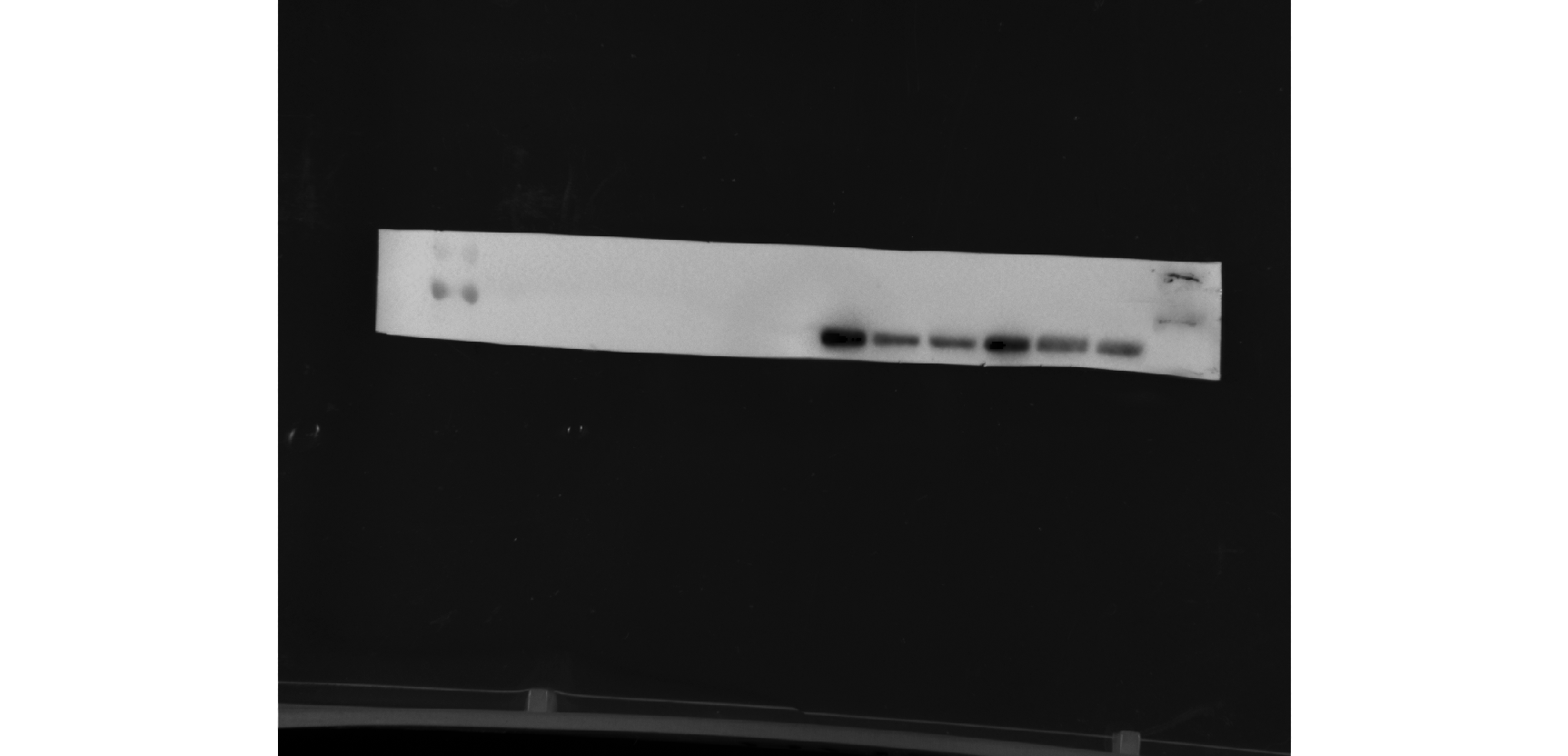

Supplement: Supplementary file 1 [file DataSheet1.zip › Suppl. Figs 1-7/2/cleaved caspase-3.tif]

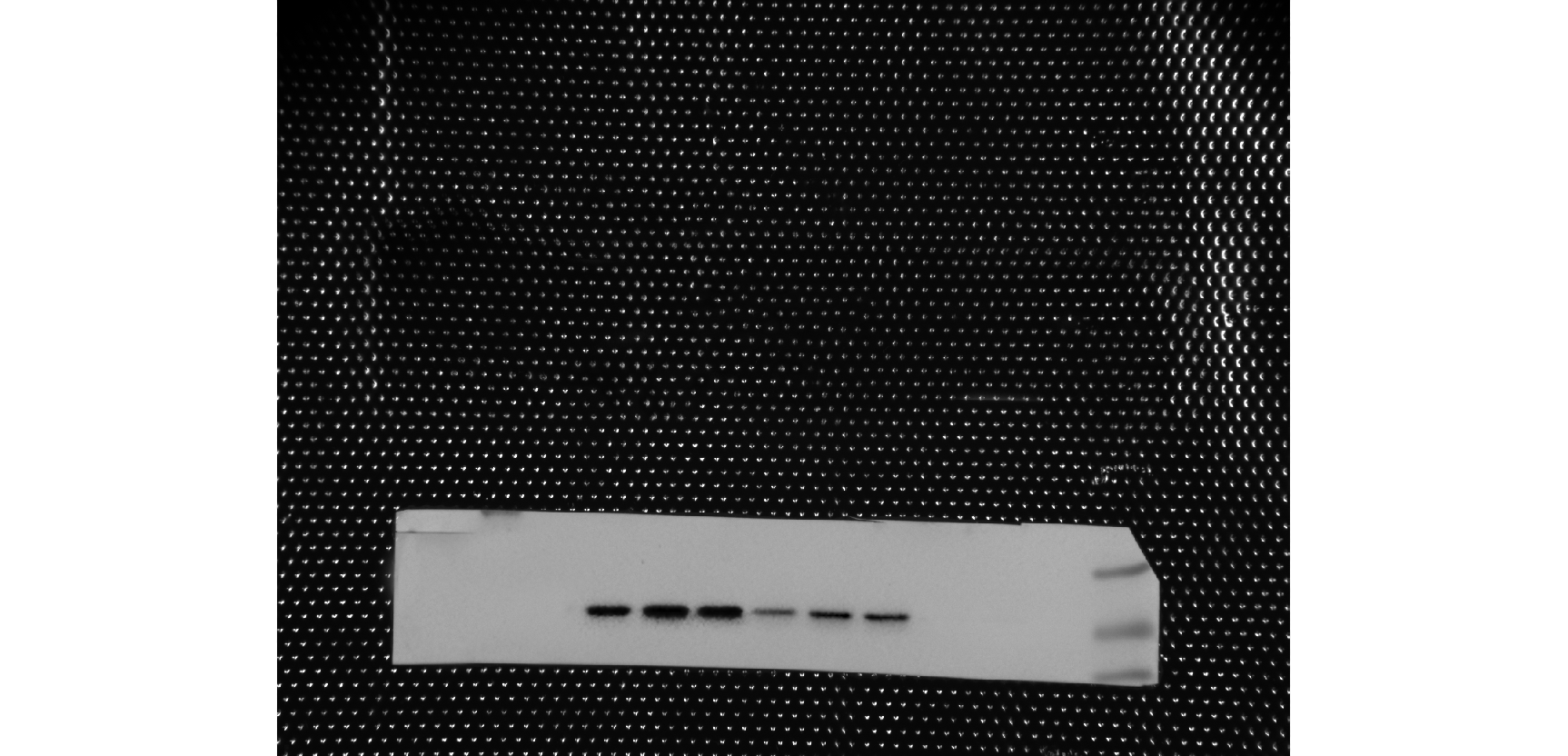

Supplement: Supplementary file 1 [file DataSheet1.zip › Suppl. Figs 1-7/5/YAP 2.tif]

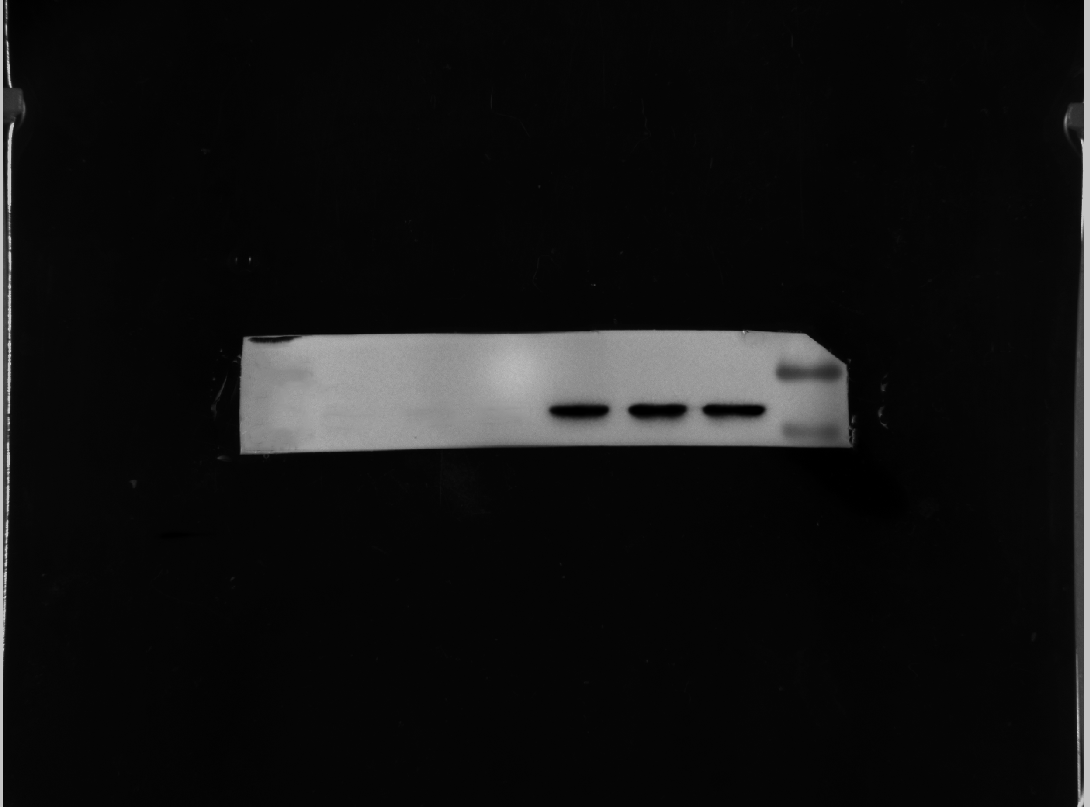

Supplement: Supplementary file 1 [file DataSheet1.zip › Suppl. Figs 1-7/5/╬▓-actin#1.tif]

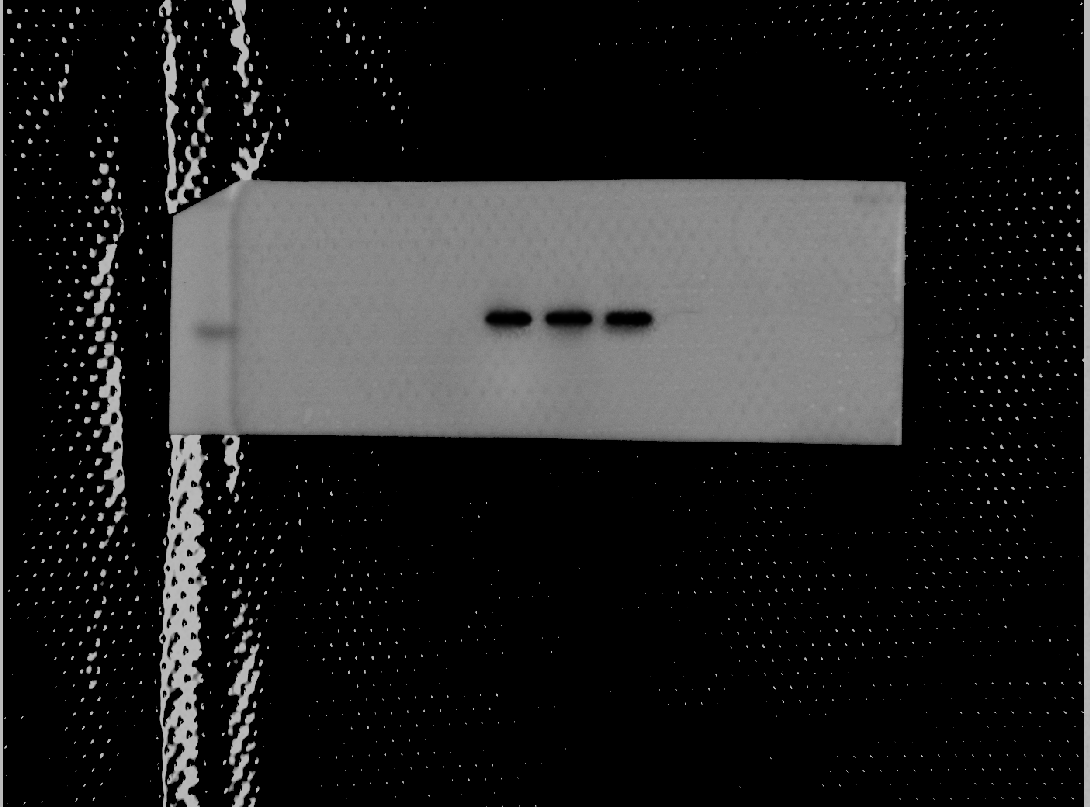

Supplement: Supplementary file 1 [file DataSheet1.zip › Suppl. Figs 1-7/5/╬▓-actin#2.tif]

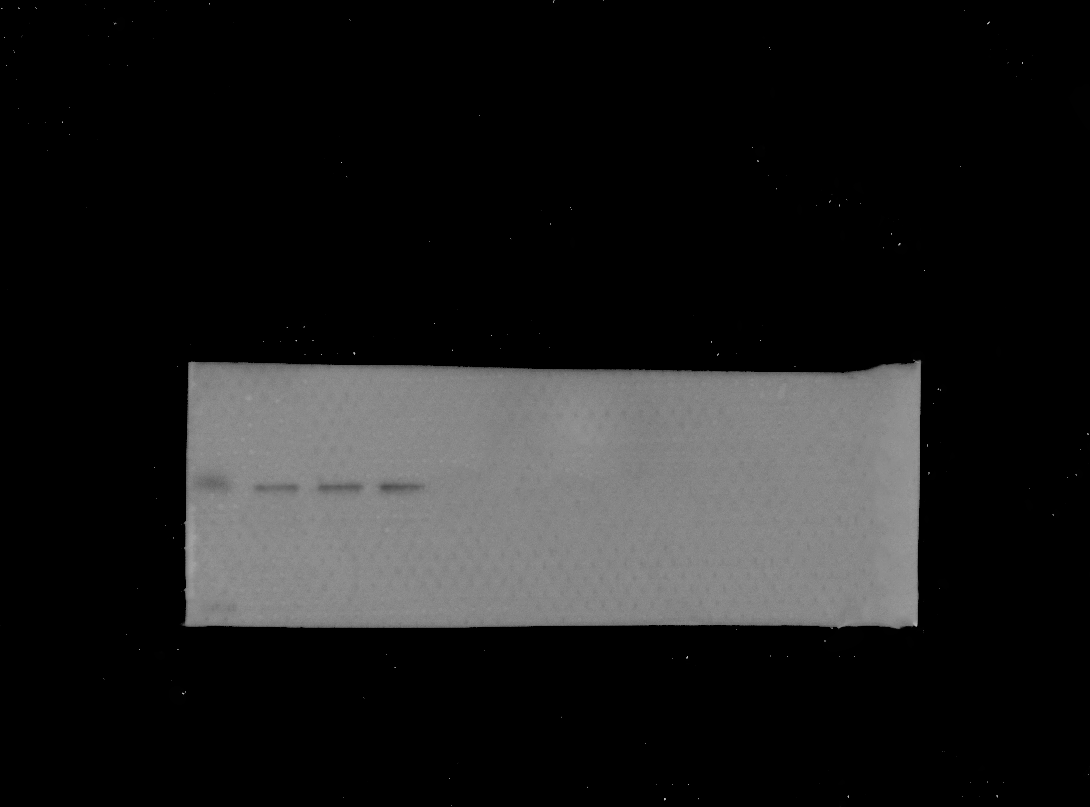

Supplement: Supplementary file 1 [file DataSheet1.zip › Suppl. Figs 1-7/5/LaminB#2.tif]

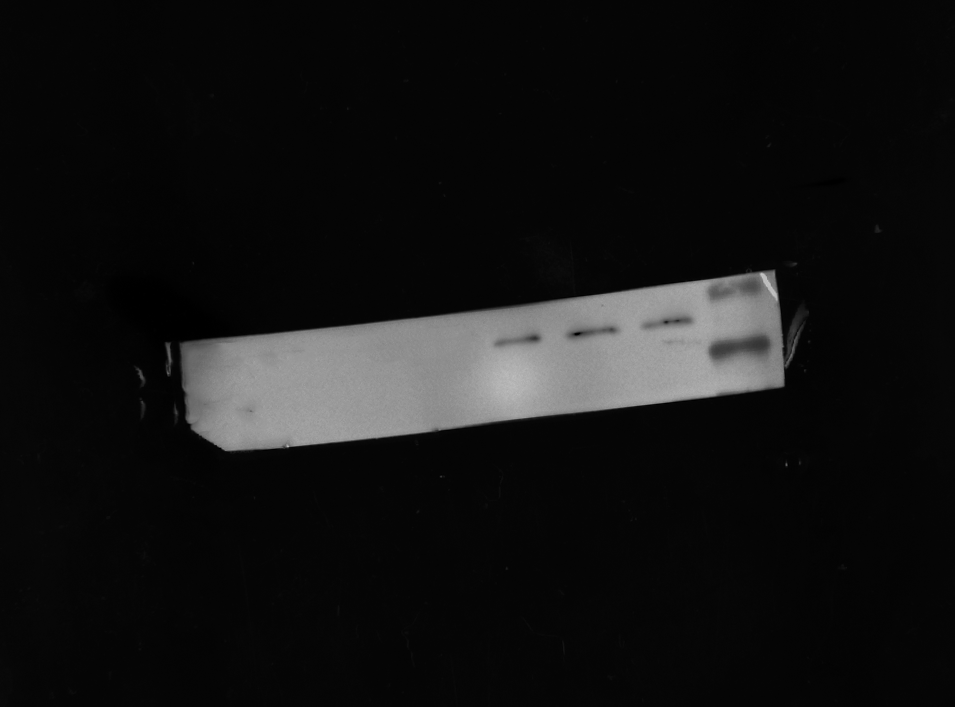

Supplement: Supplementary file 1 [file DataSheet1.zip › Suppl. Figs 1-7/5/LaminB#1.tif]

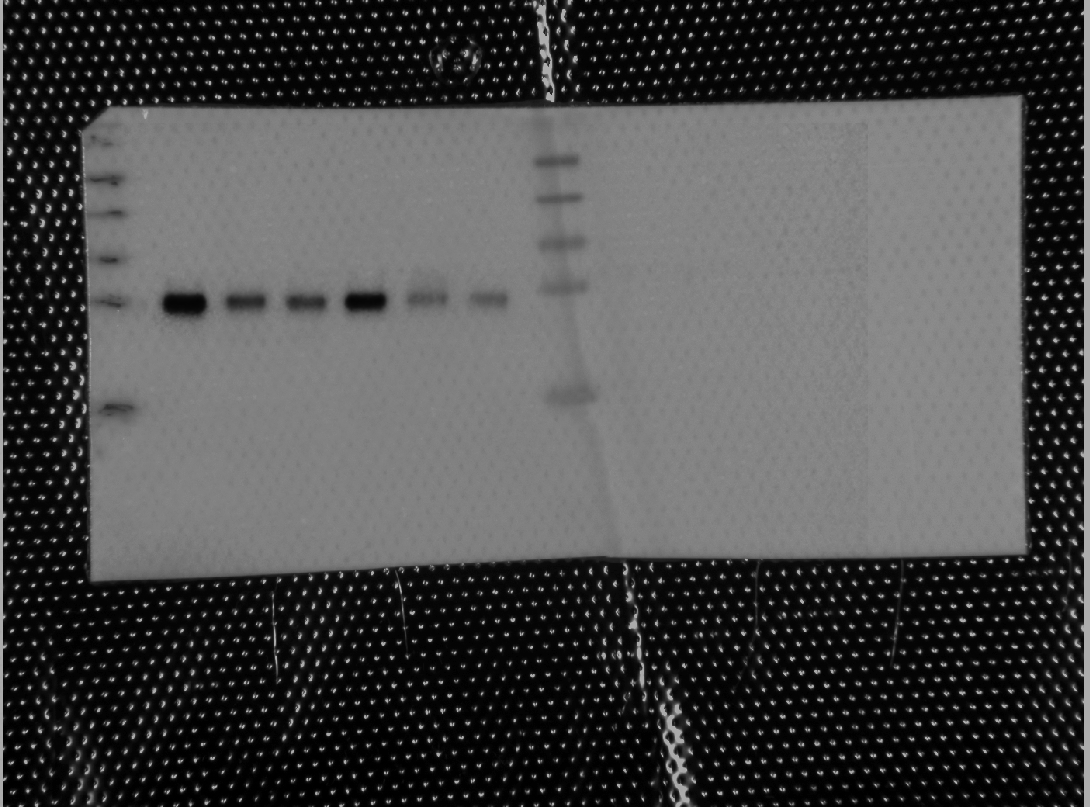

Supplement: Supplementary file 1 [file DataSheet1.zip › Suppl. Figs 1-7/5/YAP.tif]

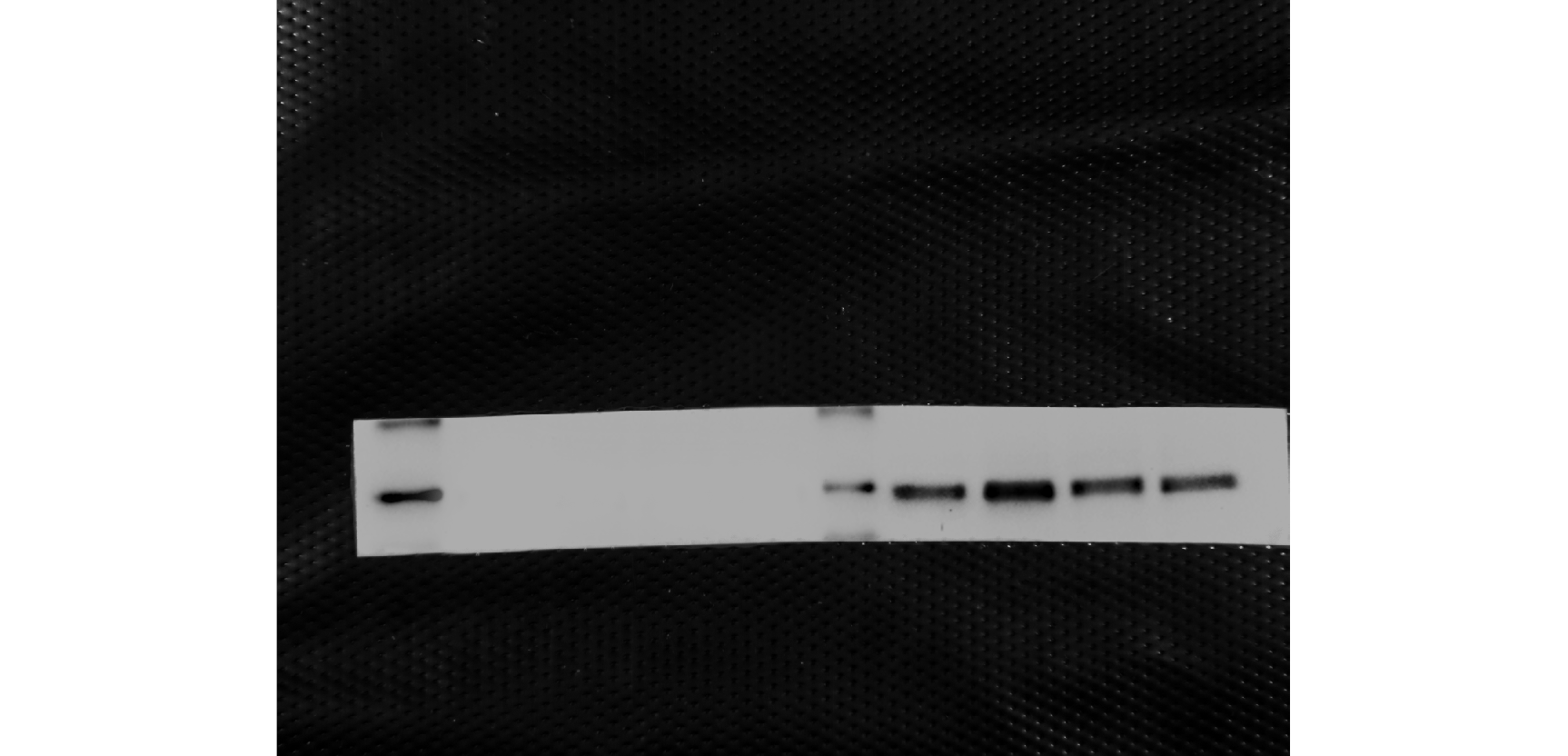

Supplement: Supplementary file 2 [file DataSheet2.zip › Suppl. Figs 8-13/9/E-cadherin.tif]

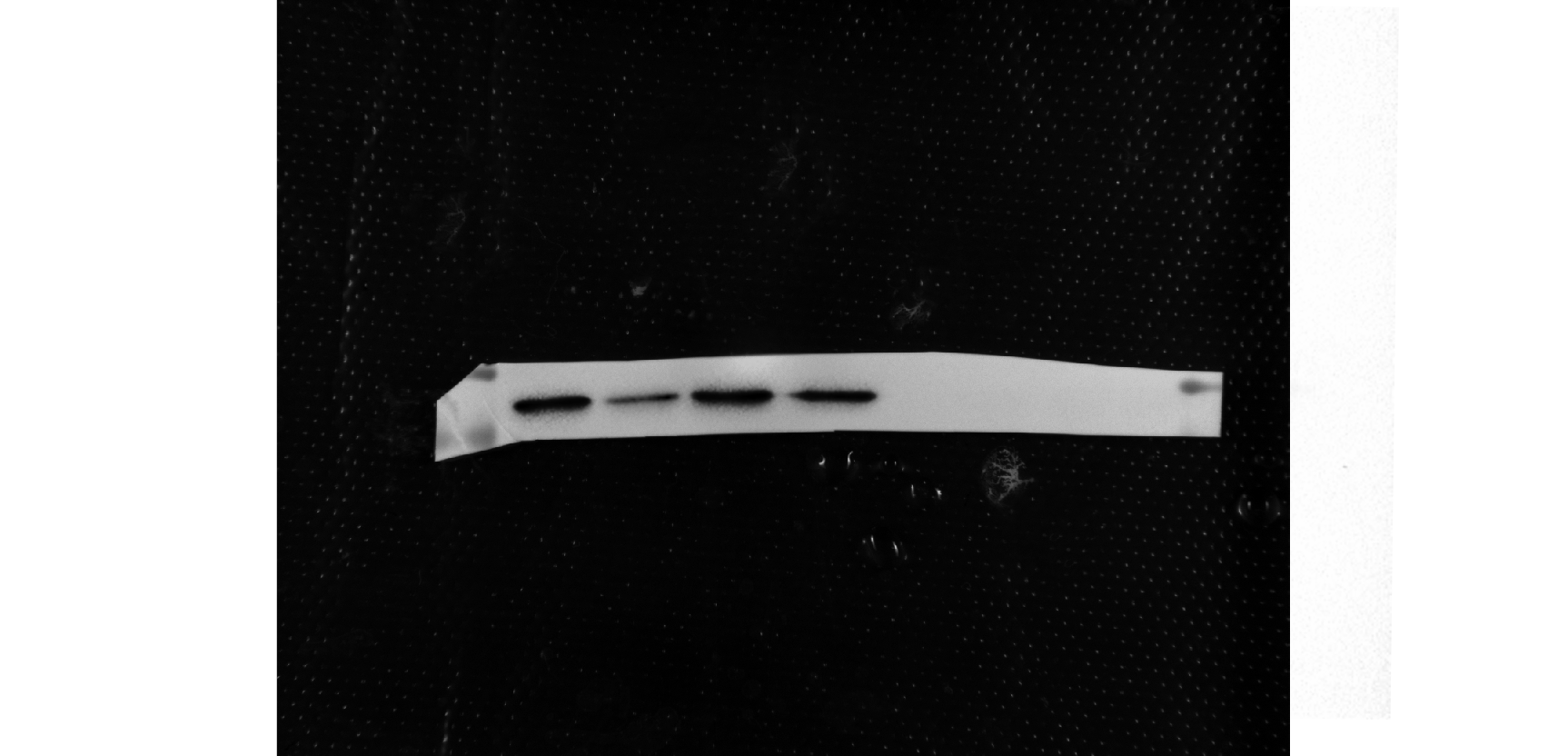

Supplement: Supplementary file 2 [file DataSheet2.zip › Suppl. Figs 8-13/9/Ki67.tif]

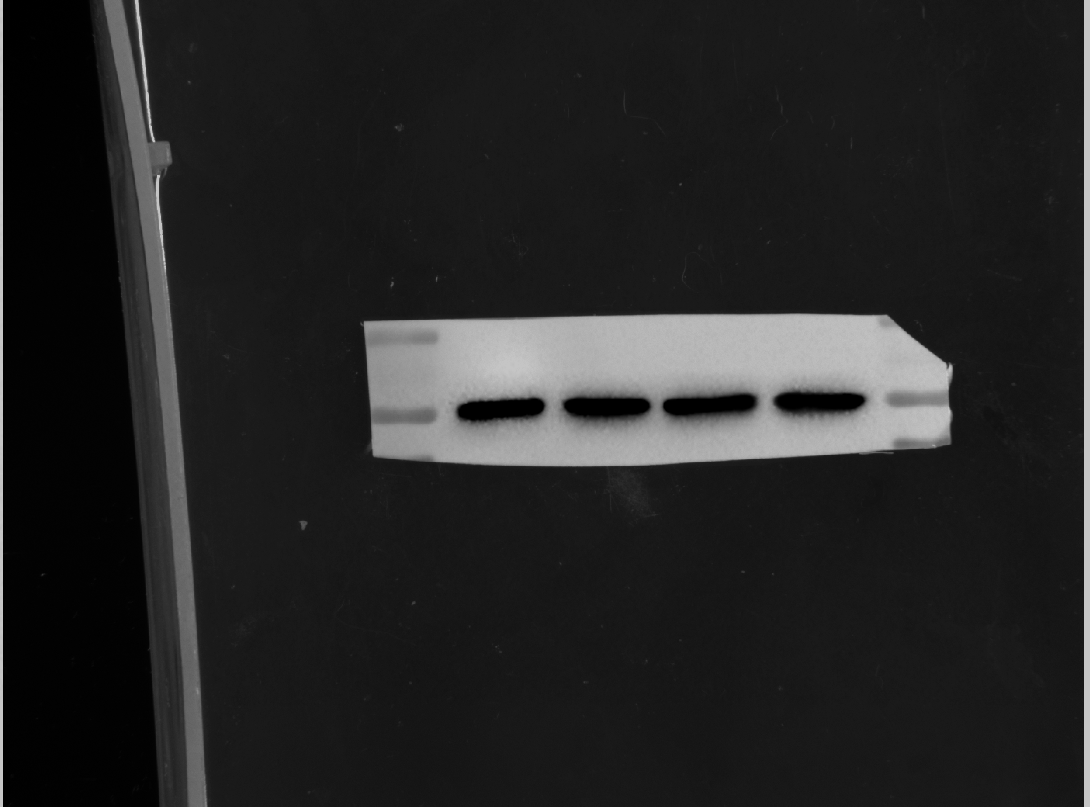

Supplement: Supplementary file 2 [file DataSheet2.zip › Suppl. Figs 8-13/9/╬▓-actin 6.tif]

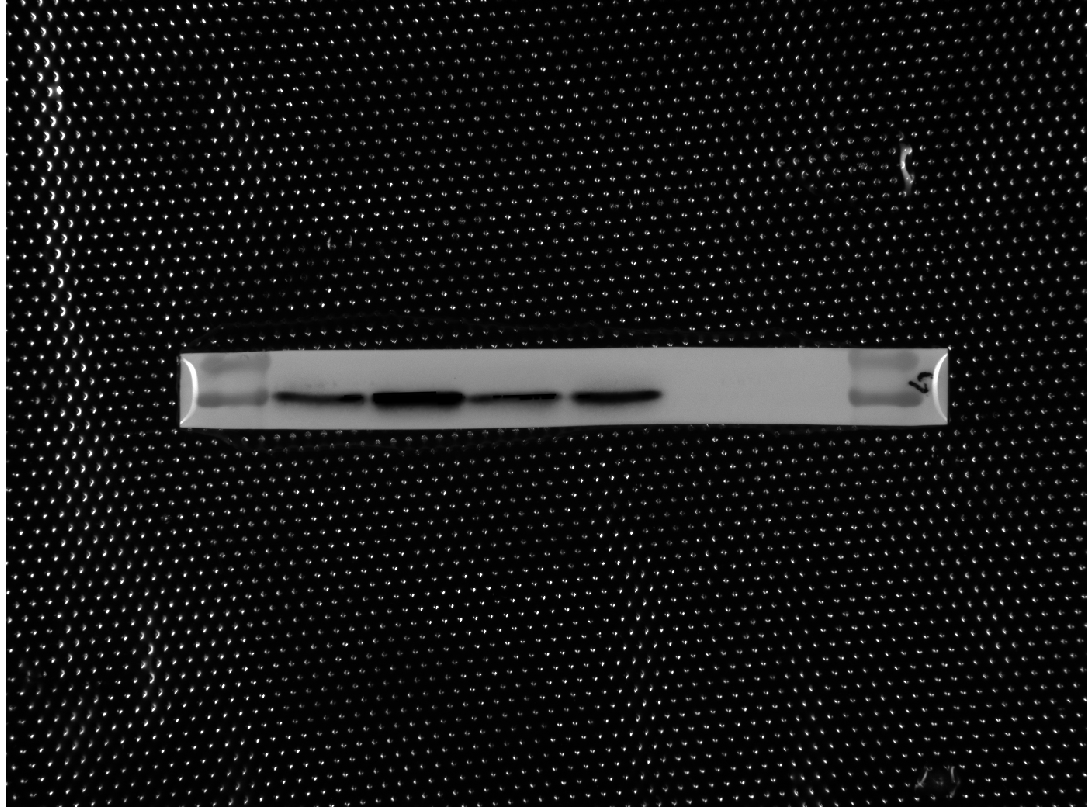

Supplement: Supplementary file 2 [file DataSheet2.zip › Suppl. Figs 8-13/9/cleaved caspase-3.tif]

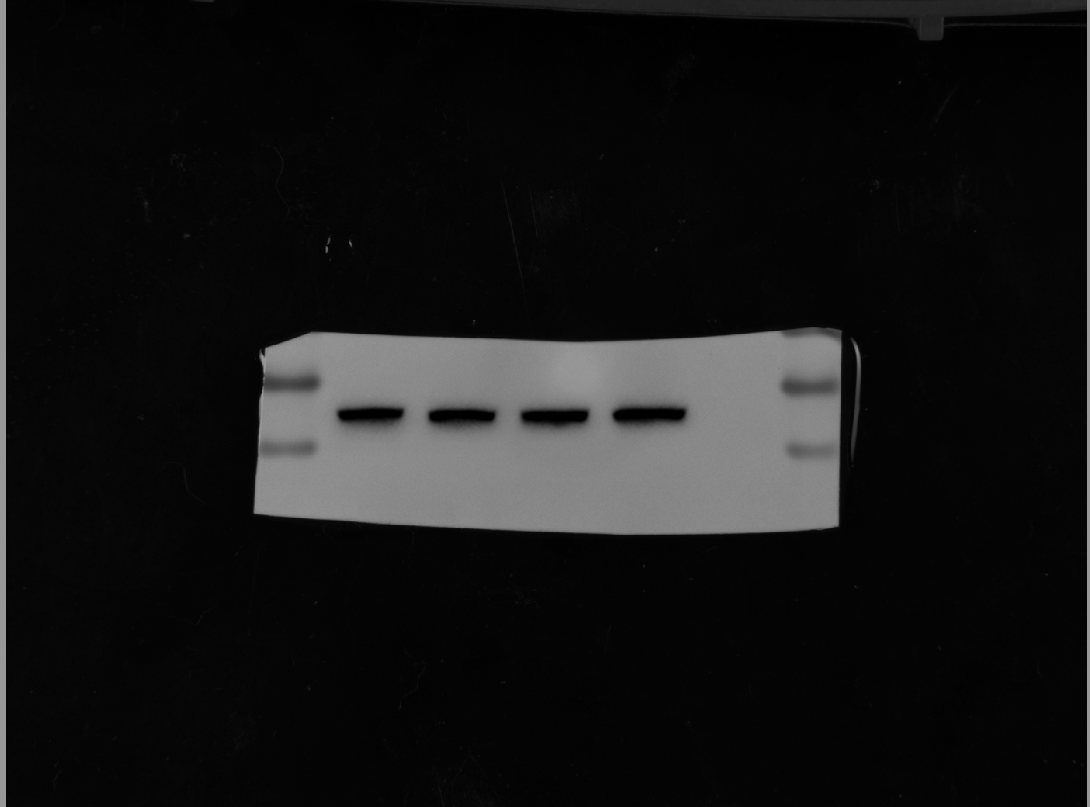

Supplement: Supplementary file 2 [file DataSheet2.zip › Suppl. Figs 8-13/11/╬▓-actin 8.tif]

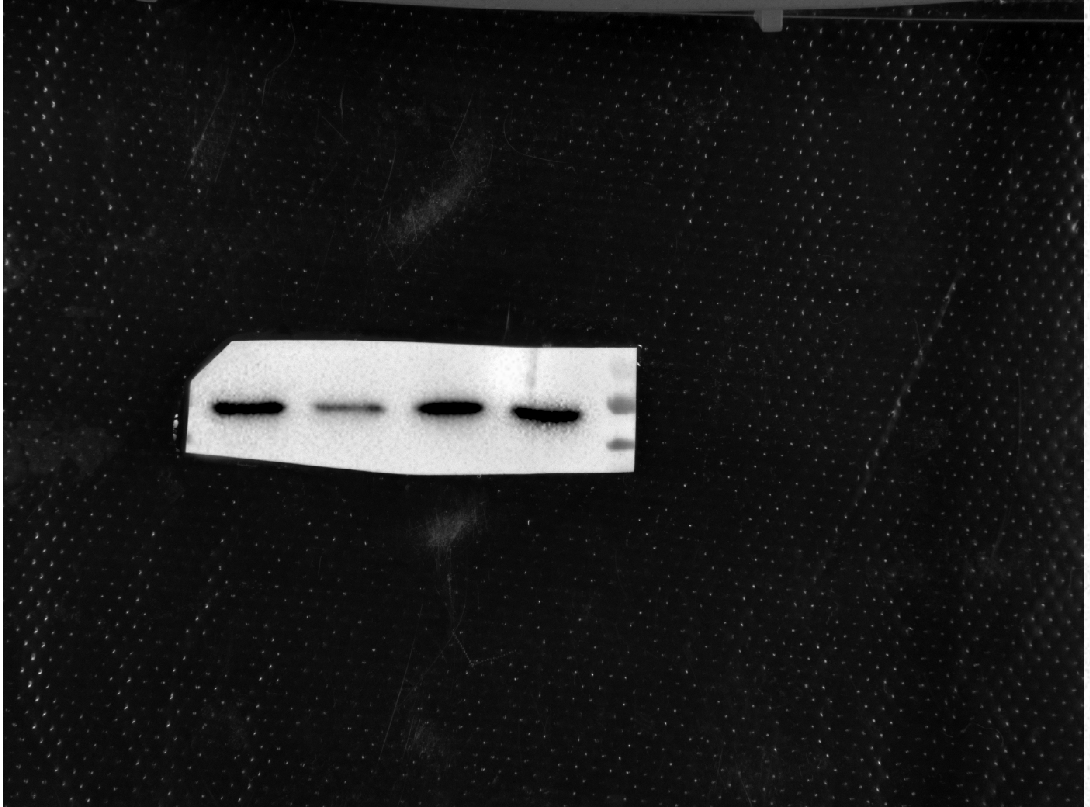

Supplement: Supplementary file 2 [file DataSheet2.zip › Suppl. Figs 8-13/11/Cyr61.tif]

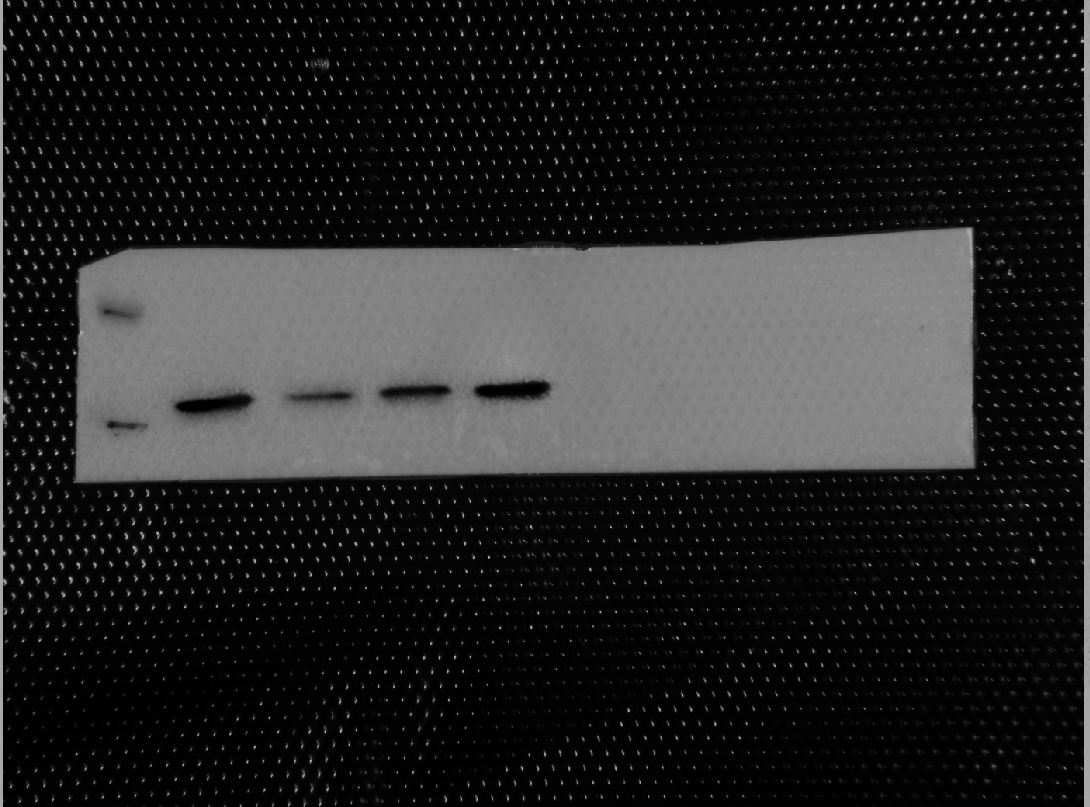

Supplement: Supplementary file 2 [file DataSheet2.zip › Suppl. Figs 8-13/11/CTGF.tif]

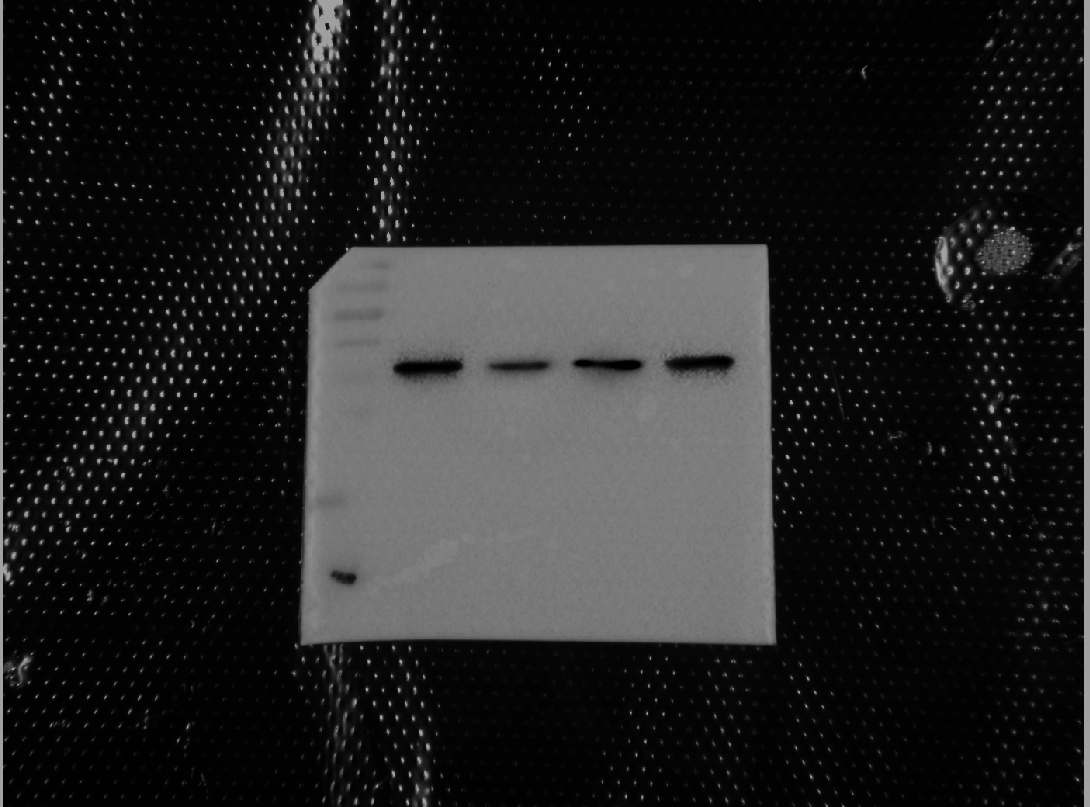

Supplement: Supplementary file 2 [file DataSheet2.zip › Suppl. Figs 8-13/11/Birc5.tif]

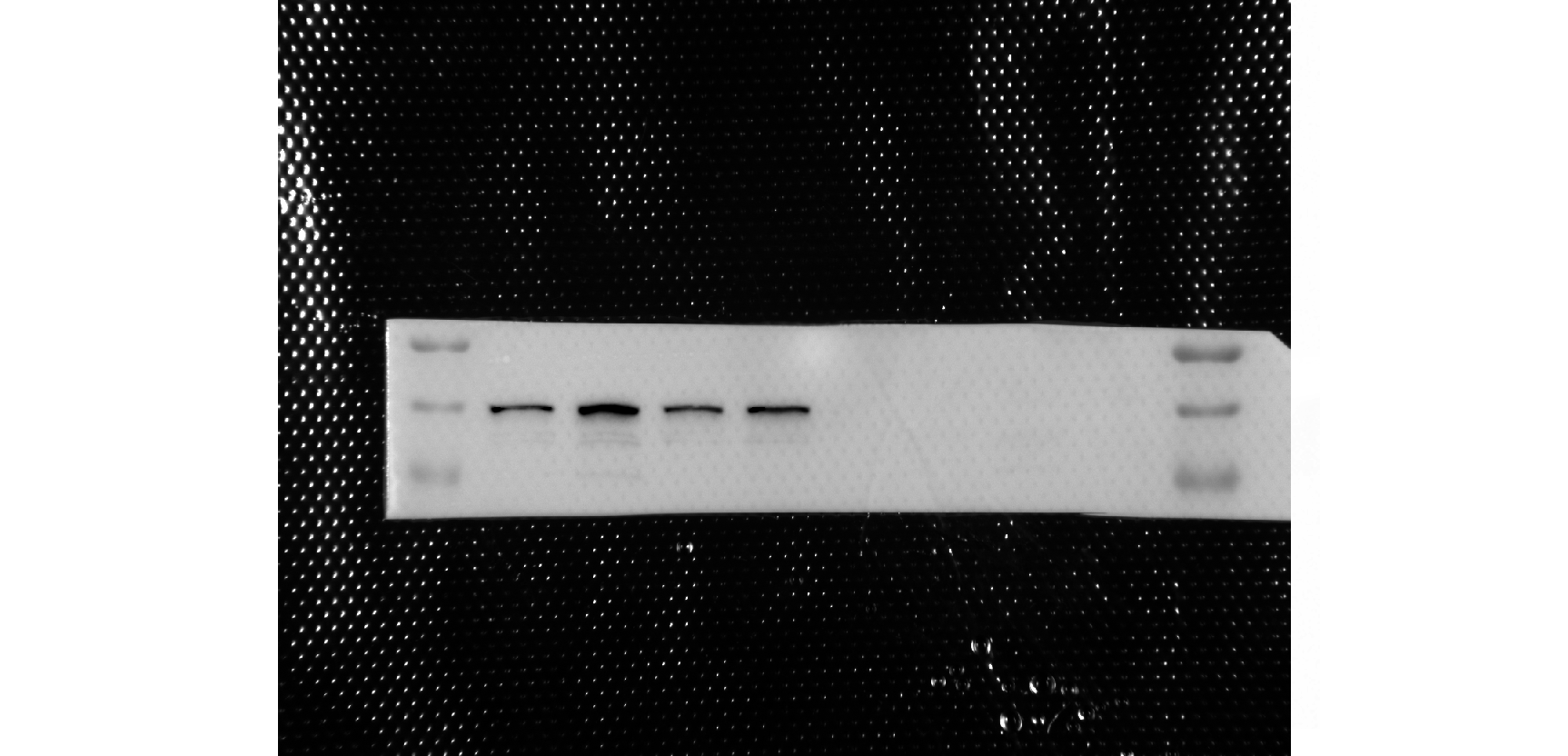

Supplement: Supplementary file 2 [file DataSheet2.zip › Suppl. Figs 8-13/10/cleaved caspase-3 2.tif]

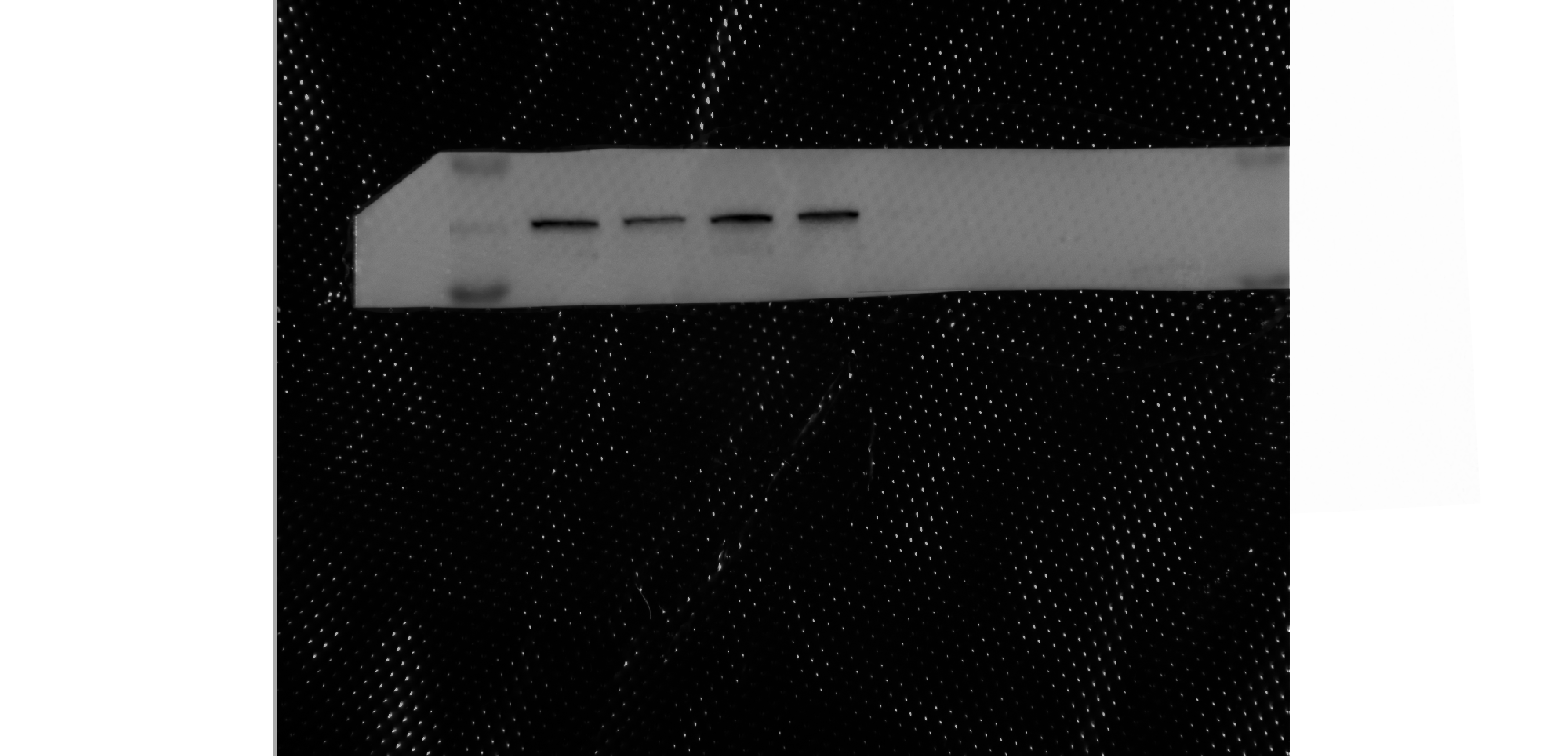

Supplement: Supplementary file 2 [file DataSheet2.zip › Suppl. Figs 8-13/10/Ki67 2.tif]

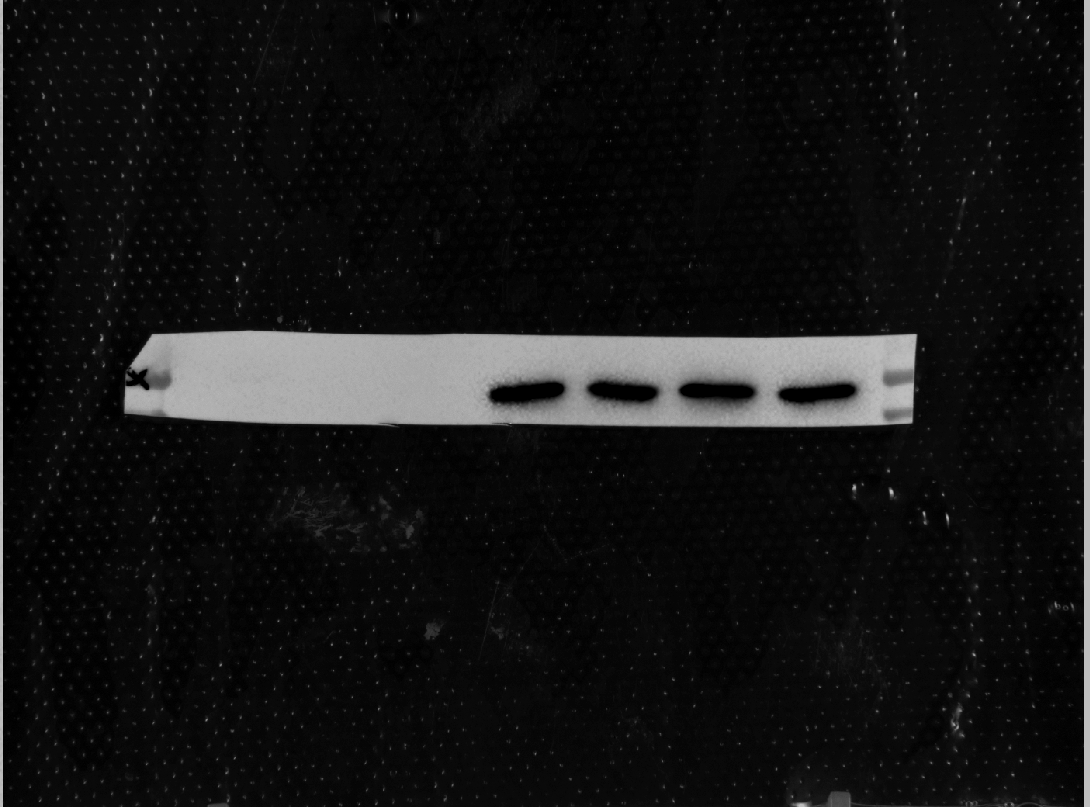

Supplement: Supplementary file 2 [file DataSheet2.zip › Suppl. Figs 8-13/10/╬▓-actin 7.tif]

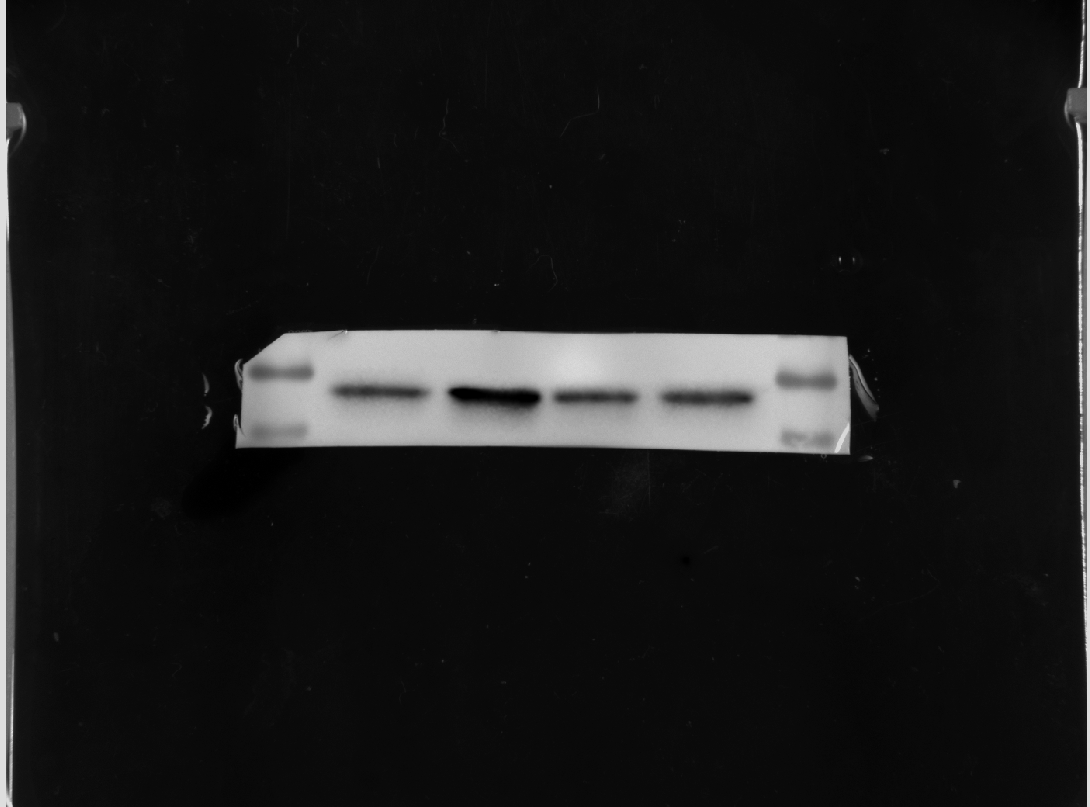

Supplement: Supplementary file 2 [file DataSheet2.zip › Suppl. Figs 8-13/10/E-cadherin 2.tif]

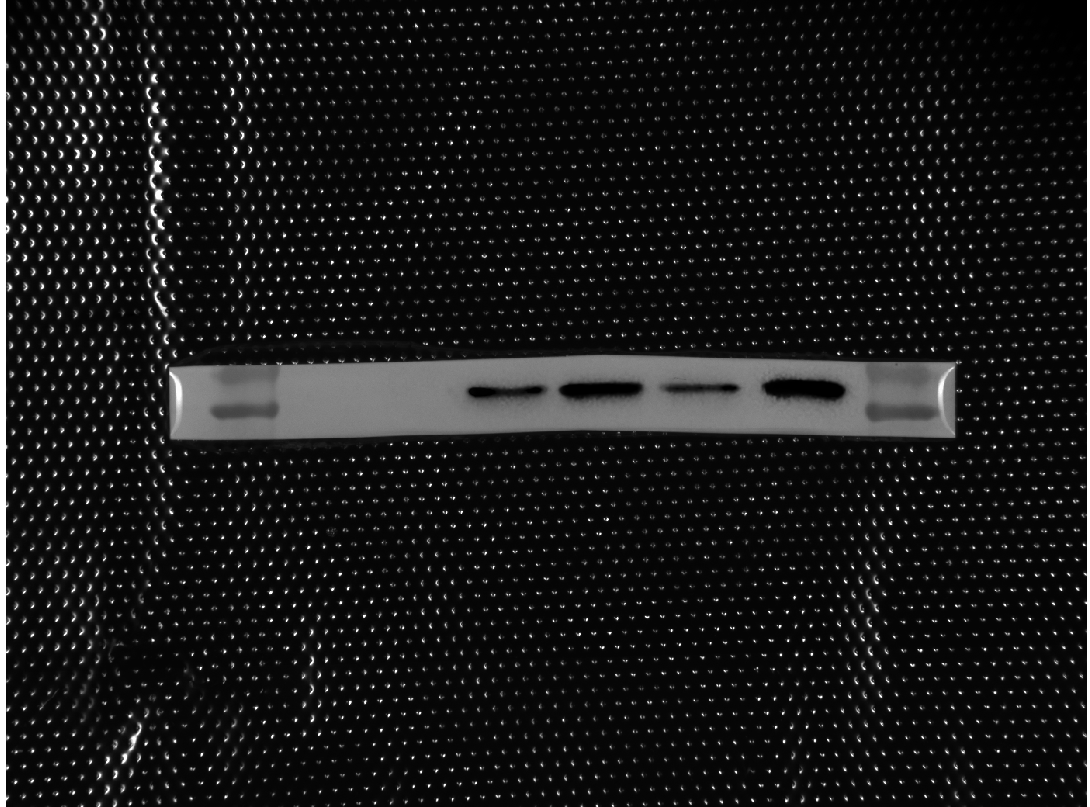

Supplement: Supplementary file 2 [file DataSheet2.zip › Suppl. Figs 8-13/8/YAP 2.tif]

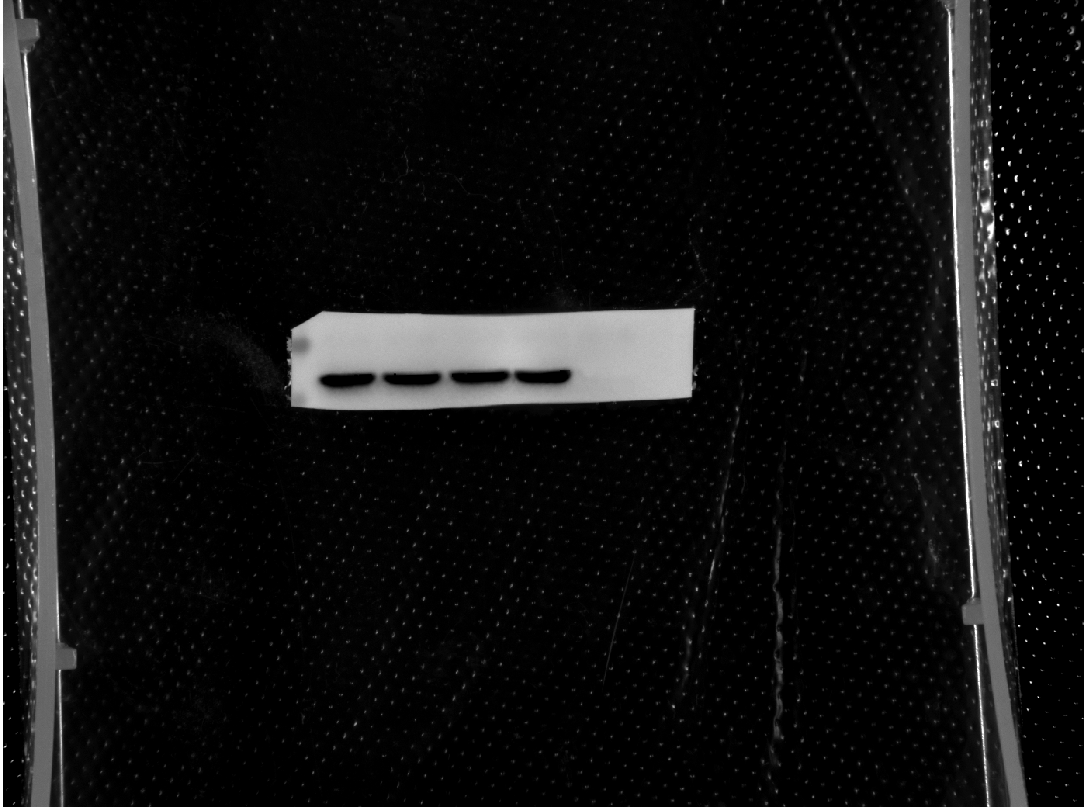

Supplement: Supplementary file 2 [file DataSheet2.zip › Suppl. Figs 8-13/8/╬▓-actin 5.tif]

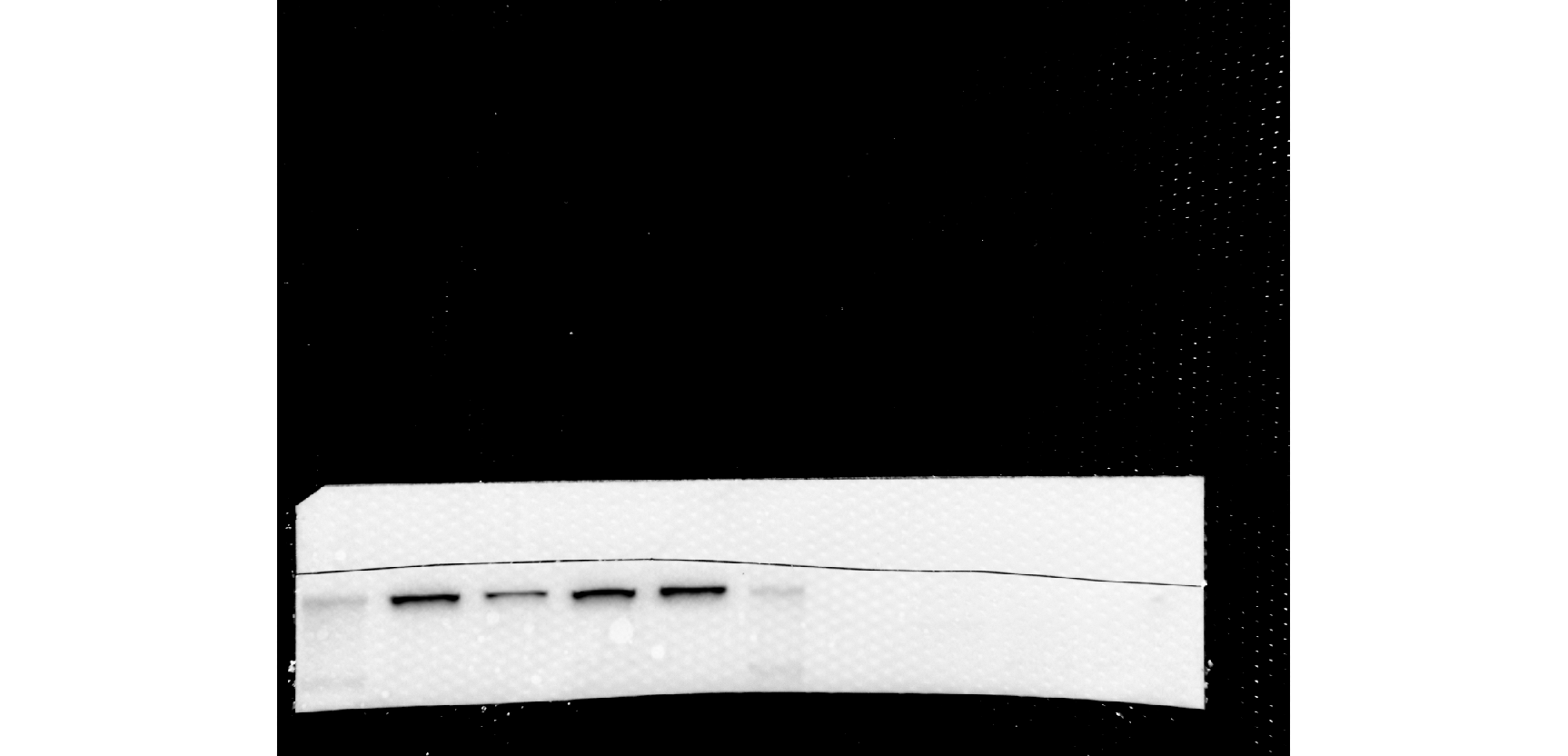

Supplement: Supplementary file 2 [file DataSheet2.zip › Suppl. Figs 8-13/12/Birc5 2.tif]

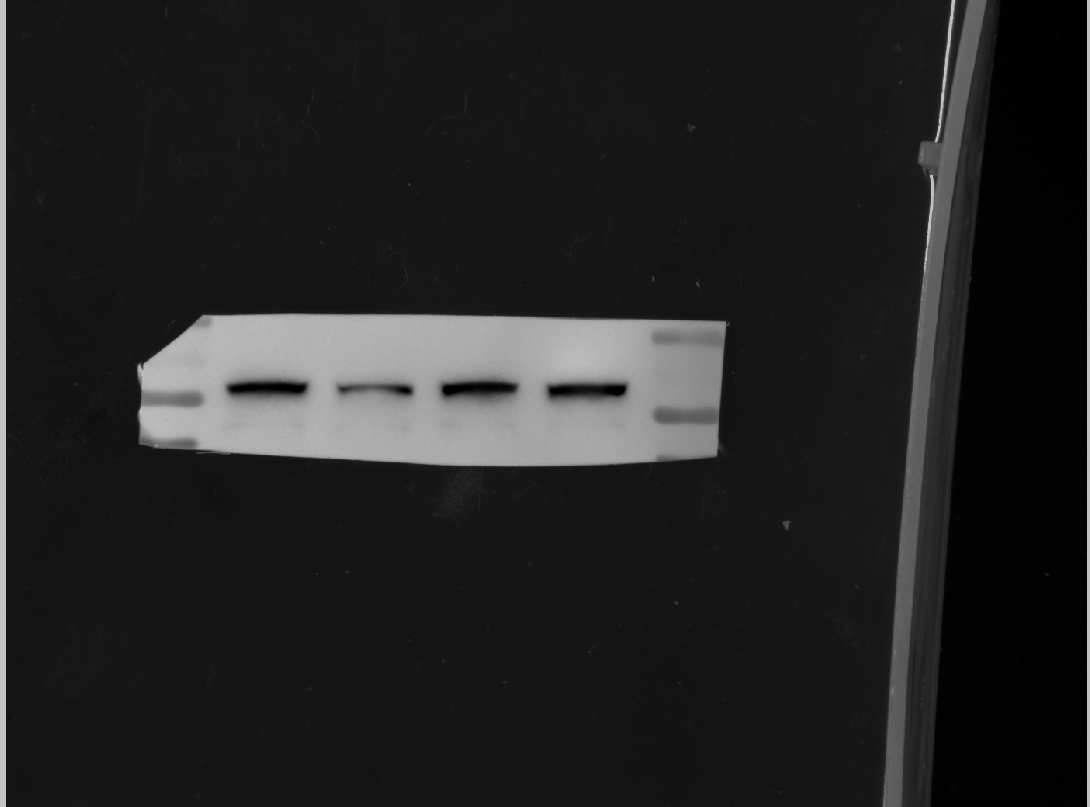

Supplement: Supplementary file 2 [file DataSheet2.zip › Suppl. Figs 8-13/12/CTGF 2.tif]

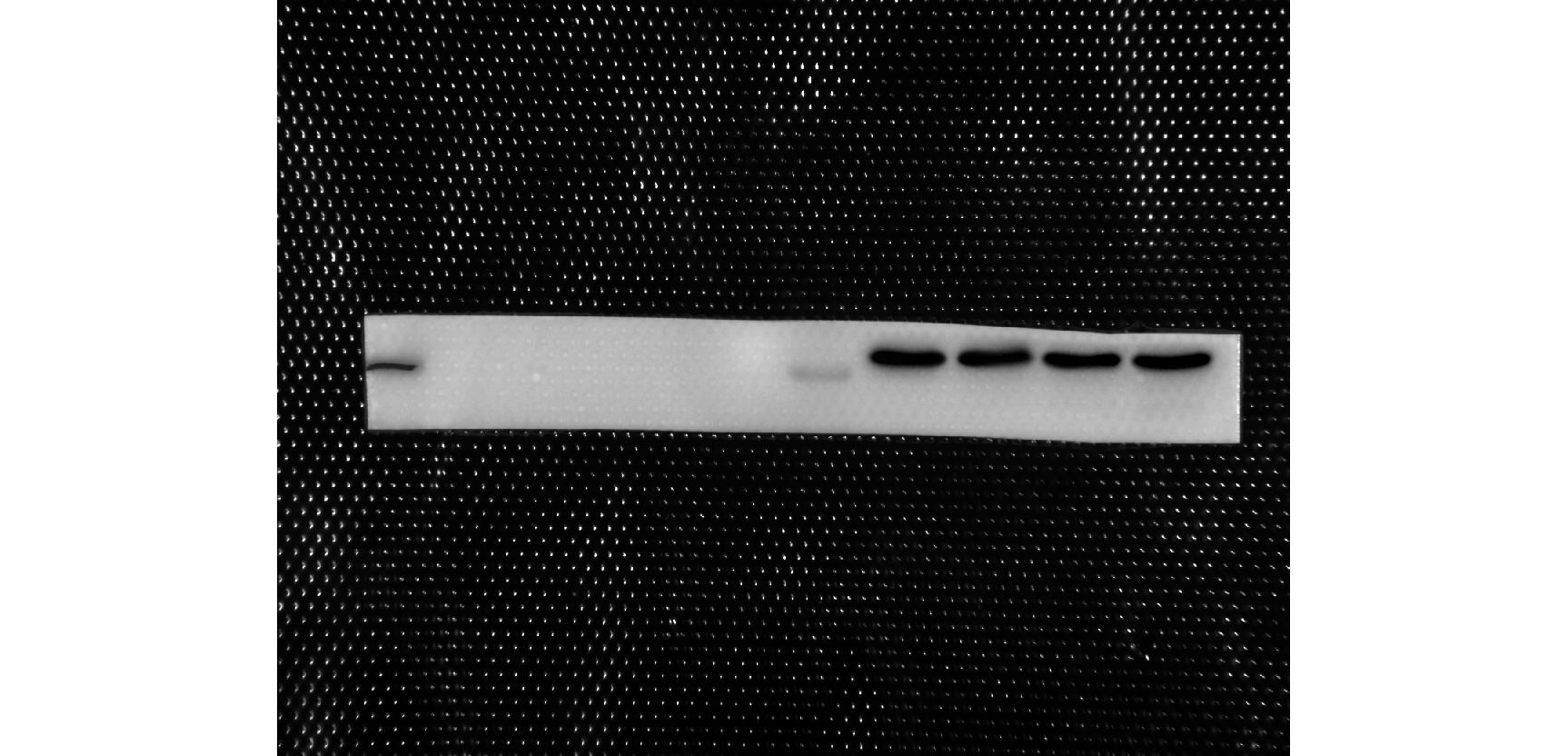

Supplement: Supplementary file 2 [file DataSheet2.zip › Suppl. Figs 8-13/12/╬▓-actin 9.tif]

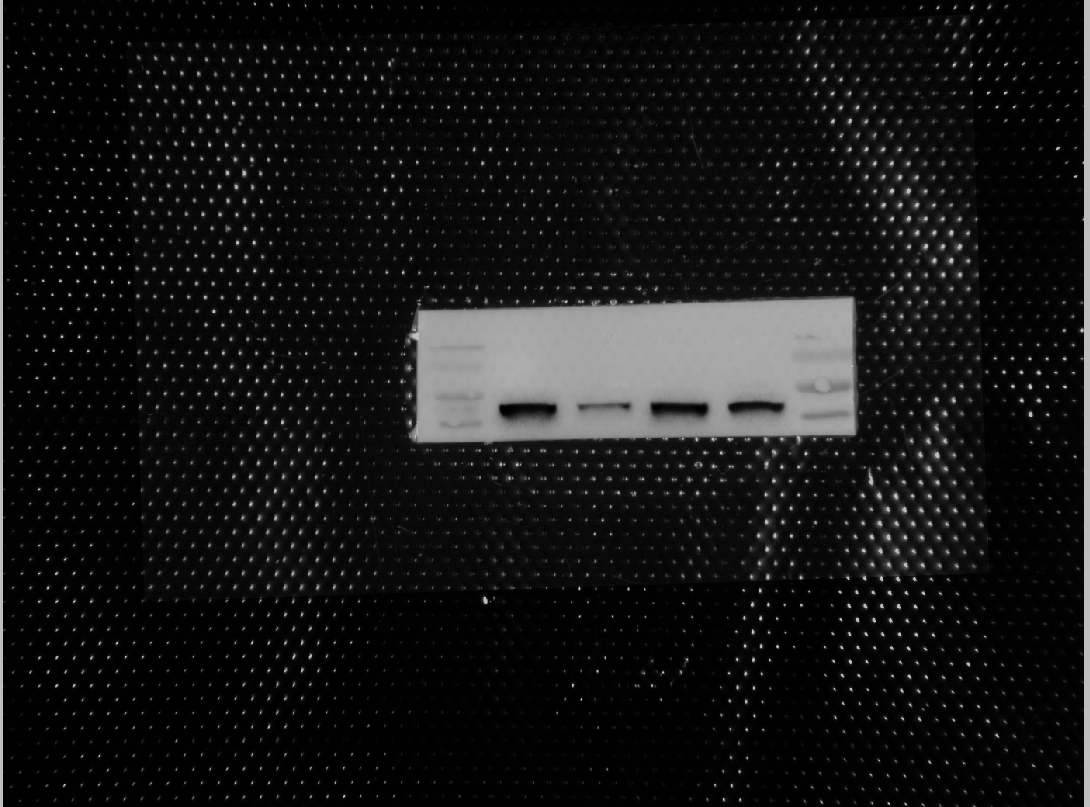

Supplement: Supplementary file 2 [file DataSheet2.zip › Suppl. Figs 8-13/12/Cyr61 2.tif]

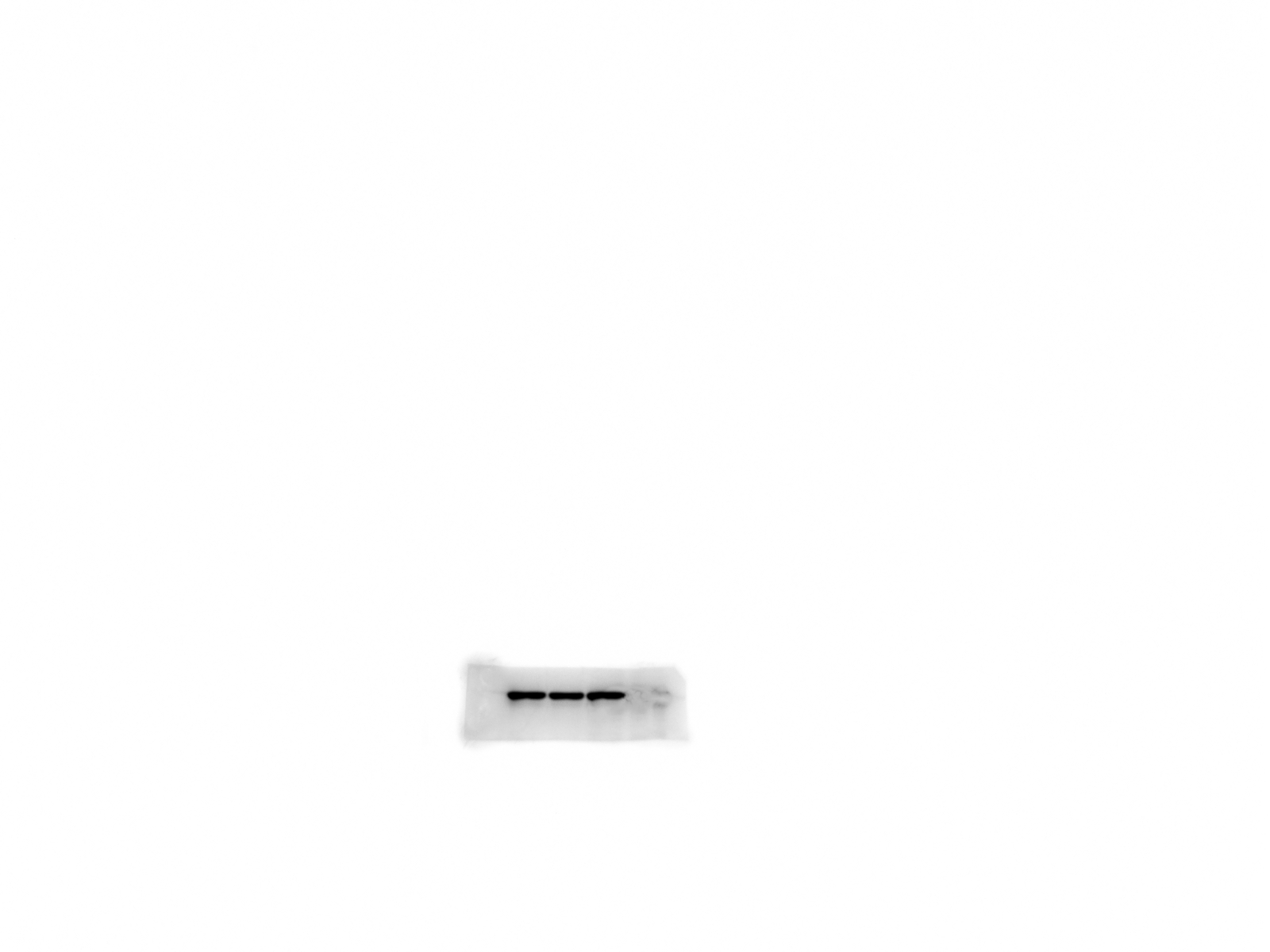

Supplement: Supplementary file 2 [file DataSheet2.zip › Suppl. Figs 8-13/13/9.tif]

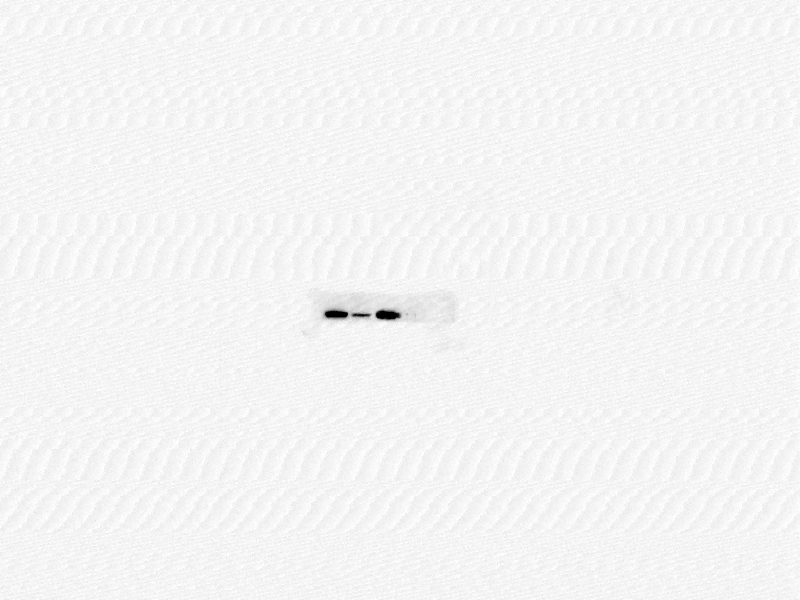

Supplement: Supplementary file 2 [file DataSheet2.zip › Suppl. Figs 8-13/13/8.tif]

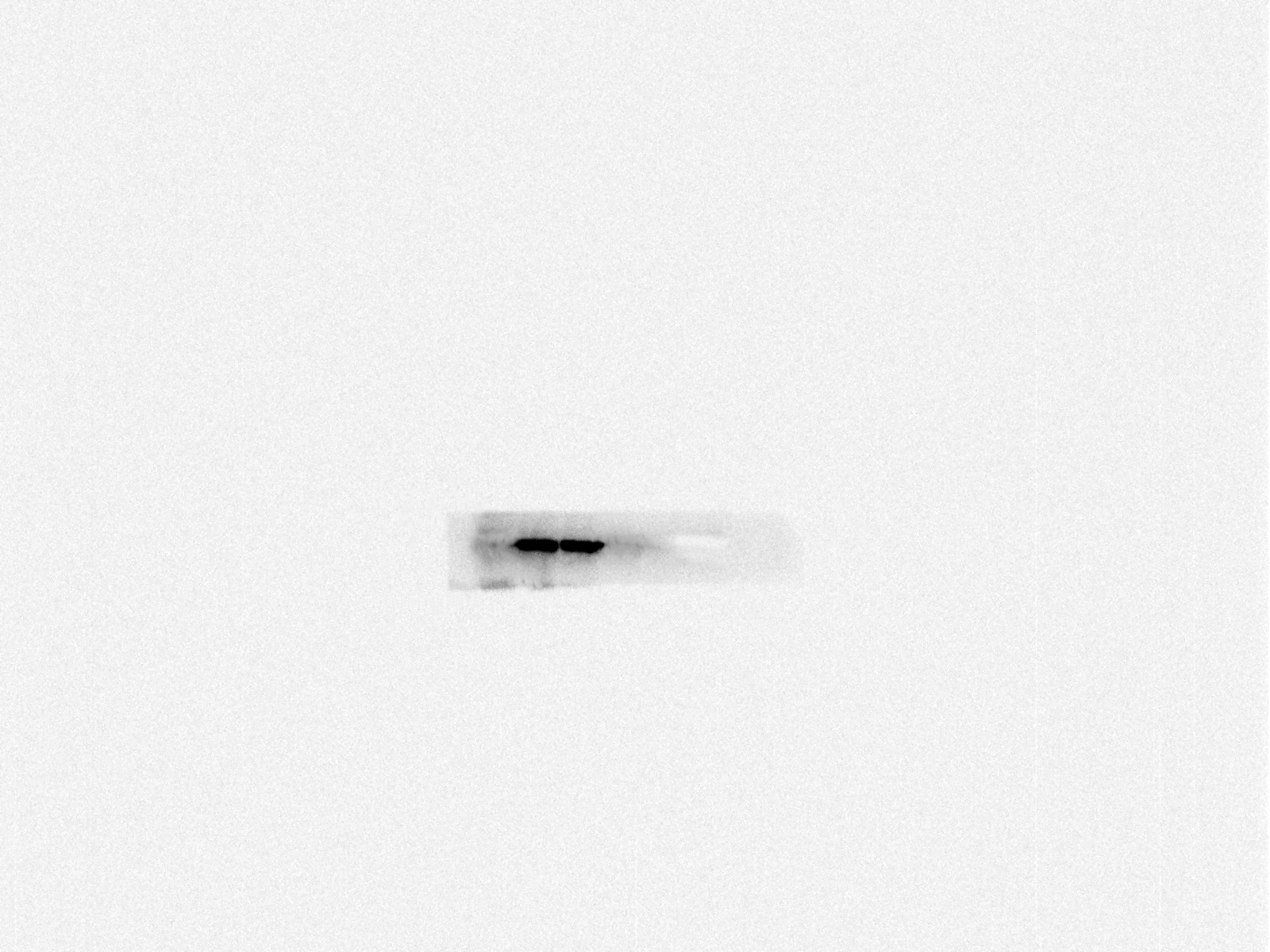

Supplement: Supplementary file 2 [file DataSheet2.zip › Suppl. Figs 8-13/13/3.tif]

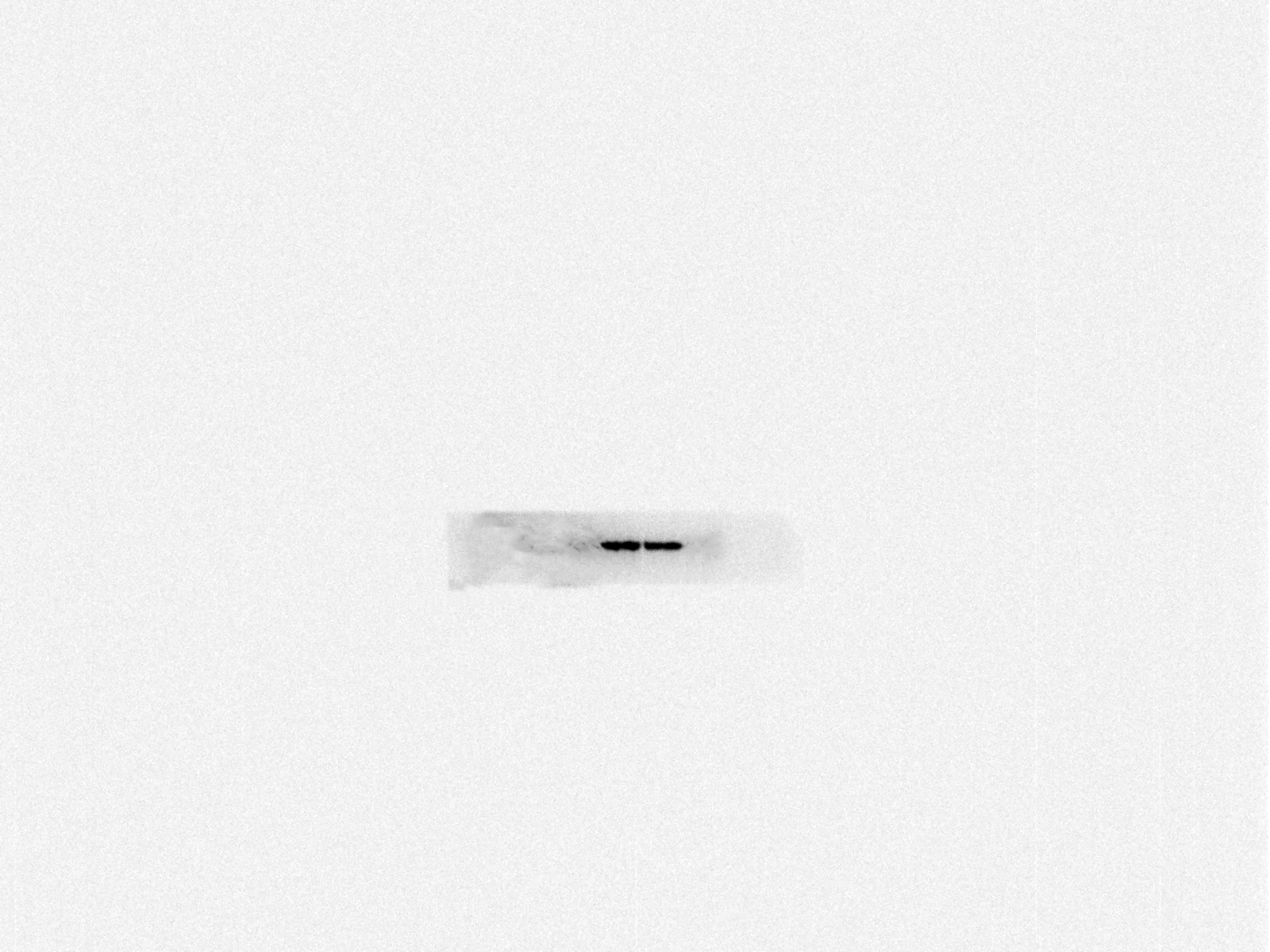

Supplement: Supplementary file 2 [file DataSheet2.zip › Suppl. Figs 8-13/13/2.tif]

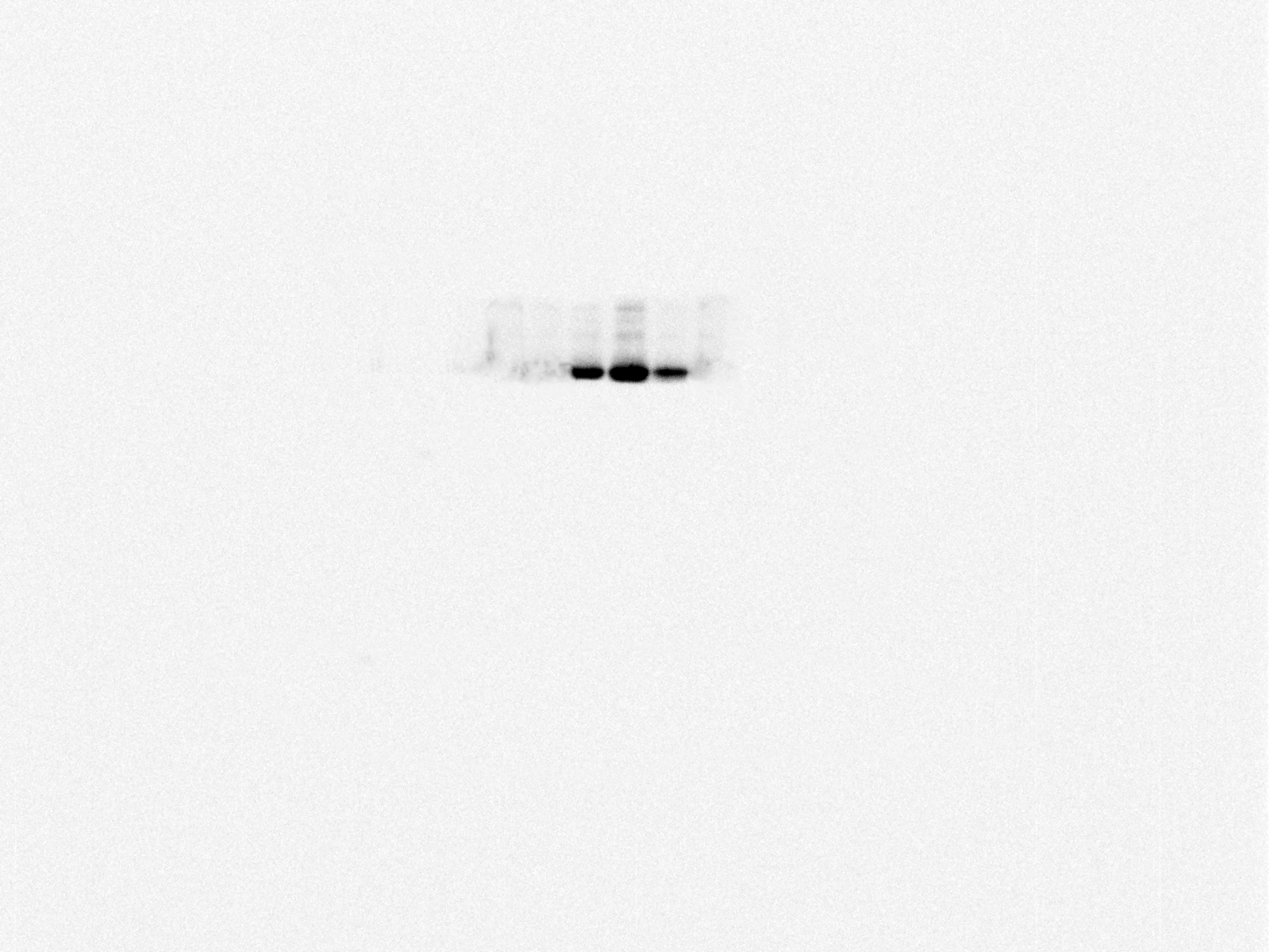

Supplement: Supplementary file 2 [file DataSheet2.zip › Suppl. Figs 8-13/13/1.tif]

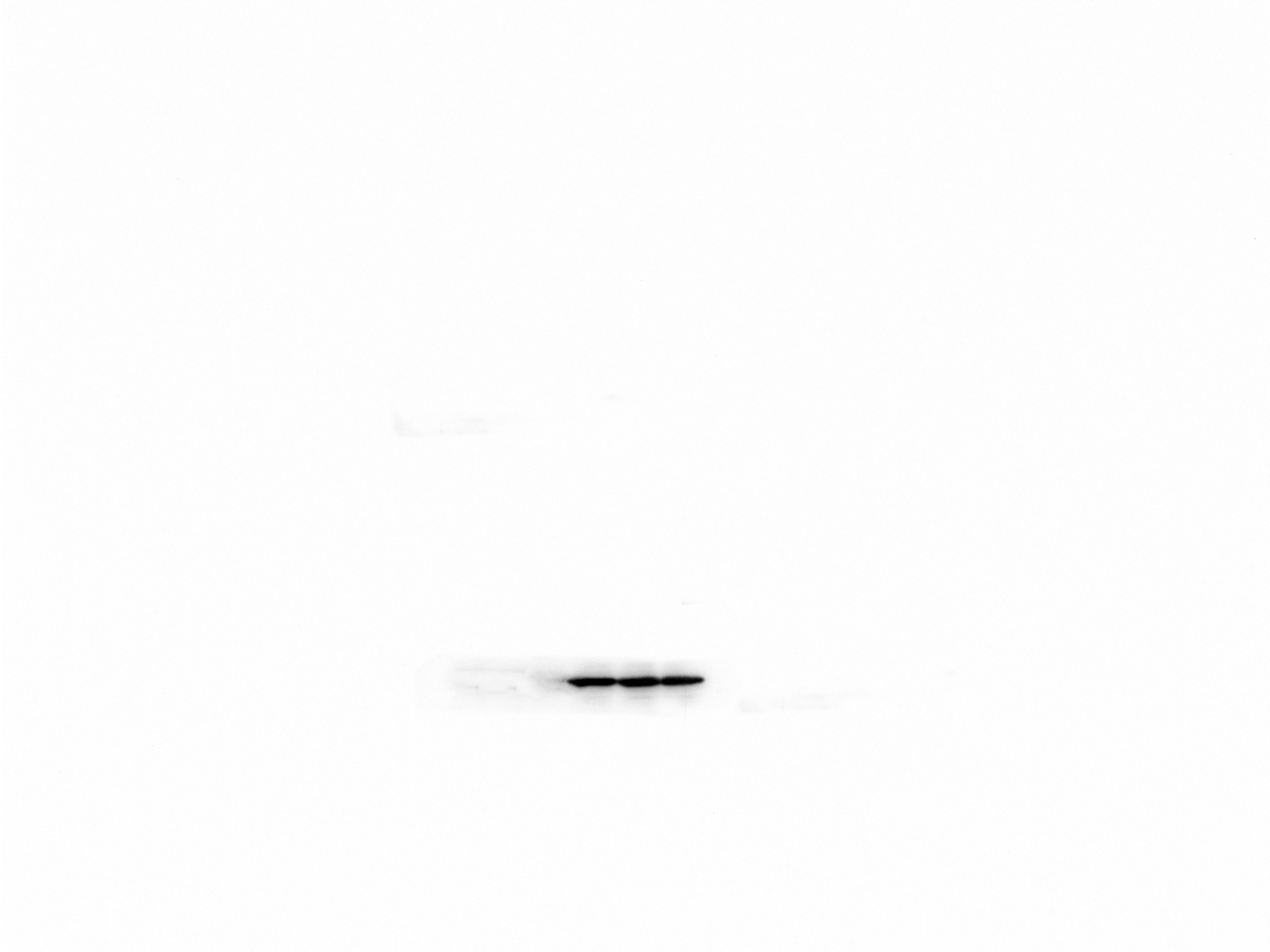

Supplement: Supplementary file 2 [file DataSheet2.zip › Suppl. Figs 8-13/13/5.tif]

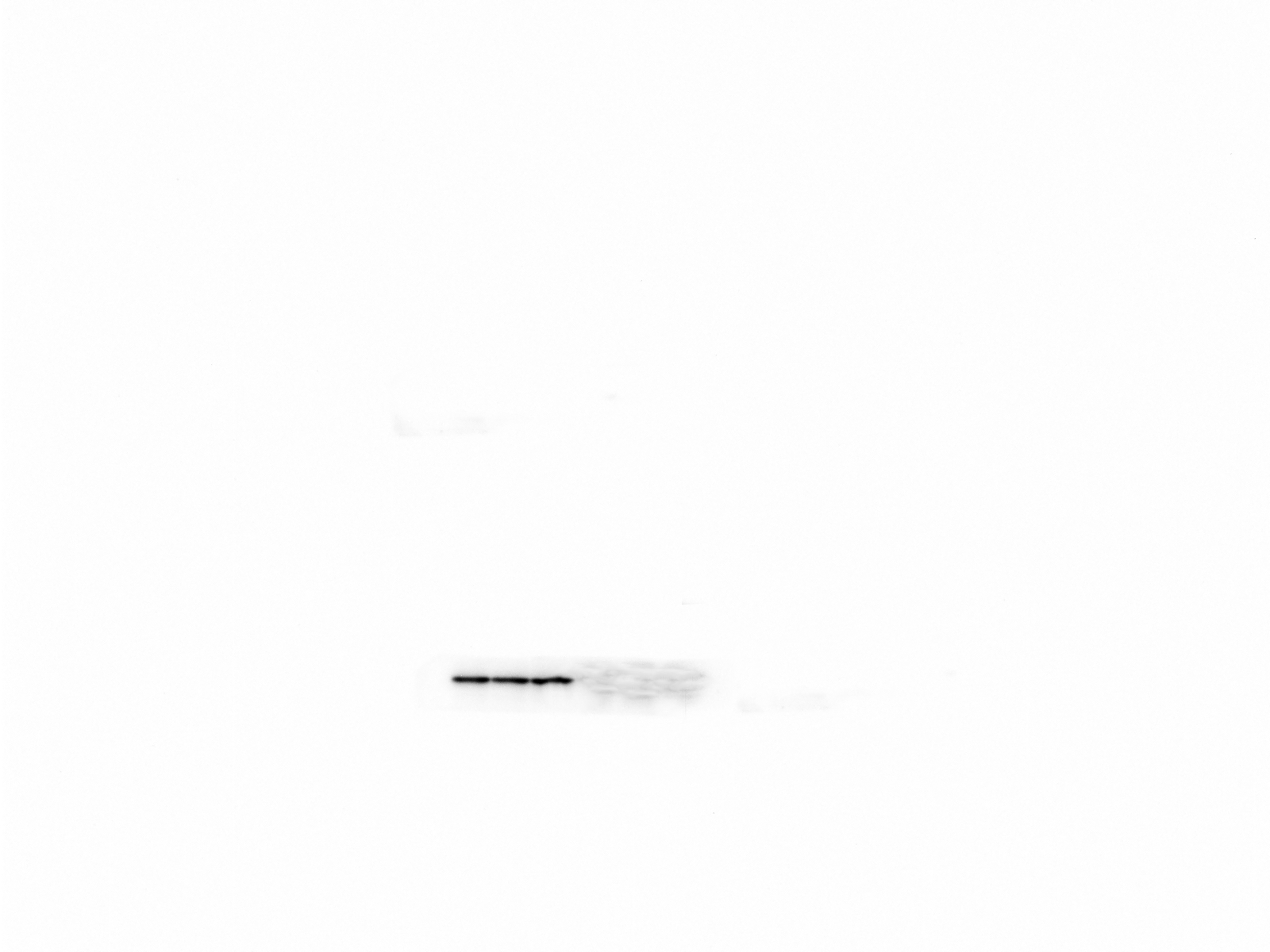

Supplement: Supplementary file 2 [file DataSheet2.zip › Suppl. Figs 8-13/13/4.tif]

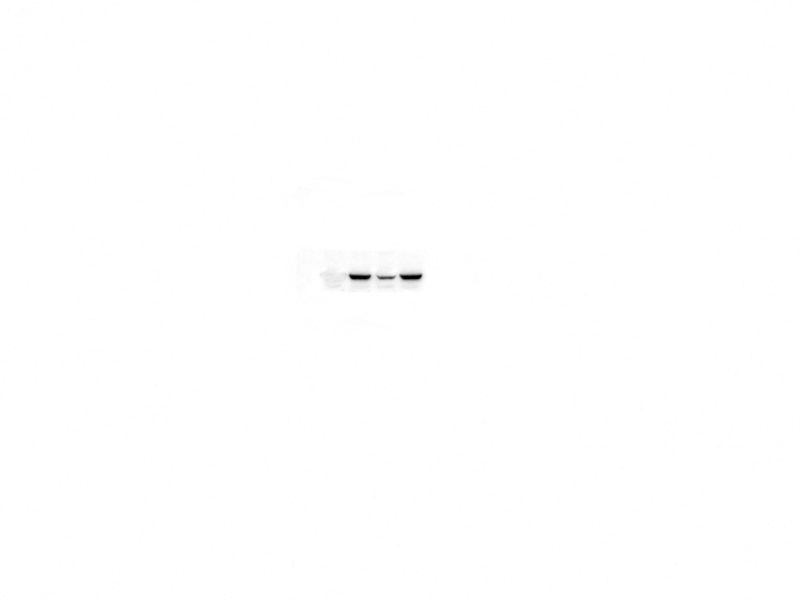

Supplement: Supplementary file 2 [file DataSheet2.zip › Suppl. Figs 8-13/13/6.tif]

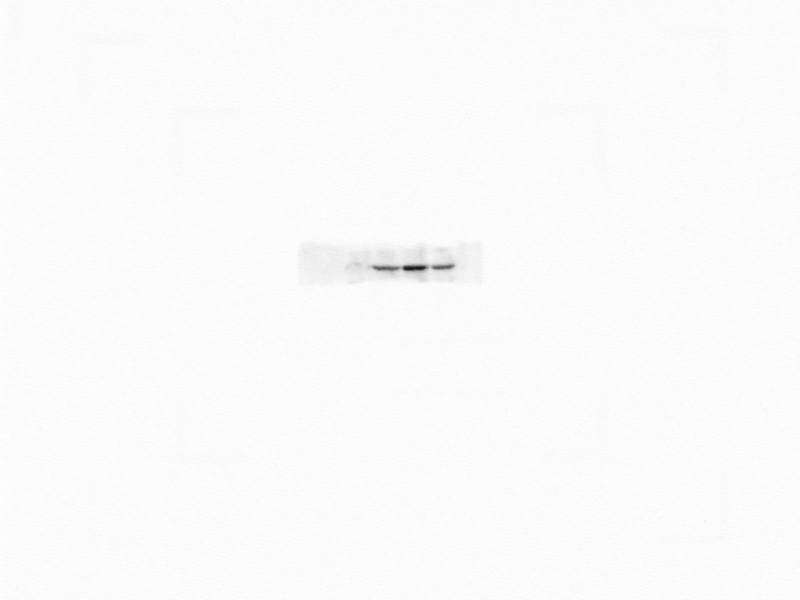

Supplement: Supplementary file 2 [file DataSheet2.zip › Suppl. Figs 8-13/13/7.tif]
